# An analysis and evaluation of the WeFold collaborative for protein structure prediction and its pipelines in CASP11 and CASP12

**DOI:** 10.1038/s41598-018-26812-8

**Published:** 2018-07-02

**Authors:** Chen Keasar, Liam J. McGuffin, Björn Wallner, Gaurav Chopra, Badri Adhikari, Debswapna Bhattacharya, Lauren Blake, Leandro Oliveira Bortot, Renzhi Cao, B. K. Dhanasekaran, Itzhel Dimas, Rodrigo Antonio Faccioli, Eshel Faraggi, Robert Ganzynkowicz, Sambit Ghosh, Soma Ghosh, Artur Giełdoń, Lukasz Golon, Yi He, Lim Heo, Jie Hou, Main Khan, Firas Khatib, George A. Khoury, Chris Kieslich, David E. Kim, Pawel Krupa, Gyu Rie Lee, Hongbo Li, Jilong Li, Agnieszka Lipska, Adam Liwo, Ali Hassan A. Maghrabi, Milot Mirdita, Shokoufeh Mirzaei, Magdalena A. Mozolewska, Melis Onel, Sergey Ovchinnikov, Anand Shah, Utkarsh Shah, Tomer Sidi, Adam K. Sieradzan, Magdalena Ślusarz, Rafal Ślusarz, James Smadbeck, Phanourios Tamamis, Nicholas Trieber, Tomasz Wirecki, Yanping Yin, Yang Zhang, Jaume Bacardit, Maciej Baranowski, Nicholas Chapman, Seth Cooper, Alexandre Defelicibus, Jeff Flatten, Brian Koepnick, Zoran Popović, Bartlomiej Zaborowski, David Baker, Jianlin Cheng, Cezary Czaplewski, Alexandre Cláudio Botazzo Delbem, Christodoulos Floudas, Andrzej Kloczkowski, Stanislaw Ołdziej, Michael Levitt, Harold Scheraga, Chaok Seok, Johannes Söding, Saraswathi Vishveshwara, Dong Xu, Foldit Players consortium, Ahmet Caglar, Alan Coral, Alison MacMillan, Allen Lubow, Barbara Failer, Bruno Kestemont, Catherine R. Landers, Charles Robert Painter, Christophe Garnier, Claudine Sellin, Dietmar Janz, Douglas Craig Wheeler, Vera Simon, Dylan M. Flear, Emmanuel Croze, George Victor McIlvaine, Gil Beecher, Gordon Lawrie, Guy Ykman, Harald Feldmann, Heidemarie K. Fuentes, Hosokawa Terumasa, Istvan Kovanecz, James Absalom Longino, Jan Hendrik Nijland, Jasper A. Diderich, Jeffrey M. Canfield, Jesper Eriksson, Jesse David Slone, Joan Goldsworthy Appel, Joanne Mitchell, John Mitch, Jorn Loots-Boiy, June M. Brownlee, Karsten Wilson, Keith T. Clayton, Kenneth E. DeFord, Kirk J. Abbey, Larry Withers, Linda Wei, Lloyd Ives, Lori A. Miller, Lynn Carpenter, Manasa G. Sharma, Manuel Ricci, Mark Stewart Binfield, Matthew John Davids, Matthias Gaebel, Michael D. Cassidy, Michael Fagiola, Michael Pfützenreuter, Nova Barlow, Peter John Triggiani, Renton Braden Mathew Innes, Robert Leduc, Rodrigo Luccas Corrêa dos Santos Gomes, Rosemarie L. R. Morneau, Scott J. Zaccanelli, Susan C. Kleinfelter, T. J. A. van der Laan, Thomas Bausewein, Thomas J. George, Varichev Mikhail, Walter Barmettler, Silvia N. Crivelli

**Affiliations:** 1 Department of Computer Science, Ben Gurion University of the Negev, Be’er sheva, Israel; 2 Biomedical Sciences Division, School of Biological Sciences, University of Reading, Reading, RG6 6AS UK; 3 Division of Bioinformatics, Department of Physics, Chemistry, and Biology, Linköping University, Linköping, Sweden; 4 Department of Chemistry, College of Science, Purdue University, West Lafayette, IN USA; 5 Purdue Institute for Drug Discovery, Purdue University, West Lafayette, IN USA; 6 Purdue Center for Cancer Research, Purdue University, West Lafayette, IN USA; 7 Purdue Institute for Inflammation, Immunology and Infectious Disease, Purdue University, West Lafayette, IN USA; 8 Purdue Institute for Integrative Neuroscience, Purdue University, West Lafayette, IN USA; 9 Department of Electrical Engineering and Computer Science, University of Missouri, Columbia, MO USA; 10 Department of Computer Science and Software Engineering, Auburn University, Auburn, AL USA; 11 Lawrence Berkeley National Laboratory, Berkeley, CA USA; 12 Laboratory of Biological Physics, Faculty of Pharmaceutical Sciences at Ribeirão Preto, University of São Paulo, São Paulo, Brazil; 13 Molecular Biophysics Unit and IISC Mathematics Initiative, Indian Institute of Science, Bangalore, India; 14 Institute of Mathematical and Computer Sciences, University of São Paulo, São Paulo, Brazil; 15 Research and Information Systems, LLC, Carmel, IN USA; 16 Department of Biochemistry and Molecular Biology, IU School of Medicine, Indianapolis, IN USA; 17 Batelle Center for Mathematical Medicine, The Research Institute at Nationwide Children’s Hospital, Columbus, OH USA; 18 Faculty of Chemistry, University of Gdansk, Gdańsk, Poland; 19 School of Engineering, University of California, Merced, CA USA; 20 Department of Chemistry, Seoul National University, Seoul, Republic of Korea; 21 Department of Computer and Information Science, University of Massachusetts Dartmouth, MA, USA; 22 Department of Chemical and Biological Engineering, Princeton University, Princeton, NJ USA; 23 Texas A&M Energy Institute, Texas A&M University, College Station, TX USA; 24 Department of Biochemistry, University of Washington, Seattle, WA USA; 25 Howard Hughes Medical Institute, University of Washington, Seattle, WA USA; 26 School of Computer Science and Information Technology, NorthEast Normal University, Changchun, China; 27 Christopher S. Bond Life Sciences Center, University of Missouri, Columbia, MO USA; 28 Max Planck Institute for Biophysical Chemistry, Göttingen, Germany; 29 California State Polytechnic University, Pomona, CA USA; 30 Artie McFerrin Department of Chemical Engineering, Texas A&M University, College Station, TX USA; 31 Institute for Protein Design, University of Washington, Seattle, WA USA; 32 Baker Laboratory of Chemistry and Chemical Biology, Cornell University, Ithaca, NY USA; 33 Department of Computational Medicine and Bioinformatics, University of Michigan, Ann Arbor, MI USA; 34 Interdisciplinary Computing and Complex BioSystems (ICOS) research group, School of Computing, Newcastle University, Newcastle-upon-Tyne, UK; 35 Intercollegiate Faculty of Biotechnology, University of Gdańsk and Medical University of Gdańsk, Gdańsk, Poland; 36 Center for Game Science, Department of Computer Science & Engineering, University of Washington, Seattle, WA USA; 37 College of Computer and Information Science, Northeastern University, Boston, MA USA; 38 Department of Structural Biology, School of Medicine, Stanford University, Stanford, CA USA; 39 Department of Computer Science, University of California, Davis, CA USA; 40 Rosetta Commons, University of Washington, Seattle, Washington 98195 USA

## Abstract

Every two years groups worldwide participate in the Critical Assessment of Protein Structure Prediction (CASP) experiment to blindly test the strengths and weaknesses of their computational methods. CASP has significantly advanced the field but many hurdles still remain, which may require new ideas and collaborations. In 2012 a web-based effort called WeFold, was initiated to promote collaboration within the CASP community and attract researchers from other fields to contribute new ideas to CASP. Members of the WeFold coopetition (cooperation and competition) participated in CASP as individual teams, but also shared components of their methods to create hybrid pipelines and actively contributed to this effort. We assert that the scale and diversity of integrative prediction pipelines could not have been achieved by any individual lab or even by any collaboration among a few partners. The models contributed by the participating groups and generated by the pipelines are publicly available at the WeFold website providing a wealth of data that remains to be tapped. Here, we analyze the results of the 2014 and 2016 pipelines showing improvements according to the CASP assessment as well as areas that require further adjustments and research.

## Introduction

The current experimental approaches to determine the native structure of proteins are too costly to keep pace with the wealth of protein sequences that genome sequencing projects generate. As of October 2017, the UniProt/TrEMBL database^1^ contains 93,236,986 protein sequence entries, whereas the protein data bank^2^ contains only 134,656 experimentally-determined protein structures, of which 42,591 are unique. A reliable method for predicting protein structure from its primary sequence of amino acids could help to bridge the sequence-structure knowledge gap and have a significant impact on bioinformatics, biology, and medicine. To help advance and assess the protein structure prediction (PSP) field, the Critical Assessment of techniques for protein Structure Prediction (CASP)^3–6^ series of community-wide experiments was initiated in 1994. Every other year, CASP challenges its participants to submit predicted structures for around one hundred proteins, whose structures are about to be experimentally determined or have been determined but not yet published. The CASP experiments run for three months and the results are evaluated by independent assessors after the experimental structures are made available. CASP history over the last two decades indicates that while significant progress has been made^4–6^, major roadblocks still remain^5,6^.

**Figure 1:**
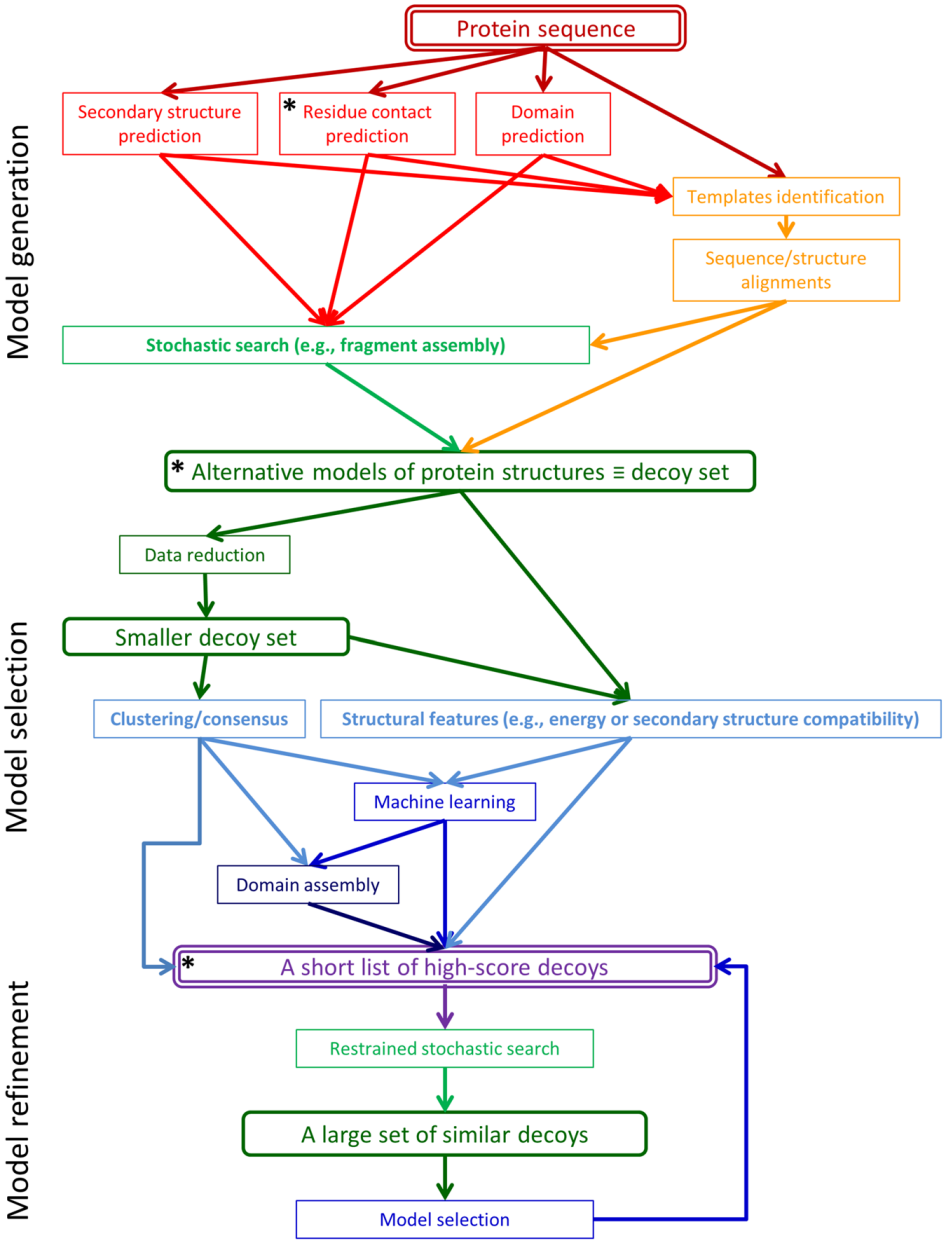
A schematic depiction of the multi-step and multi-path information flow of protein structure prediction. Rounded rectangles represent information and plain rectangles represent basic tasks, each of which is an open computational problem. A prediction process starts with a protein sequence, passes at least once through a set of decoys (structural models of proteins), and ends with a short list, ideally one, of high score decoys. The paths in this graph are not mutually exclusive.

One such roadblock is the multi-step nature of PSP, and the diversity of the approaches to these steps. Figure 1 depicts this complexity in the form of a directed graph. A method for structure prediction needs to implement at least one path that leads from a protein sequence to a few high scoring structural models of proteins, aka *decoys*. The final overall performance of a prediction protocol depends heavily on the quality of the intermediate steps (the rectangle nodes in Fig. 1), each of which is still an open scientific problem. Thus, progress in the PSP field depends on advances in all sub-problems. Yet the need to build or at least adopt a complete path in order to participate in CASP raises a high entry barrier for new people and ideas. Further, the interfaces between the various steps (the arrows in Fig. 1) are not always standardized, making it difficult to exchange elements between existing methods.

The CASP organizers have long recognized this problem, and CASP experiments include three tracks that serve as short-cut entries into the graph (marked by asterisks in Fig. 1). Indeed, these tracks: contact prediction, quality assessment, and refinement, each addressing a major sub-problem, have a considerable impact on research. They evaluate the performance of methods in an objective manner, and most importantly, they provide developers with large data sets that can be used to improve them. Yet, these CASP tracks offer only a limited solution. Many sub problems are not covered at all, and further, the treatment of quality assessment and refinement is somewhat artificial. Within a prediction pipeline, for example, quality assessment is typically applied to large sets of decoys, all of which were generated by the same method (i.e., previous steps of the pipeline). The CASP quality assessment decoys, contrarily, are far fewer and are the outcome of dozens of servers, employing diverse methods. Similarly, refinement tasks in CASP start from a single decoy, allowing the use of CPU intensive methods. Contrarily, a prediction pipeline may require the refinement of all top scoring decoys, which limits the available CPUs per refinement task.

**Figure 2:**
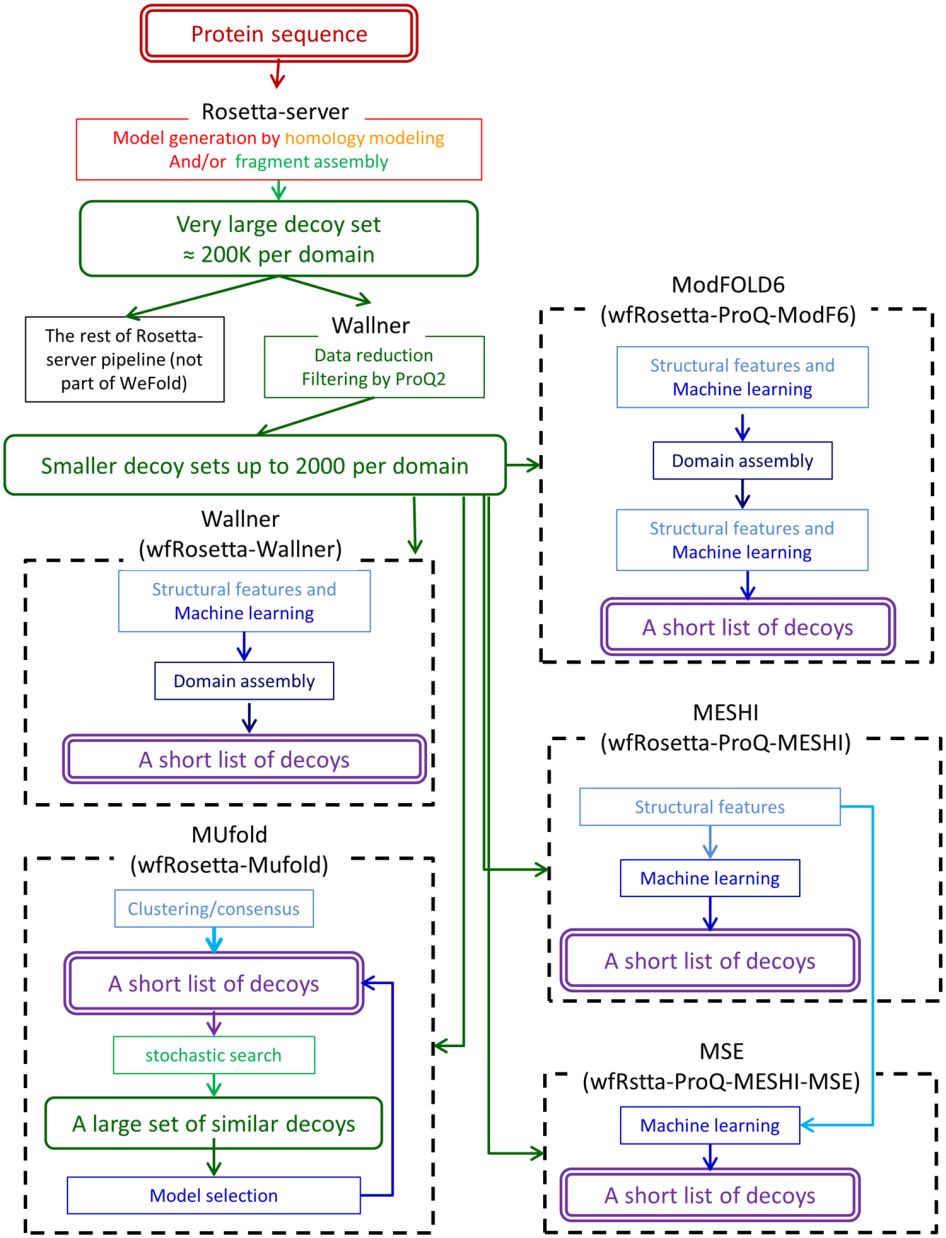
An illustration of the WeFold pipeline concept. The figure presents a schematic depiction of 5 WeFold3 pipelines, which share their first components and differ in the final stages. Graph representation and colors are based on Fig. 1. A complete list of all the WeFold2 and WeFold3 pipelines is presented in Table 1 and in the main text.

In order to support method development and reduce entry barriers, we started the WeFold collaborative effort in 2012^7–9^. WeFold provides a flexible infrastructure for the creation of prediction pipelines (e.g. Fig. 2 shows the pipelines that start with Rosetta decoys), into which researchers may insert components of their methods such as refinement and quality assessment. These pipelines participate as groups in CASP allowing their overall performance to be evaluated in an objective and coherent manner along with all the other groups. This way a method may be applied to a variety of input sources and the utility of its outcome may be tested within a variety of pipelines. Further, the entire information flow through this infrastructure is documented, resulting in a data source for the development of methods that tackle sub-problems of PSP. Yet, an infrastructure needs a community of users to accomplish its goals. To this end, WeFold pursues an inclusive approach that brings together different groups that already participate in CASP, reaches out to raise awareness and excitement outside the CASP community, and tries to act as an incubator for new ideas. In fact, we have recruited non-CASP members who have contributed to the WeFold3 efforts and are co-authors of this manuscript or are working on innovative methodologies for the upcoming CASP13 exercise^10,11^.

The first WeFold collaboration included 31 researchers from 12 institutions. We created 5 hybrid pipelines, each of which was composed of a combination of methods contributed by the participants^7^. These pipelines competed against all the other methods during the CASP10 experiment. Motivated by the success of the first WeFold experiment, a broader invitation was extended in 2014 to both CASP and non-CASP members. This invitation was well received and resulted in the participation of 21 groups in CASP11. Finally, the last WeFold experiment included 16 groups that participated in 12 pipelines, which competed in CASP12 (2016). In this paper, we refer to these events as WeFold1, WeFold2, and WeFold3, respectively.

This manuscript analyses WeFold2 and WeFold3 as the latest two successive case studies, shows an overall improvement of the latter, and identifies the pipelines that stood out. The first case study helped in shaping the more successful second one, and the second provides plausible guidelines for future efforts. To this end, we complement the CASP assessment with analysis of the information flow within the pipelines to figure out where we benefited from joining forces and where we could have designed the pipelines better (the Supplementary Materials provide extensive analysis of the pipelines steps for WeFold2 and WeFold3). Some of these issues have been discussed intensively before (e.g. scoring) and others have almost been ignored in the literature, most notably domain assembly. Finally, we discuss the data resources that WeFold offers to the PSP community.

## Methods

The WeFold2 project participated in two categories within the CASP experiment: (1) PSP and (2) model refinement. Groups that participated in WeFold2 contributed one or more pipeline components, which were combined into 23 different pipelines. These components included: four major decoy generators (Foldit, Zhang, UNRES, and CASP11 servers^12–14^), three contact-prediction methods (GREMLIN, ICOS, and Floudas^15,16^), one secondary structure prediction method (conSSert^17^), a clustering algorithm adapted to deal with large numbers of models (Wallner), and other clustering algorithms such as the minimum-variance algorithm^18^, one filtering algorithm (Wallner), five refinement methods (Princeton_TIGRESS, KoBaMIN, GalaxyRefine, Delbem, and 3Drefine^19–23^) and eight QA/selection algorithms (SVLab, APOLLO, ModFOLD5_single, ProQ2, Delbem, Seder1.0, Kloczkowski/Pawlowski, and MESHI-score^10,24–28^). The online protein structure prediction servers were an important source of models for some of the WeFold pipelines. Two CASP servers, HHpred-A and HHpred-X^29^, were explicitly members of WeFold2 and provided their predictions immediately after they were generated. The other server decoys were obtained from the CASP website^30^.

The WeFold3 project focused on the protein structure prediction category alone. Participating groups combined components into 12 different pipelines. These components included: three major model generators (Rosetta, UNRES, and CASP12 servers)^14,31,32^, two contact prediction methods (GREMLIN and Floudas^15^), one secondary structure prediction method (conSSert^17^), one clustering algorithm^18^, three refinement methods (Princeton_TIGRESS, GalaxyRefine, and 3Drefine^19,22,23^), and seven QA/selection methods (APOLLO, MESHI-score, MESHI-MSC, ModFOLD6, MUFold, ProQ2, and Seder^10,25–28,33,34^). We decided to compare QA/scoring methods fairly by applying them to the same decoys sets. Thus, *wfRosetta*-*MUfold*, *wfRosetta*-*ProQ*-*MESHI*, *wfRosetta*-*ProQ*-*MESHI*-*MSC*, *wfRosetta*-*ProQ*-*ModF6*, *wfDB_BW_SVGroup*, and *wfRosetta*-*Wallner* started with the same set of Rosetta decoys and *wfMESHI*-*Seok* and *wfMESHI*-*TIGRESS* started with the same subsets of server decoys selected by MESHI. Moreover, *wfRosetta*-*ProQ*-*MESHI* and *wfRosetta*-*ProQ*-*MESHI*-*MSC* also used the same set of decoys and features to strictly compare two scoring functions^10^. With regards to decoys reduction needed to reduce the large set of Rosetta decoys to a manageable size for refinement and QA, we replace the filtering and clustering procedure that we had used in WeFold2 for the Foldit decoys, by ProQ2.

Table 1 shows all the pipeline components and the groups that contributed to them in WeFold2 and WeFold3. Table 2 shows all the pipelines that resulted from WeFold2 and WeFold3 with their corresponding CASP11 and CASP12 group number, category, and number of targets attempted. Five of these pipelines are also presented with some detail in Fig. 2. Description of the pipelines is provided in the Supplementary Materials.

**Table 1:** Pipeline components in WeFold2 and WeFold3 and the groups that contributed.

Contribution	WeFold2	WeFold3	Group
Alignment	HHPred		Söding
Sampling	Foldit		Baker&Khatib Groups
		RosettaServer	Baker Group
	UNRES	UNRES	Scheraga&Gdansk Groups
	Zhang		Zhang Group
Contact Predictions	GREMLIN	GREMLIN	Baker Group
	Floudas	Floudas	Floudas Group
	ICOS		Jaume Bacardit
Secondary Structure Pred.	conSSert	conSSert	Floudas Group
Clustering	Wallner		Björn Wallner
	Minimum Variance	Minimum Variance	Scheraga&Gdansk Groups
Filtering	Wallner	ProQ2	Björn Wallner
Refinement	Delbem		Delbem Group
QA/Selection	KoBaMIN		Levitt Group
	GalaxyRefine	GalaxyRefine	Seok Group
	PTIGRESS	TIGRESS	Floudas Group
	3D refine	3D refine	Cheng Group
	APOLLO	APOLLO	Cheng Group
	Delbem		Delbem Group
	Kloczkowski/Pawlowski		Kloczkowski Group
	Kloczkowski/Seder	Kloczkowski/Seder	Kloczkowski Group
	MESHI-score	MESHI-score	Keasar Group
		MESHI-MSC	Mirzaei&Crivelli Group
	ModFOLD5	ModFOLD6	McGuffin Group
		MUfold	Xu Group
	ProQ2	ProQ2	Björn Wallner
	SVLab	SVLab	SVLab

**Table 2:** Pipelines formed in WeFold2 and WeFold3, with their corresponding group number (assigned by the prediction center upon registration), category (tertiary structure prediction or refinement), number of targets attempted and groups involved.

WeFold	Pipeline Name	Group #	Category	Attempted Targets	Groups Involved
WeFold2	wf-Baker-UNRES	128	TSP	13	Baker, Scheraga, Gdansk
	wfCPUNK	442	TSP	55	Floudas, Scheraga, Gdansk, Levitt
	wfKsrFdit-BW-Sk-BW	336	TSP	25	Keasar, Baker/Foldit, Wallner, Seok
	wfKsrFdit-BW-Sk-McG	120	TSP	27	Keasar, Baker/Foldit, Wallner, Seok, McGuffin
	wfZhng-Ksr	173	TSP	25	Zhang, Keasar
	wfZhng-Sk-BW	260	TSP	27	Zhang, Seok, Wallner
	wfAll-Cheng	403	TSP	45	All WeFold Groups, Cheng
	wfAll-MD-RFLB	153	TSP	46	All WeFold Groups, Delbem
	wfMix-KFa	118	TSP	55	Baker/Foldit, Kloczkowski/Faraggi
	wfMix-KFb	197	TSP	55	Baker/Foldit, Kloczkowski/Faraggi
	wfMix-KPa	482	TSP	49	Baker/Foldit, Kloczkowski/Pawlowski
	wfMix-KPb	056	TSP	49	Baker/Foldit, Kloczkowski/Pawlowski
	wfHHpred-PTIGRESS	034	TSP	55	Söding, Floudas
	wfKeasar-PTIGRESS	457	TSP	43	Keasar, Floudas
	wf-AnthropicDreams	203	TSP	27	Keasar, Baker/Foldit
	WeFold-Contenders	014	TSP	24	Keasar, Baker/Foldit
	WeFold-GoScience	433	TSP	27	Keasar, Baker/Foldit
	WeFold-Wiskers	281	TSP	7	Keasar, Baker/Foldit
	wf-Void_Crushers	258	TSP	27	Keasar, Baker/Foldit
	wfFdit-BW-KB-BW	208	Refinement	22	Baker/Foldit, Wallner, Levitt
	wfFdit-K-McG	180	Refinement	23	Baker/Foldit, Wallner, Levitt, McGuffin
	wfFdit_BW_K_SVGroup	154	Refinement	15	Baker/Foldit, Wallner, Levitt, SVLab
	wfFdit_BW_SVGroup	334	Refinement	17	Baker/Foldit, Wallner, SVLab
WeFold3	wf-BAKER-UNRES	300	TSP	16	Baker, Scheraga, Gdansk
	wfCPUNK	182	TSP	47	Floudas, Scheraga, Gdansk, Levitt
	wfDB_BW_SVGroup	475	TSP	46	Baker, Wallner, SVLab
	wfRosetta-MUfold	325	TSP	64	Baker, Wallner, Xu
	wfRosetta-ProQ-MESHI	173	TSP	59	Baker, Wallner, Keasar
	wfRosetta-ProQ-ModF6	252	TSP	58	Baker, Wallner, McGuffin
	wfRosetta-Wallner	456	TSP	56	Baker, Wallner
	wfRstta-PQ2-Seder	067	TSP	85	Baker, Wallner, Kloczkowski/Faraggi
	wfRstta-PQ-MESHI-MSC	441	TSP	55	Baker, Wallner, Keasar, Mirzaei
	wfAll-Cheng	239	TSP	77	All WeFold Groups, Cheng
	wfMESHI-Seok	384	TSP	65	Keasar, Seok
	wfMESHI-TIGRESS	303	TSP	61	Keasar, Floudas

TSP is Tertiary Structure Prediction.

### Pairwise pipeline comparison

Table 2 shows that many WeFold2 pipelines failed to submit decoys to all or even most of the targets. This was mainly true for the pipelines that relied on decoy sets contributed by the Foldit Players and the Zhang group. Based on volunteering work of citizen scientists, the Foldit project could not cope with the rate of target release during the CASP folding season. Further, the computational resources of players could not support modeling of the larger targets. Thus, the Foldit teams provided decoy datasets for less than a half of CASP “human” targets. On the other hand the decoy set contribution of the Zhang group included only single domain targets, again accounting for less than half of the human targets. To complicate the analysis further, the various submitted subsets of the targets did not overlap. Target coverage has improved considerably in WeFold3 when the pipelines attempted a larger number of the targets as shown in Table 2.

The CASP evaluation of performance, justifiably, takes a “user’s” perspective and penalizes lack of coverage. Yet, from a developer’s perspective, if these incomplete datasets were simply dismissed, we would miss much that could be learned to promote further research into such collaborative pipelines. Indeed, a thorough analysis of the WeFold2 results proved informative and helped shape the more successful WeFold3 pipelines (please refer to Supplementary Materials). We envisage that further improvements can be gained based on the current analyses presented below.

### Data availability

All the protein models contributed to WeFold2 and WeFold3 or generated by WeFold pipelines are available at https://wefold.nersc.gov/wordpress/casp11/downloads/ and https://wefold.nersc.gov/wordpress/casp12/downloads/ respectively.

## Results and Discussion

### WeFold2 and WeFold3 performances in tertiary structure prediction

**Figure 3:**
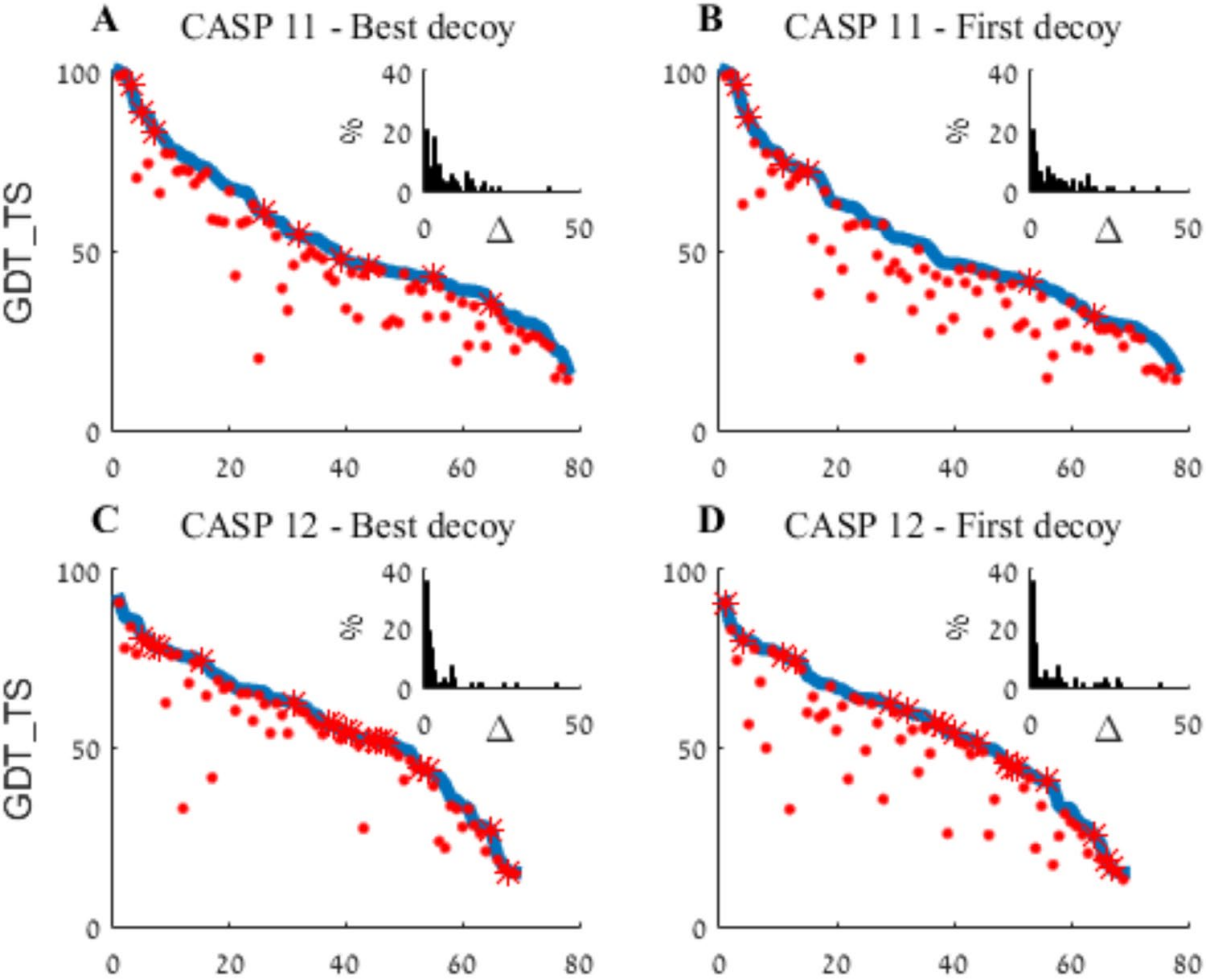
Aggregated best models WeFold vs. all CASP groups. In each panel, targets are sorted in descending order of the best decoy submitted (blue line). The best WeFold decoy for each target is marked by a red dot or, when coincides with the overall best, red asterisk. The insert histograms depict the distributions of quality differences (Δ) between the best decoys and their corresponding best WeFold decoy. (**A** and **B**) – CASP11; (**C** and **D**) – CASP12; (**A** and **C**) – Best out of five; (**B** and **D**) – First model.

**Figure 4:**
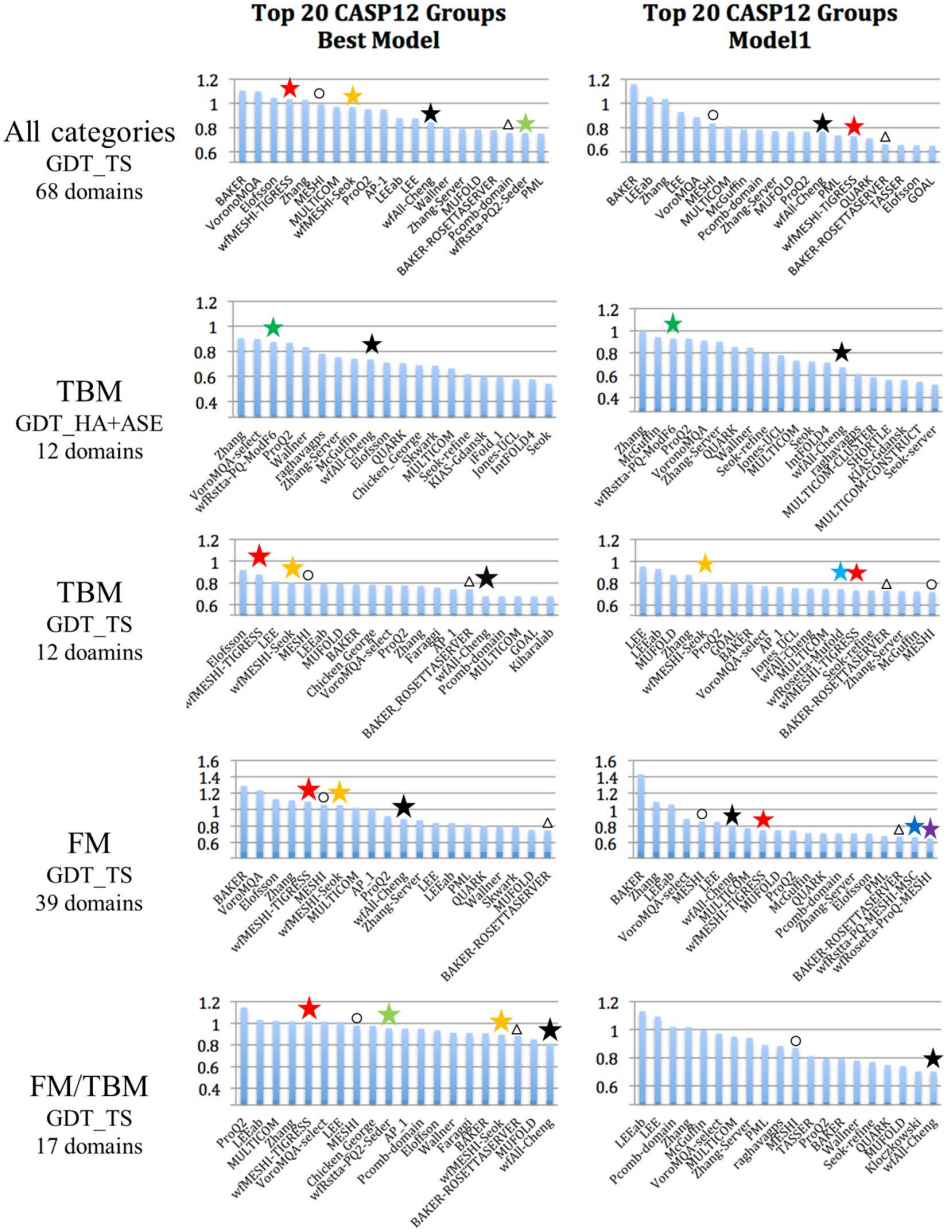
Average z-scores (>−2.0) of the 20 top CASP12 groups, WeFold pipelines are marked with asterisks (Black = wfAll-Cheng; Red = wfMESHI-TIGRESS; Orange = wfMESHI-Seok; Light green = wfRstta-PQ2-seder; Dark green = wfRstta-PQ-ModF6; Light blue = wfRosetta-MUFOLD; Dark blue = wfRstta-PQ-MESHI-MSC; Purple = wfRosetta-PQ-MESHI). The results of MESHI and BAKER-ROSETTASERVER are marked by black circle and triangle respectively. Only those groups that submitted models for at least half of the targets are considered. Chart on the left shows top 20 groups/servers when considering the best model submitted by each group for each target. Chart on the right shows top 20 groups/servers when considering Model 1 only. CASP assessors used GDT_HA + ASE only for TBM targets hence the double depicting of that category. Source: http://www.predictioncenter.org/casp12/zscores_final.cgi.

An aggregated summary of WeFold2 and WeFold3 results is presented in Fig. 3, which depicts the best per-target decoy submitted to CASP by all groups (blue) and the best submitted by WeFold (red). In both events WeFold pipelines submitted some of the best decoys (marked by red asterisks) as well as many other high quality ones. The figure also suggests an improved performance in WeFold3 compared with WeFold2, with a larger proportion of best or very-close-to-best decoys. The insert histograms in Fig. 3 depict the distributions of quality differences (Δ) between the best CASP decoys and their corresponding best WeFold decoy. As it can be seen in the inserts twice as many WeFold3 best models (40%) were also the best CASP decoy (Δ equal to zero) than were the WeFold2 models (20%) and most models in WeFold3 were close to the best CASP decoys (Δ close to zero). The CASP assessment considers each pipeline separately and yet shows a similar trend. None of the WeFold pipelines ranked high in CASP11, but eight of them did in CASP12 (Fig. 4).

Below we present detailed comparisons of the WeFold2 and WeFold3 pipelines.

### Detailed pipeline comparison

**Figure 5:**
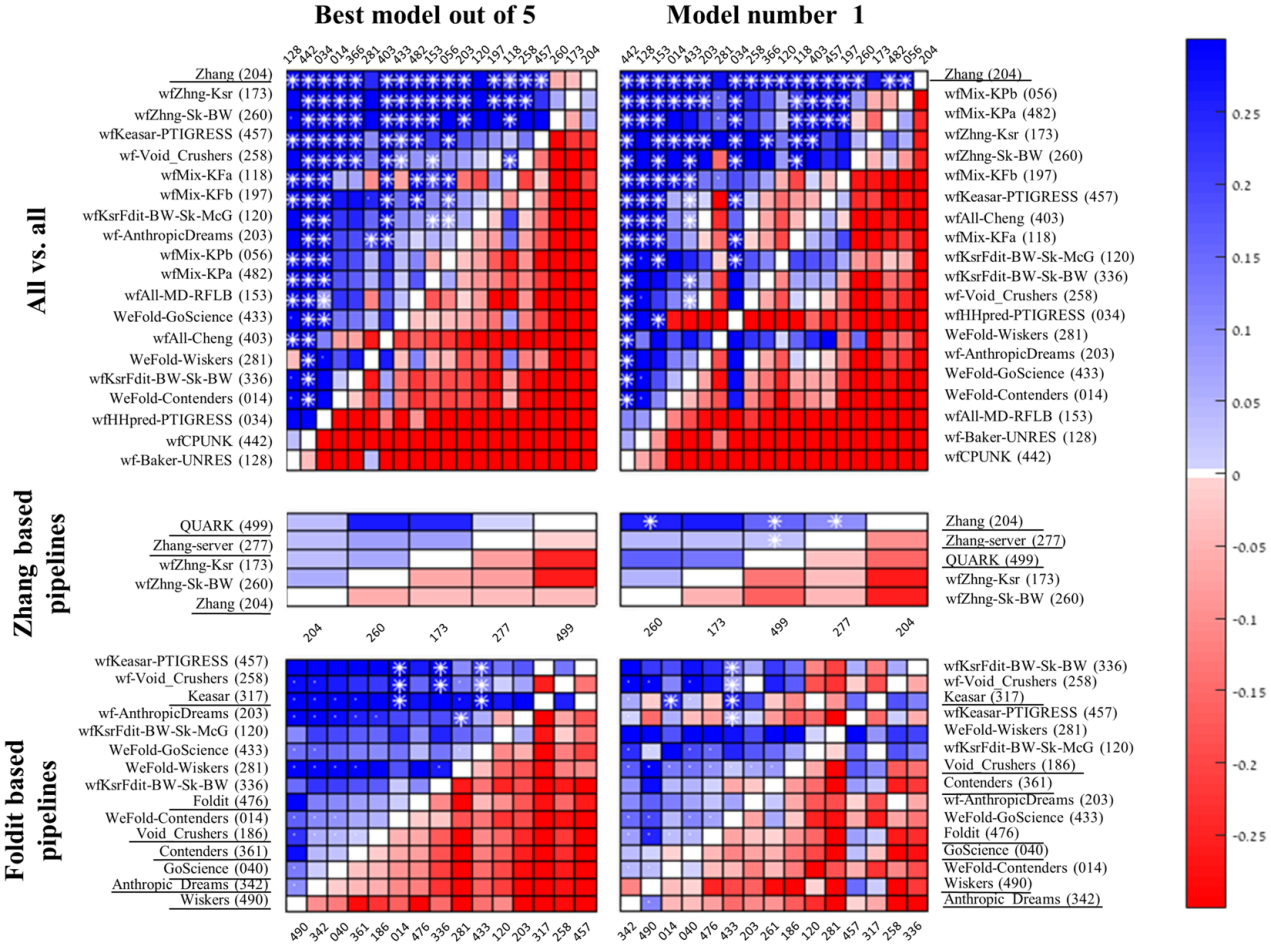
Pairwise comparison of WeFold and related (underlined) CASP11 groups. Each cell represents a comparison between the row and column groups, based on the subset of targets they both predicted. Cell colors depict the difference in average z-scores (GDT_TS). Blue indicate better performance of the row group. Asterisks indicate statistical significance (p < 0.05; Wilcoxon two-sided pair test). Dots indicate that the two groups shared no more than ten targets. Rows are ordered by decreasing number of significant cells, and then by blue cells. Source: http://www.predictioncenter.org/casp12/zscores_final.cgi.

To allow comparison among WeFold pipelines and between WeFold pipelines and CASP groups chosen as gold standards, we compared each pair of pipelines based on the intersection of their submitted targets. For this subset of targets we compared the mean GDT_TS z-score value of the best submitted decoy and the first submitted one (model 1). The statistical significance of results was estimated using two-sided Wilcoxon paired test (as implemented in MATLAB^35^). In Fig. 5 a blue cell indicates that the row pipeline outperforms the column pipeline, white asterisks indicate statistical significance (p < 0.05), and a white dot indicates that the two groups have no more than ten targets in common.

With almost each pipeline submitting a unique subset of the targets, rigorous comparison is only possible between pairs of pipelines (see legends for details). Yet, an *ad*-*hoc* ordering is implied by associating each row-pipeline with the number of column pipelines that it outperforms, with higher weight on significant differences.

The CASP tradition is to rank prediction groups by either their model 1 or the best-out-of-five. The former is used with easy, template based modeling targets, and the latter with hard, free modeling, targets. Yet splitting WeFold predictions by category would render many submission sets of pipelines too small to analyze. Thus, here we focus on best-out-of-five (left-hand matrices in Fig. 5) but also provide the first model results for comparison. Generally speaking the first model results seem less stable with fewer significant differences between the groups (162 versus 186 in WeFold2 and 114 versus 88 in WeFold3).

#### WeFold2

Our first step in analyzing the results of the tertiary structure prediction pipelines is to compare their performances in a coherent way. Figure 5, top panel, presents an all-against-all comparison of pipeline performances in WeFold2. The leading Zhang group was added to this analysis for calibration, and as a gold standard. As expected it outperformed most of the pipelines, yet it does not outperform the two top WeFold2 pipelines *wfZhng*-*Ksr* and *wfZhng*-*Sk*-*BW* (groups 173 and 260).

Figure 5, mid panel, depicts these two Zhang-based WeFold pipelines (groups 173 and 260) along with three Zhang groups (underlined). Zhang provided WeFold with decoy sets of single domain targets that were generated by his own servers (groups 499 and 277). Each of the servers selected from its own decoy set and each of the WeFold pipelines selected and submitted five decoys from the combined sets (with refinement in the case of group 260). The Zhang human group (204) incorporated this decoy set (and other sources) into its I-TASSER algorithm. None of the pairwise comparisons is statistically significant when considering the best of five models submitted by the Zhang-based pipelines. Yet, although the Zhang servers performed best, the WeFold pipelines integrated them better than Zhang’s human group.

**Figure 6:**
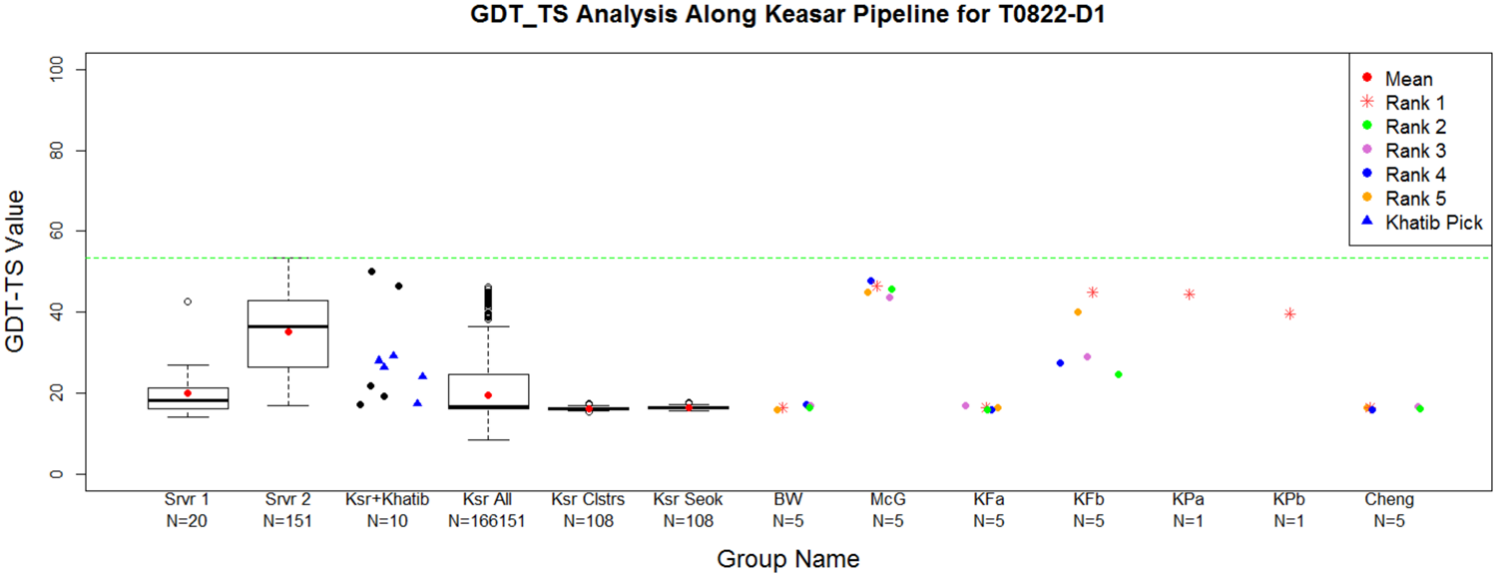
Box and whiskers plots represent the steps in Keasar-Foldit-based pipelines for target T0822-D1. First column represents the 20 models created by the servers at stage 1. Second column represents the 151 server models that are made available by the CASP organizers (stage 2). Keasar selects a subset of 10 server models using MESHI. These models are marked as dots in the third column. Then Khatib selects 5 of those models (marked with triangles). Khatib’s selected models (starting points) are given to the Foldit players. The Foldit players created a wide range of models, some of which were substantially better than the starting points as shown in column 4. However, column 5 shows that the clustering and filtering algorithm did not select those best models. Column 6 shows the clusters after refinement by Seok’s lab. Columns 7–13 represent the final selection by different WeFold groups, which selected either exclusively from the clusters in column 6, or from a combination of these and Zhang’s clusters, or from a combination of all the models shared by various WeFold groups and servers. Green line is the best model submitted to CASP11 for that target considering all the CASP11 groups. Note that the tick labels along the x-axis also show the number of models in each step of the pipeline. Box and whiskers plots for all the other targets attempted by the Keasar-Foldit pipelines and Zhang pipelines are in the Supplementary Materials.

The ranking of CASP11 servers by the non-Wefold Keasar group (317) served as a starting point to sampling and refinement by the seven Foldit-based pipelines (Fig. 5, bottom panel). The small numbers of targets submitted by most of them (white dots) reduce the statistical reliability of any specific pairwise comparison. Yet, two trends are apparent. First, all the WeFold-Foldit pipelines performed better than their non-WeFold counterparts (the same people, sampling with different starting points). That is, the starting points provided to Foldit players by a MESHI selection among server models were overall better than those selected by Rosetta among those generated by the Rosetta server. Another observation is more intriguing. Two of the individual WeFold-Foldit groups (258 & 203) performed better than more sophisticated pipelines that used pooled decoys from all groups. Specifically, the decoys selected (manually) by the individual groups were within the pool and some of them were missed. Table 3 offers a plausible explanation. It follows the gradual loss of the best decoys along the filtering and clustering steps of the pipelines. These pipelines started with hundreds of thousands of decoys generated by the Foldit players and then reduced them to a hundred decoys by a filtering and clustering process, in order to make the refinement and selection steps more manageable given the time constraints. Table 3 shows that for a majority of the T0XXX targets (12/19) the overall GDT_TS loss is less than 4 GDT_TS percentage units for the complete pipeline. However, the GDT_TS loss in the filtering and clustering steps were significant for some targets as illustrated in Fig. 6, which shows box and whiskers plots representing the steps in Keasar-Foldit-based pipelines for target T0822-D1. The first and second columns represents the models created by the servers at stage1 and 2, respectively. Keasar selected a subset of 10 server models using MESHI. These models are marked as dots in the third column. Then Khatib selected 5 of those models, which are marked with triangles. Khatib’s selected models are given to the Foldit players who created a wide range of models, some of which were substantially better than those provided to them by Khatib as shown in column 4. However, column 5 shows that the clustering and filtering algorithm did not select those best models. In fact, Table 3 shows that the best models were filtered out because they had both high Rosetta energies and were over 20Å from the model with lowest Rosetta energy. The Supplementary Materials include box and whiskers plots that show the gradual loss of the GDT_TS values at each of the steps of the prediction pipelines for all the targets attempted by the WeFold teams.

**Table 3:** The GDT_TS loss for the different steps in the complete clustering process for T0XXX targets, as measured by comparing the GDT_TS difference between the best GDT_TS before and after the different stages; *energy* is loss after applying the Rosetta energy filter cutoff, *rmsd1* is the loss after applying the filter that excluded models too different from the lowest Rosetta energy model, *energy* + *rmsd1* is the cumulative loss by applying both energy and rmsd1 filters, *clustering* is the loss after clustering, and Total loss refers to the complete cumulative loss after both filtering and clustering.

Stages combo stages	Energy	rmsd1	Energy + rmsd1	Clustering	Total loss
T0759	−1.0	0.0	−1.0	−1.8	−2.9
T0763	−1.9	−7.9	−8.2	−0.1	−8.3
T0765	0.0	−3.9	−3.9	0.0	−3.9
T0769	−2.1	0.0	−2.1	−1.0	−3.1
T0773	−2.2	0.0	−2.2	−0.7	−3.0
T0785	−2.5	0.0	−2.5	−0.5	−3.0
T0787	−1.3	−1.5	−2.5	−0.5	−3.0
T0797	−0.1	0.0	−0.1	−0.1	−0.2
T0803	−0.2	−17.0	−17.0	−0.7	−17.7
T0816	−8.8	−8.8	−8.8	−17.6	−26.5
T0818	−2.4	0.0	−2.4	−1.5	−3.9
T0820	−1.7	−2.1	−2.6	−1.1	−3.8
T0822	−16.4	−25.0	−28.5	−0.2	−28.7
T0824	−3.9	−3.0	−4.4	−2.1	−6.5
T0837	−8.3	−5.0	−8.5	−0.4	−8.9
T0838	−0.8	0.0	−0.8	−0.4	−1.2
T0848	0.0	0.0	0.0	−1.8	−1.8
T0853	−1.6	−5.8	−7.4	−0.5	−7.9
T0855	−1.3	−1.3	−1.3	−1.7	−2.9
**Median**	**−1**.**7**	**−1**.**5**	**−2**.**5**	**−0**.**7**	**−3**.**8**

#### The Refinement pipelines in CASP11

**Figure 7:**
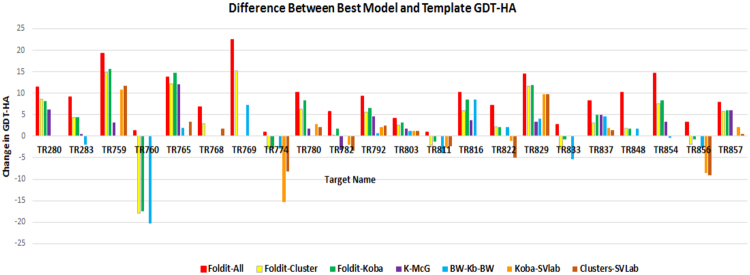
Comparison of GDT_HA differences between top model in each step of the refinement pipeline and the original model provided by the CASP11 organizers for each target. The steps are identified by color bars representing the difference between the GDT_HA of the starting model and the GDT_HA of (1) the best model among those generated by Foldit players (Foldit-All), (2) the best model among the clusters (Foldit-Cluster), (3) the best model among the clusters refined by KoBaMIN (Foldit-Koba), (4) the best selection by McGuffin (K-McG), (5) the best selection by Wallner/ProQ2 (BW-Kb-BW), (6) the best selection by SVLab of KoBaMIN-refined clusters (Koba-SVlab), and (7) the best selection by SVLab based on unrefined clusters (Clusters-SVLab).

The results by the WeFold pipelines in CASP11 (there were no refinement WeFold pipelines in CASP12) show that Foldit players generally improved the starting model. Figure 7 shows the improvement or deterioration measured by the best GDT_HA for each step of the pipelines and for each target. GDT_HA is the High Accuracy GDT defined as *GDT_HA* = (*GDT_P0*.*5* + *GDT_P1* + *GDT_P2* + *GDT_P4*)/*4*, where *GDT_Pn* denotes percent of residues under distance cutoff <= *n*Å. This figure shows that in the first step of the pipelines (marked as Foldit-All), Foldit players were able to improve the starting model in 22/22 targets attempted, i.e. they generated models that were better than the starting one. For the second step (marked as Foldit-Clusters in Fig. 7), 17/22 targets show an improvement after clustering by Wallner. In the third step (marked as Foldit-Koba), the bars show improvement for 15/20 targets after refinement by KobaMIN. Finally, bars marked as *wfFdit*-*K*-*McG*, *wfFdit*-*BW*-*KB*-*BW*, *wfFdit_BW_K_SVGroup*, *and wfFdit_BW_SVGroup* show the best GDT_HA among the 5 models submitted by those pipelines. *wfFdit*-*K*-*McG* selected an improved model for 12/17 targets, *wfFdit*-*BW*-*KB*-*BW* selected an improved model for 10/17 targets, *wfFdit_BW_K_SVGroup* selected an improved model for 7/12 targets, and *wfFdit_BW_SVGroup* selected an improved model for 9/14 targets. In 17/20 cases, KobaMIN improved the top model of the Foldit-Cluster group.

A similar analysis was conducted for the WeFold pipelines in CASP10, where the highest improvement per target was 10.51^7^. Figure 7 shows that 7 of the 22 CASP11 refinement targets were improved by 10 or more GDT_HA points. Among the best cases, TR769 and TR759 show improvements with respect to the initial model of around 20 points.

**Figure 8:**
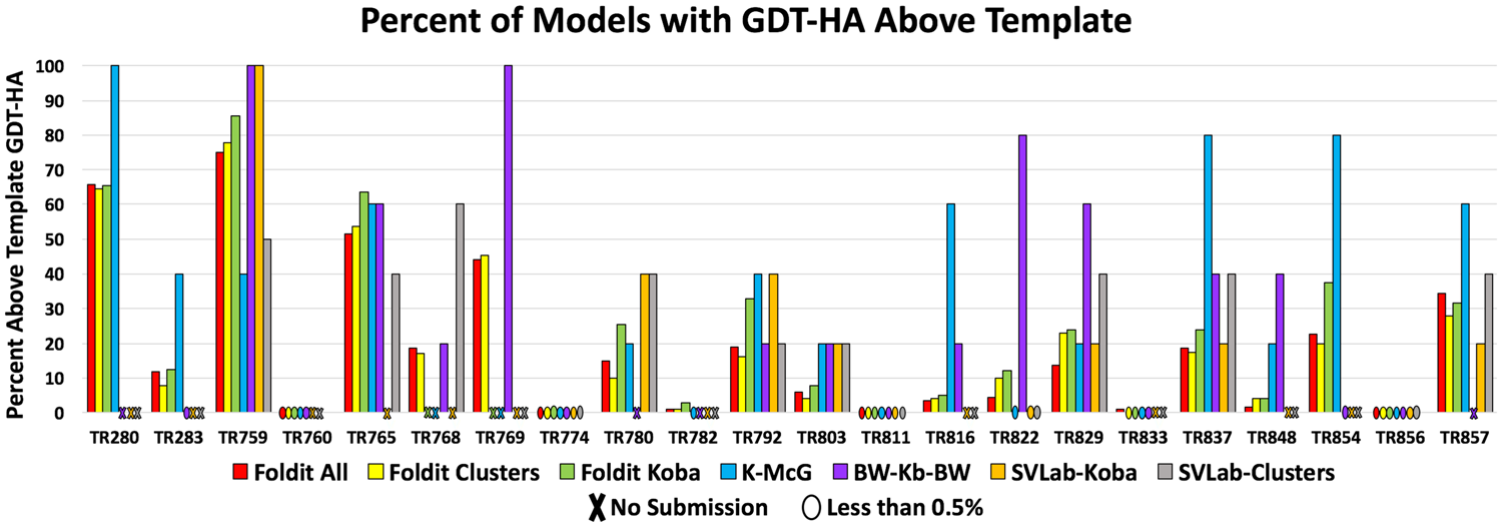
Chart comparing the percentage of models in each step of the refinement pipeline that improved the GDT_HA of the original model provided by CASP organizers. The steps are identified as follows: (1) models generated by Foldit players (Foldit-All), (2) clusters (Foldit Clusters), (3) clusters refined by KoBaMIN (Foldit Koba), (4) selection by McGuffin (K-McG), (5) selection by Wallner/ProQ2 (BW-Kb-BW), (6) selection by SVLab of KoBaMIN-refined clusters (SVLab-Koba), and (7) selection by SVLab based on unrefined clusters (SVLab-Clusters).

Figure 8 shows a comparison of the percentage of models in each step that have a higher GDT_HA than the starting model. The bars represent steps just like in Fig. 7 but in this case they show the percentage of models with GDT_HA higher than the template in each step of the pipeline to provide an idea of where the good models generated by Foldit are lost in the pipelines. Ideally, we would need to see an increased percentage of good models, i.e. enrichment, as the size of the sets is reduced from hundreds of thousands (generated by Foldit) to hundreds (clusters) to five, and the best models are kept in those sets. Unfortunately, this is not always the case as can be seen in Fig. 8.

We investigate the effect of the filtering and clustering steps in the refinement pipelines (the Foldit Clusters step in Figs 7 and 8) by comparing the maximum GDT_TS scores after each of the different steps in the clustering pipeline, i.e., by calculating the GDT_TS loss before and after the different steps in the clustering/filtering pipeline, (see Table ST1 in Supplementary Materials). For the refinement targets the overall GDT_TS loss is similar to the T0XXX targets (see Table 3), a majority (14/22) of the targets have GDT_TS loss less than five GDT_TS units. The difference is that most of the filtering is done based on the Rosetta energy and virtually none on the distance to the lowest energy model, because the structural ensemble in refinement is tighter. The only case where the clustering really fails is for target TR760. The best GDT_TS in the initial ensemble is 59.8. Almost 20 (19.8) GDT_TS units are lost after clustering and the best GDT_TS is 39.8. The reason for this failure is that the models with best GDT_TS have very unfavorable Rosetta energy (>200 Rosetta Units), and were filtered out by the energy based filter.

Nevertheless, it can be seen that the WeFold pipelines submitted models that were substantial improvements over the template for the majority of the refinement targets suggesting that the Foldit-based pipelines should be continued for this category.

#### WeFold3

**Figure 9:**
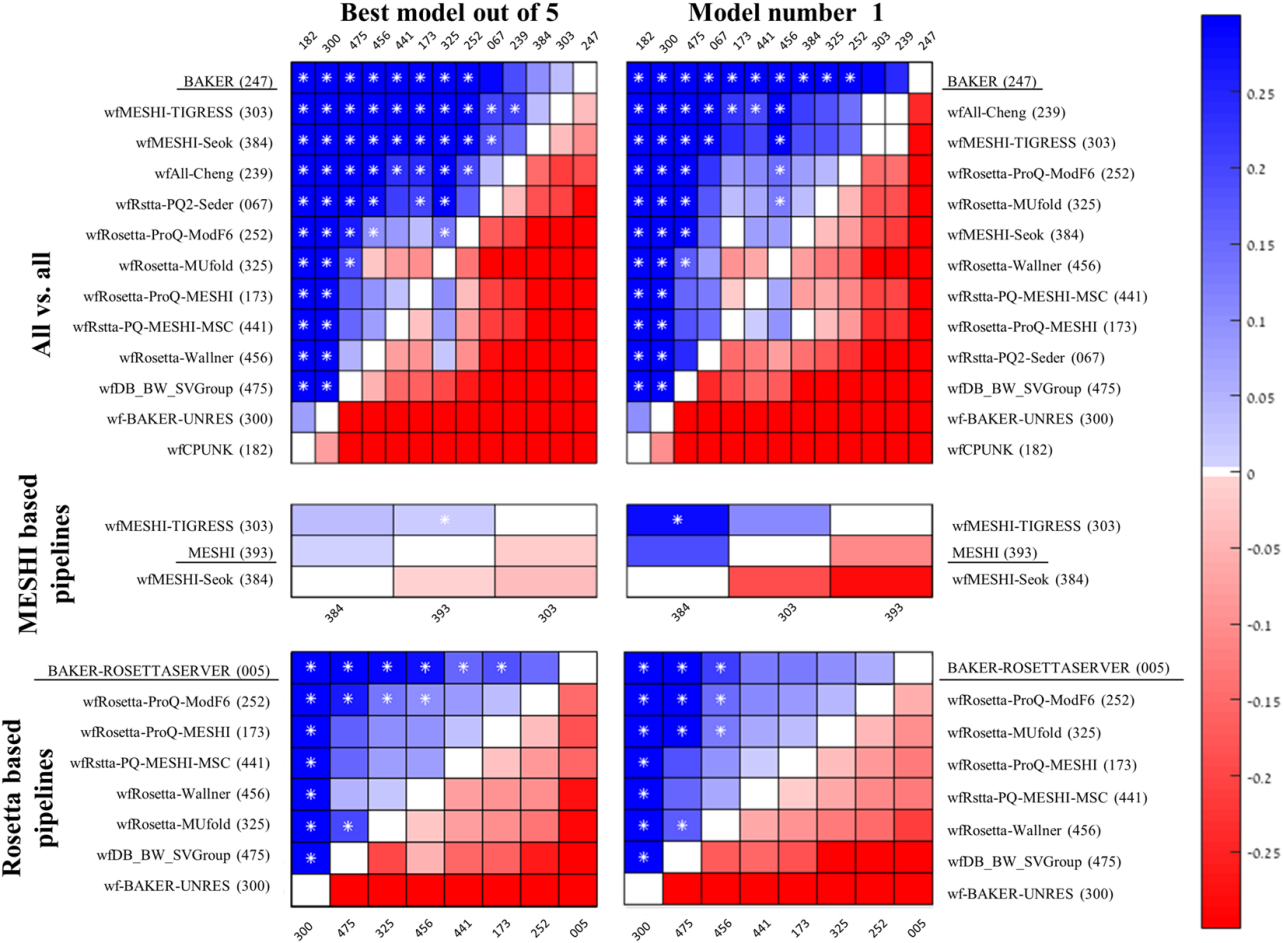
Pairwise comparison of WeFold and related (underlined) CASP12 groups. Each cell represents a comparison between the row and column groups, based on the subset of targets they both predicted. Cell colors depict the difference in average z-scores (GDT_TS). Blue indicate better performance of the row group. Asterisks indicate statistical significance (p < 0.05; Wilcoxon two-sided pair test). Rows are ordered by decreasing number of significant cells, and then by blue cells. Source: http://www.predictioncenter.org/casp12/zscores_final.cgi.

The top panel of Fig. 9 presents an all-against-all comparison of pipeline performances in WeFold3. Again, a leading CASP group (BAKER) is added to this analysis as a gold standard. It outperforms all the pipelines, though its advantage over the three top pipelines is not statistically significant. The top four pipelines are also consistent with the CASP12 ranking (Fig. 4 top panel).

Figure 9, mid panel depicts two top WeFold pipelines (groups 303 and 384) as well as one non-WeFold group (MESHI). These three groups started from the same start point, which is the set of CASP server decoys ranked by MESHI-score, and tried to refine them. The refinement by Princeton_TIGRESS outperforms the other two resulting in the strongest WeFold3 pipeline.

Figure 9, bottom panel compares the seven Rosetta-based WeFold pipelines as well as the non-WeFold group BAKER-ROSETTASERVER. All these groups selected domain decoys from the same pool, hundreds of thousands decoys generated by Rosetta (Fig. 2). The Rosetta domain parsing method tries to identify template structures for optimal sequence similarity and structural coverage. If a confident PDB template cannot be identified, it predicts boundaries from a multiple sequence alignment based on start and end points of sequence alignment clusters. The large Rosetta data sets were reduced by filtering with ProQ2. Thus, all the Rosetta-based pipelines used the same reduced datasets as starting point. Yet, their performances differed considerably from that of the BAKER-ROSETTASERVER. A post CASP analysis suggests that domain assembly has been an obstacle for two of them (*Rstta*-*PQ*-*MESHI* and *Rstta*-*PQ*-*MESHI*-*MSC*), who submitted independent segments for each presumed domain. Often the official CASP evaluation units (domains) were different reducing the performance measure. However, this was not the only obstacle. Another post CASP analysis using only single domain proteins shows that ProQ2 performed well in most cases when selecting one thousand models among the hundreds of thousands Rosetta-generated models but the QA methods missed those best models in most cases.

The comparison among Rosetta-based pipelines shows that some top-performing QA methods like MESHI, which are trained and tested on server models do not perform equally well when applied to Rosetta server models pointing to the need for more data to generate more general scoring functions. The bottom panel of Fig. 9 also shows that *Rstta*-*PQ*-*MESHI* and *Rstta*-*PQ*-*MESHI*-*MSC* which only differ in the machine learning method used to combined the same features and applied to the same decoys, had similar performance thus confirming the results shown in^10^, which state that different machine learning methods do not seem to make a substantial difference in the performance of the scoring functions.

**Figure 10:**
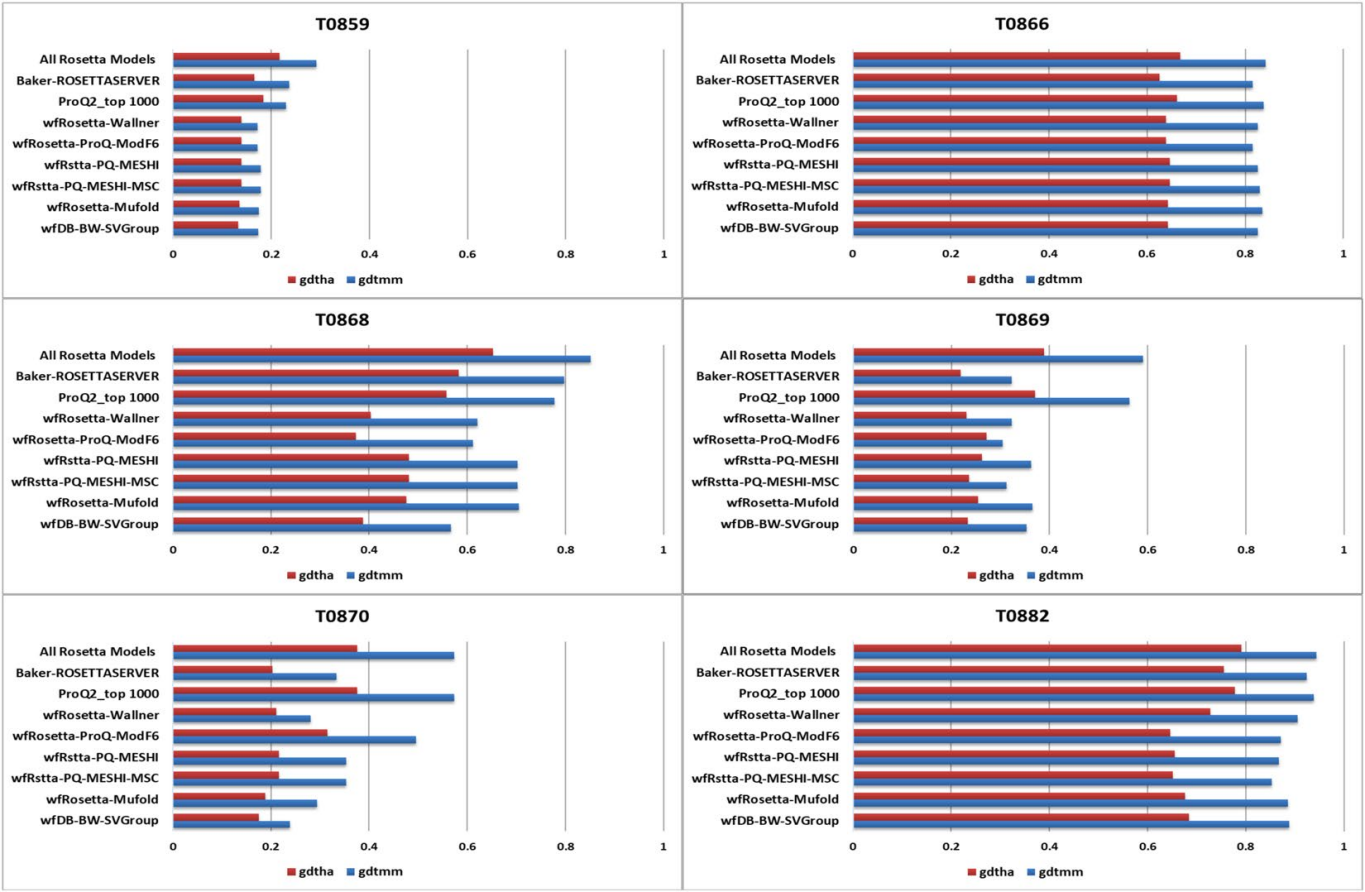
Bar plots show the down-selection process across the Rosetta-based pipelines for 6 targets using GDT_HA and GDT_MM. In each row, red bars represent best GDT_HA and blue bars represent best GDT_MM. GDT_MM is a Baker-lab specific metric, where the MAMMOTH alignment algorithm (MM = MAMMOTH) is used for the superposition (slight variations with respect to GDT_TS are based on alignment). Top row shows best GDT_HA (or MM) among the hundreds of thousands of models generated by Rosetta for that target. Next row shows the best GDT_HA (MM) among the best 5 selected by the BAKER-ROSETTASERVER; next row shows the best GDT_HA (MM) among the one thousand models selected by ProQ2; the remainder rows show the best GDT_HA (MM) among the best 5 selected by the Rosetta-based WeFold groups (one set of bars each).

Figure 10 shows the best models at each step of the pipelines measured by GDT_HA and GDT_MM for each step of the Rosetta-based pipelines and for six single domain targets (Fig. S3 in the Supplementary Materials show a similar plot for the remaining single domain targets). GDT_MM is a Baker-lab specific metric, where the MAMMOTH alignment algorithm is used for the superposition. It should match GDT_TS in all other respects. We used GDT_MM instead of GDT_TS because Rosetta enables GDT_MM direct calculation using its silent files, thus avoiding the extraction of millions of PDB files. Silent files are Rosetta-specific file formats used for efficient concatenated storage of large numbers of structures. In total, 32,474,636 decoys were generated by BAKER-ROSETTASERVER and scored using ProQ2 during CASP12. As it can be seen in Fig. 10, the best models are not selected at each step of the pipelines in a consistent manner. This figure also shows that ProQ2 was a significant improvement compared to the filtering and clustering methods used for CASP11.

### Top performing groups in WeFold3 for all categories

Figure 4 (top panel) shows that four WeFold3 groups ranked among the top 20 of the CASP12 groups/servers. These charts show the top 20 CASP12 groups/servers according to average GDT_TS z-scores > −2 when considering all 3 categories, TBM, TBM/FM, and FM and only those groups that submitted models for at least half of the targets. The chart on the left-hand side shows the top 20 groups/servers when considering the best model submitted by each group for each target and the chart on the right-hand side shows top 20 groups/servers when considering Model 1 only.

The two pipelines that are based on MESHI selection, *wfMESHI*-*TIGRESS* and *wfMESHI*-*Seok*, benefited from the top performance of the MESHI group and one of them, *wfMESHI*-*TIGRESS* slightly outperformed MESHI when considering the best model submitted by each group. Group *wfAll*-*Cheng*, which used all the models shared by all the WeFold3 groups but usually selected models from the MESHI-based groups (as shown in Fig. S1 in the Supplementary Materials) ranked 13th in both cases, when considering the best model and model 1 only, showing a significant improvement with respect to its own performance in CASP11 when it ranked 47th. Of the Rosetta-based teams, none ranked among the top 20 when considering the best model submitted. Finally, group *wfRstta*-*PQ2*-*Seder*, which uses a mix of Rosetta and server models, also ranked among the top 20. In the next sections, we analyze the performance of the WeFold3 pipelines in the 3 subcategories TBM, TBM/FM, and FM.

#### Top performing groups in WeFold3 for the TBM category

Here we explore the performance of the WeFold3 pipelines in the TBM (Template-Based Modeling) category. Proteins in this category are those for which a close relationship could be detected by sequence similarity searches providing one or more templates. Figure 4, second and third panels, show the top 20 ranking CASP12 groups/servers when considering the average z-scores of both the assessors’ formula and GDT_TS, respectively. The CASP12 assessors used *GDT_HA* + *ASE* (Accuracy Self Estimate) for the assessment of models in this category. ASE is defined as

$$ASE=100.0\ast (1-Mean(|S(t{f}_{i}/{d}_{0})-S({d}_{i}/{d}_{0})|))$$

where *tf*_*i*_ is temperature factor of the *i*-*th* residue in the model and *d*_*i*_ is distance between *i*-*th* residues in lga alignment (sequence dependent mode)

$$S(x)=1/(1+{x}^{2})$$

*d*_*0*_ is the scaling factor, set *d*_*0*_ = *5*.*0*

(http://www.predictioncenter.org/casp12/doc/help.html#ASE).

These charts show that focusing on either GDT_TS or ASE produced different results. In fact, when considering the assessors’ formula, two WeFold pipelines ranked among the top 20: *wfRosetta*-*ProQ*-*ModF6* and *wfAll*-*Cheng*. Notice that *wfRosetta*-*ProQ*-*ModF6* selected best 5 models among the models generated by the BAKER-ROSETTASERVER and neither the BAKER-ROSETTASERVER nor the BAKER group are among the top 20 in this category. The high performance of the *wfRosetta*-*ProQ*-*ModF6* group was mainly due to accurate ranking and accuracy self-assessment (ASE) using the ModFOLD6_rank method^33^. On the other hand, when using GDT_TS values, the two MESHI-based groups and *wfAll*-*Cheng* ranked among the top 20 when considering both the best model among the 5 submitted and model 1. *wfMESHI*-*Seok* showed better results in TBM category than in other categories probably because the refinement method was originally trained to improve template-based models.

#### Top performing groups in WeFold3 for FM category

In this section, we analyze the performance of the WeFold3 pipelines in the FM (Free Modeling) category. Proteins in this category are those for which no structural template could be found by structural similarity searches. Figure 4, fourth panel, shows the top 20 ranking CASP12 groups/servers when considering the average z-scores of the GDT_TS values. According to these charts, three WeFold pipelines ranked among the top 20 when considering the best model submitted: *wfMESHI*-*TIGRESS*, *wfMESHI*-*Seok*, and *wfAll*-*Cheng*. Note that none of the pipelines that started with Rosetta decoys are among the top 20 in this case. On the other hand, two pipelines made it to the top 20 when considering models 1 only: *wfRstta*-*PQ*-*MESHI*-*MSC* and *wfRosetta*-*ProQ*-*MESHI*, which started with the 1000 models filtered with ProQ2 and selected the best 5 by combining the same features in Keasar’s dataset using different machine learning techniques^10^. However, these pipelines did not outperform the BAKER-ROSETTASERVER. The low performance of the Rosetta-based pipelines in this category can mainly be attributed to incorrect domain and difficult predictions. Compared to the pipelines that used “all-server models”, the Rosetta-based pipelines performed worse. Not surprisingly since they are based on a single server’s models, i.e. BAKER-ROSETTASERVER.

#### Top performing groups in WeFold3 for TBM/FM category

In this section, we analyze the performance of the WeFold3 pipelines in the TBM/FM category. Figure 4, bottom panel, shows the top 20 ranking CASP12 groups/servers when considering the average GDT_TS z-scores. According to these charts, four WeFold3 pipelines ranked among the top 20: *wfMESHI*-*TIGRESS*, *wfRstta*-*PQ2*-*Seder*, *wfMESHI*-*Seok*, and *wfAll*-*Cheng*. Like in the FM category, *wfMESHI*-*TIGRESS* performed slightly better than MESHI when considering the best model submitted. Note that none of the pipelines that started exclusively with Rosetta decoys did better than BAKER-ROSETTASERVER, which ranked 18th. We believe that the performance of the BAKER-ROSETTASERVER-based pipelines could be improved by including a new component to the pipelines to take care of domain splitting. Note that the *wfAll*-*Cheng* pipeline, which selected many models from the *wfMESHI*-*Seok* and *wfMESHI*-*TIGRESS* pipelines, ranked 20th when considering model 1 only even though the MESHI-based pipelines are not in the top20 list, which highlights the ability of this meta-pipeline to select top-ranking models.

## Conclusions

This paper discusses the second and third round of the WeFold experiment, WeFold2 and WeFold3, which took place in the context of CASP11 and CASP12, respectively. Twenty-one groups participated in WeFold2 and contributed a wide range of methods, some already proven successful and some in experimental stage, creating a unique opportunity for the generation of 23 pipelines. Sixteen groups participated in WeFold3, creating 12 pipelines. The scale and diversity of the methods tried in WeFold could not have been achieved by any individual lab or even by any collaboration among a few partners. The number and diversity of the models amassed by the WeFold project cannot be found anywhere else. Even more importantly, WeFold has created a strong sense of community among its participants, with well-defined goals and purposes.

By analyzing WeFold2 and WeFold3 as two successive case studies, not only can we see that the first helped to shape the more successful second one, but also provide guidelines for future efforts. The scale of the WeFold collaboration and the richness of the gathered results highlight a new challenge: as we see new ways to improve the sampling (either by gathering models from different methods or by including citizen scientists), domain splitting, decoys set reduction, assessment and selection steps become bottlenecks that limit the success of the pipelines. Faced with the large scale and wide range of models, many of which are of mediocre quality, the clustering/filtering algorithms struggle and the assessment and selection algorithms largely fail to consistently select the best models produced. Most QA methods are trained on TBM models and they do not perform well on mediocre ones. Although these problems have been affecting the CASP methods in general, they are significantly magnified in the WeFold pipelines. For example, most methods are trained on server models and fail to generalize on a wide range of models created by a single group. We have collectively taken action to deal with these bottlenecks and the performance of the WeFold3 pipelines improved substantially as a result.

A number of WeFold3 pipelines stood up: wfRstta-BW-ModF6 outperformed ROSETTASERVER as well as the other WeFold pipelines in the TBM category. wfMESHI-TIGRESS performed slightly better than its non-WeFold counterpart MESHI, especially when considering model 1 and wfAll-Cheng performed consistently well in all categories. Efforts are underway to provide the codes for these pipelines to the public using GitHub or Jupyter notebooks.

An important goal of the project is to create an inclusive community that reaches out beyond CASP and brings into the field people, methods, disciplines, and technologies that can contribute to the solution of such a complex problem. This effort has produced results^10^ which show that the performance of the methods depends on the metric used and that certain features, such as GOAP^36^, have more significance than the method used, while others only add noise to the scoring function. Further efforts in improving QA are under development and the resulting methods will be tested in CASP13^11^.

One of the main problems of the WeFold experiment, which still needs to be addressed, is that the full pipelines are assembled on the first day of the CASP event and no prior benchmarking or testing is done, other than for the individual components. In some cases, this may result in suboptimal pipelines that cannot achieve peak performance and cannot compete with the individual group methods, which may have been heavily benchmarked before CASP. Nevertheless, despite these challenges, this paper shows that some of the tertiary structure prediction pipelines have ranked among the top performing groups in the CASP12 experiment.

The scale of the data garnered has also motivated us to leverage the power of ‘big data’ to our problems. We are working on significantly expanding the Keasar’s database^10^ to include a subset of the millions of models shared by our community. The vast number of models amassed, the collaboration among various labs, and the ability to attract outsiders with complementary expertise (e.g. machine learning) may give WeFold an edge to tackle the scoring and quality assessment problem. In fact, WeFold has great potential to bring protein structure prediction to the realm of data science and analytics.

## Electronic supplementary material

Supplementary Information
