# Supplementary material for: An analysis and evaluation of the WeFold collaborative for protein structure prediction and its pipelines in CASP11 and CASP12
Source: Sci Rep. 2018 Jul 2;8:9939. doi: 10.1038/s41598-018-26812-8 (PMC6028396; doi:10.1038/s41598-018-26812-8)
Supplement: Supplementary file 1 — Supplementary Information [file 41598_2018_26812_MOESM1_ESM.pdf]

# An analysis and evaluation of the WeFold collaborative for protein structure prediction and its pipelines in CASP11 and CASP12
## Supplementary Information

Chen Keasar[1], Liam J. McGuffin[2], Björn Wallner[3], Gaurav Chopra[4,5,6,7,8], Badri Adhikari[9], Debswapna Bhattacharya[9,10], Lauren Blake[11], Leandro Oliveira Bortot[12], Renzhi Cao[9], B.K. Dhanasekaran[13], Itzhel Dimas[11], Rodrigo Antonio Faccioli[14], Eshel Faraggi[15,16], Robert Ganzynkowicz[17], Sambit Ghosh[13], Soma Ghosh[13], Artur Giełdoń[17], Lukasz Golon[17], Yi He[18], Lim Heo[19], Jie Hou[9], Main Khan[20], Firas Khatib[20], George A. Khoury[21], Chris Kieslich[22], David E. Kim[23,24], Pawel Krupa[17], Gyu Rie Lee[19], Hongbo Li[9,25,26], Jilong Li[9], Agnieszka Lipska[17], Adam Liwo[17], Ali Hassan A. Maghrabi[2], Milot Mirdita[27], Shokoufeh Mirzaei[11,28], Magdalena A. Mozolewska[17], Melis Onel[29], Sergey Ovchinnikov[23,30], Anand Shah[20], Utkarsh Shah[29], Tomer Sidi[1], Adam K. Sieradzan[17], Magdalena Ślusarz[17], Rafal Ślusarz[17], James Smadbeck[21], Phanourios Tamamis[22,29], Nicholas Trieber[20], Tomasz Wirecki[17], Yanping Yin[31], Yang Zhang[32], Jaume Bacardit[33], Maciej Baranowski[34], Nicholas Chapman[35], Seth Cooper[36], Alexandre Defelicibus[14], Jeff Flatten[35], Brian Koepnick[23], Zoran Popović[35], Bartlomiej Zaborowski[17], David Baker[23,24,35], Jianlin Cheng[9], Cezary Czaplewski[17], Alexandre Cláudio Botazzo Delbem[14], Christodoulos Floudas[22], Andrzej Kloczkowski[17], Stanislaw Ołdziej[34], Michael Levitt[37], Harold Scheraga[31], Chaok Seok[19], Johannes Söding[27], Saraswathi Vishveshwara[13], Dong Xu[9,26], and Silvia N. Crivelli*[11,38]

Affiliations

[1] Department of Computer Science and Life Sciences, Ben Gurion University of the Negev, Israel

[2] Biomedical Sciences Division, School of Biological Sciences, University of Reading, Reading RG6 6AS, UK

[3] Division of Bioinformatics, Department of Physics, Chemistry, and Biology, Linköping University, Sweden

[4] Department of Chemistry, College of Science, Purdue University, West Lafayette, IN, USA

[5] Purdue Institute for Drug Discovery, Purdue University, West Lafayette, IN, USA

[6] Purdue Center for Cancer Research, Purdue University, West Lafayette, IN

[7] Purdue Institute for Inflammation, Immunology and Infectious Disease, Purdue University, West Lafayette, IN

[8] Purdue Institute for Integrative Neuroscience, Purdue University, West Lafayette, IN

[9] Department of Electrical Engineering and Computer Science, University of Missouri, USA

[10] Department of Computer Science and Software Engineering, Auburn University, AL, USA

[11] Lawrence Berkeley National Laboratory, Berkeley, CA, USA

[12] Laboratory of Biological Physics, Faculty of Pharmaceutical Sciences at Ribeirão Preto, University of São Paulo, Brazil

[13] Molecular Biophysics Unit and IISC Mathematics Initiative, Indian Institute of Science, India

[14] Institute of Mathematical and Computer Sciences, University of São Paulo, Brazil

[15] Research and Information Systems, LLC, Carmel, Indiana, USA and Department of Biochemistry and Molecular Biology, IU School of Medicine, Indianapolis, Indiana, USA

[16] Batelle Center for Mathematical Medicine, The Research Institute at Nationwide Children's Hospital, Columbus, OH, USA

[17] Faculty of Chemistry, University of Gdansk, Poland

[18] School of Engineering, University of California, Merced, CA, USA

[19] Department of Chemistry, Seoul National University, Seoul, Republic of Korea

[20] Department of Computer and Information Science, University of Massachusetts Dartmouth, USA

[21] Department of Chemical and Biological Engineering, Princeton University, USA

[22] Texas A&M Energy Institute, Texas A&M University, USA

[23] Department of Biochemistry, University of Washington, USA

[24] Howard Hughes Medical Institute, University of Washington, USA

[25] School of Computer Science and Information Technology, NorthEast Normal University, Changchun, China

[26] Christopher S. Bond Life Sciences Center, University of Missouri, USA

[27] Max Planck Institute for Biophysical Chemistry, Göttingen, Germany

[28] California State Polytechnic University, Pomona, CA, USA

[29] Artie McFerrin Department of Chemical Engineering, Texas A&M University, USA

[30] Institute for Protein Design, University of Washington, USA

[31] Baker Laboratory of Chemistry and Chemical Biology, Cornell University, Ithaca, NY, USA

[32] University of Michigan, USA

[33] Interdisciplinary Computing and Complex BioSystems (ICOS) research group, School of Computing, Newcastle University, Newcastle-upon-Tyne, UK

[34] Intercollegiate Faculty of Biotechnology, University of Gdańsk and Medical University of

Gdańsk, Poland

[35] Center for Game Science, Department of Computer Science & Engineering, University of Washington, USA

[36] College of Computer and Information Science, Northeastern University

[37] Department of Structural Biology, School of Medicine, Stanford University, CA, USA

[38] Department of Computer Science, University of California, Davis, CA, USA

10/28/2017

*Author to whom all correspondence should be addressed

Silvia N. Crivelli, Ph.D.

Computational Research Division

Lawrence Berkeley Laboratory, CA

1 Cyclotron Rd, Mail Stop 50F-1615

Berkeley, CA 94720

Emails: SNCrivelli@ucdavis.edu, SNCrivelli@lbl.gov

Phone: 925-367-5900

Running Title: Formative Assessment of WeFold

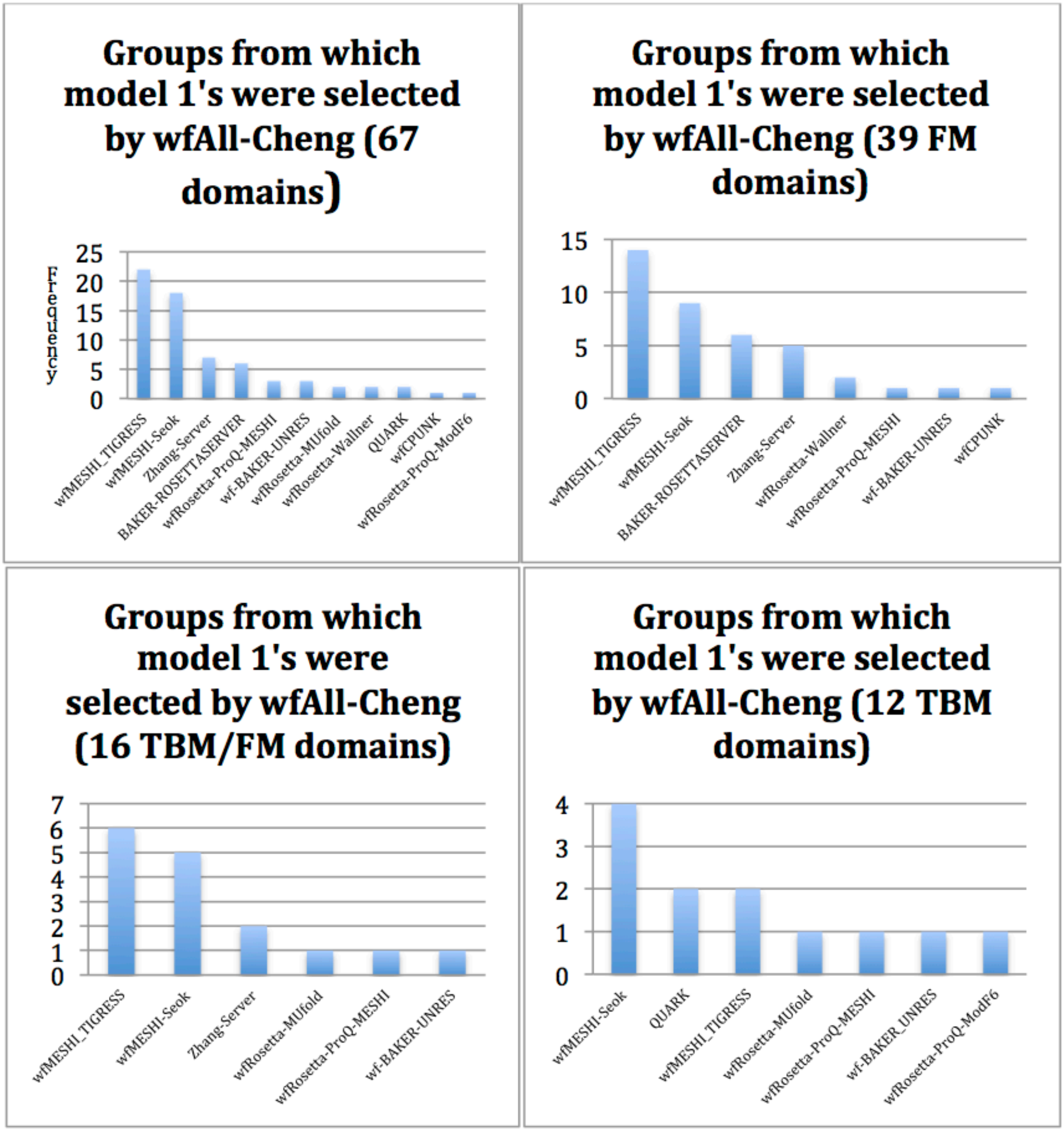


**Figure S1.** The wfAll-Cheng pipeline selected its model 1 among all models contributed by the WeFold pipelines as well as servers models. (Top left) Of the 67 CASP12 domains released so far, the wfAll-Cheng pipeline selected 22 models submitted by pipeline wfMESHI_TIGRESS and 18 models submitted by pipeline wfMESHI-Seok as model 1. (Top right) Of the 39 FM CASP12 domains released so far, the wfAll-Cheng pipeline selected 14 models submitted by pipeline wfMESHI_TIGRESS and 9 models submitted by pipeline wfMESHI-Seok as model 1. (Bottom left) Of the 16 TBM/FM CASP12 domains released so far, the wfAll-Cheng pipeline selected 6 models submitted by pipeline wfMESHI_TIGRESS and 5 models submitted by pipeline wfMESHI-Seok as model 1. (Bottom right) Of the 12 TBM CASP12 domains released so far, the wfAll-Cheng pipeline selected 4 models submitted by pipeline wfMESHI_Seok and 2 models submitted by pipeline wfMESHI-TIGRESS as model 1

**Table ST1:** The GDT_TS loss for the different steps in the complete clustering process for TRXXX targets, as measured by comparing the GDT_TS difference between the best GDT_TS before and after the different stages; *energy* is loss after applying the Rosetta energy filter cutoff, *rmsd1* is the loss after applying the filter that excluded models too different from the lowest Rosetta energy model, *energy+rmsd1* is the cumulative loss by applying both energy and rmsd1 filters, *clustering* is the loss after clustering, and Total loss refers to the complete cumulative loss by after both filtering and clustering.

| **stages** | **energy** | **rmsd1** | | **Clustering** | |
|---|---|---|---|---|---|
| **combo stages** | | | **energy+rmsd1** | | **Total loss** |
| TR280 | -1.1 | 0.0 | -1.1 | -1.8 | -2.9 |
| TR283 | -3.7 | 0.0 | -3.7 | -1.1 | -4.8 |
| TR759 | -2.8 | 0.0 | -2.8 | -1.6 | -4.4 |
| TR760 | -18.0 | -16.7 | -18.0 | -1.2 | -19.3 |
| TR765 | -1.0 | 0.0 | -1.0 | -0.7 | -1.7 |
| TR768 | -1.8 | 0.0 | -1.8 | -2.1 | -3.8 |
| TR769 | -3.4 | 0.0 | -3.4 | -3.9 | -7.2 |
| TR774 | -3.0 | 0.0 | -3.0 | -1.0 | -4.0 |
| TR780 | -2.9 | 0.0 | -2.9 | -1.1 | -4.0 |
| TR782 | -1.1 | 0.0 | -1.1 | -1.6 | -2.7 |
| TR792 | -0.6 | 0.0 | -0.6 | -3.1 | -3.8 |
| TR803 | -1.5 | 0.0 | -1.5 | 0.0 | -1.5 |
| TR811 | -1.7 | 0.0 | -1.7 | -1.4 | -3.1 |
| TR816 | -4.0 | 0.0 | -4.0 | -0.4 | -4.4 |
| TR822 | -2.9 | 0.0 | -2.9 | -2.2 | -5.0 |
| TR829 | -0.8 | 0.0 | -0.8 | -2.2 | -3.0 |
| TR833 | -3.9 | 0.0 | -3.9 | -1.9 | -5.8 |
| TR837 | -2.1 | 0.0 | -2.1 | -3.1 | -5.2 |
| TR848 | 0.0 | 0.0 | -6.7 | -1.6 | -8.3 |
| TR854 | -5.4 | 0.0 | -5.4 | -1.7 | -7.1 |
| TR856 | -3.1 | 0.0 | -3.1 | -2.1 | -5.2 |
| TR857 | 0.0 | 0.0 | 0.0 | -2.1 | -2.1 |
| **Median** | **-2.5** | **0.0** | **-2.8** | **-1.7** | **-4.2** |

## Description of the WeFold2 Pipelines

### *The Keasar-Foldit pipelines: wfKsrFdit-BW-Sk-BW and wfKsrFdit-BW-Sk-McG*

As their names suggest, the *wfKsrFdit-BW-Sk-BW* and *wfKsrFdit-BW-Sk-McG* pipelines shared most of their components, splitting only in their last step. They started from CASP server models, which were selected by Keasar. A subset of those models was provided to Foldit players, who sampled around and between them generating a massive set of decoys. This set was then shrunk to a manageable size of around one hundred by filtering and clustering performed by Wallner. After refinement of the cluster centers by Seok group, the two pipelines split. In one branch (*wfKsrFdit-BW-Sk-BW*) Wallner group selected the five submitted models from the set of refined cluster centers, in the other branch the selection was done by McGuffin group. Below we describe this process in detail.

*Sampling step*

The first step in these pipelines was the selection of 10-20 server models as starting points. The selection protocol, developed by the Keasar group is thoroughly discussed in [1]. Briefly, the protocol starts with a preprocessing step of energy minimization, which normalizes the server models by removing clashes and other distortions. Then, a large set of structural features is fed into an ensemble-learning predictor that was trained on a curated subset of CASP8, CASP9, and CASP10 server models. The list of top scoring models was uploaded to the WeFold site.

Up to five of those Keasar's top scoring models were provided as starting models in the Foldit game [2], allowing players to create hybrid predictions by combining different server models together in the same puzzle. Initially, Keasar’s top five ranked server predictions were selected, so long as they were not too similar (i.e. >2.5 Å RMSD) to one another, in order to provide Foldit players with a conformationally diverse set of starting structures. As CASP11 progressed, however, it became apparent that Foldit players were converging on server predictions that had been generated using the Rosetta energy function [3] (such predictions appear near-optimal in Foldit, which also uses the Rosetta energy function). For example, if players were given five of Keasar’s top-ranked server predictions for a particular CASP target, but one of those five was a RosettaServer model, the top-scoring Foldit solutions would all originate from the RosettaServer model and not from the other four starting structures. This was not exclusive to RosettaServer predictions, as this occurred with any server models that had been generated using the Rosetta energy function.

Since the in-game Foldit score is entirely based on Rosetta, from a player perspective this resulted in two different classes of server predictions: those that scored well in Foldit and those that scored poorly when initially loaded into the game. It is not surprising that players found it easier to improve Rosetta-based server predictions than those with very poor initial Foldit scores. Ideally, these two different classes of server models would have split into two separate Foldit puzzles for each CASP11 target: one with only Rosetta-based predictions and one without. This was not feasible with the time constraints of the CASP experiment, as only a handful of CASP11

targets were attempted in the first place, due to the throughput limitations of a typical personal computer running Foldit. Instead of posting puzzles for both classes of server models, one class was selected based on the majority of Keasar's top five ranked server predictions (e.g., if 3 out of Keasar's top 5 models were non-Rosetta models, only those 3 server predictions were given to the players and the 2 Rosetta-based models were ignored). Each CASP11 Foldit puzzle was typically accessible to players for 5-8 days, along with a sequence logo of secondary structure predictions generated by the SAM-T08 server [4]. Overall, Foldit players generated between 96K and 240K models per target (166K on average), which were uploaded onto the WeFold gateway.

*Clustering step*

By the time the numerous Keasar-Foldit models were available, the submission deadline was only one week away, too tight to allow computationally intensive assessment of their quality and refinement (discussed below). Therefore, in order to reduce the models ensemble to a manageable size, Wallner applied a two-stage intermediate clustering step.

The clustering aimed at finding 100 clusters, representing the structural space of the initial models. The Foldit players generated on average 166,000 models per target, but due to memory and time-constraints the clustering protocol was limited to at most 30,000 input models. Thus, before clustering the initial model ensemble had to be reduced, quite significantly, to between 69%-88% of the original ensemble size. The filtering needed to be relatively fast, since the clustering needed to be completed within one day to leave enough time for the other methods further down the pipelines. We therefore applied a Rosetta energy filter (*energy* filter) and a filter based on the RMSD distance to the model with lowest Rosetta energy (*rmsd1* filter). It was observed that some models were almost identical, probably a result from players sharing and working on the same model. This would add an unwanted bias so these models were excluded before applying the *energy* and *rmsd1* filters, by requiring that models with almost identical Rosetta energy (energy difference < 0.01) need to be at least 0.1Å different.

To find the *energy* and *rmsd1* filter cutoffs that would filter the required number of models, energy cutoffs ranging from median energy to lowest energy in 10 steps and RMSD distance to the lowest energy model in the range 5Å-10Å in 1Å steps were used. This filtering step ended up with around 30,000 models. The combination with the most relaxed cutoffs (highest energy and RMSD distance cutoff) for which 30,000 models passed the filters was chosen. Finally, four clustering runs with cluster radii 0.5Å, 1Å, 2Å, and 3Å were performed using Rosetta's clustering application and cluster centers from the run with the total number of cluster centers closest to 100 were selected.

*Refinement step*

The sets of cluster centers were refined using a modified version of GalaxyRefine [5,6]. The refinement method is composed of three steps of initial optimization, relaxation, and model selection, as outlined in Figure S2. The number of runs for each model was reduced from 32 to 5 compared to the original method to save computational cost because a large number of models

(~100) needed to be refined in the WeFold experiment.

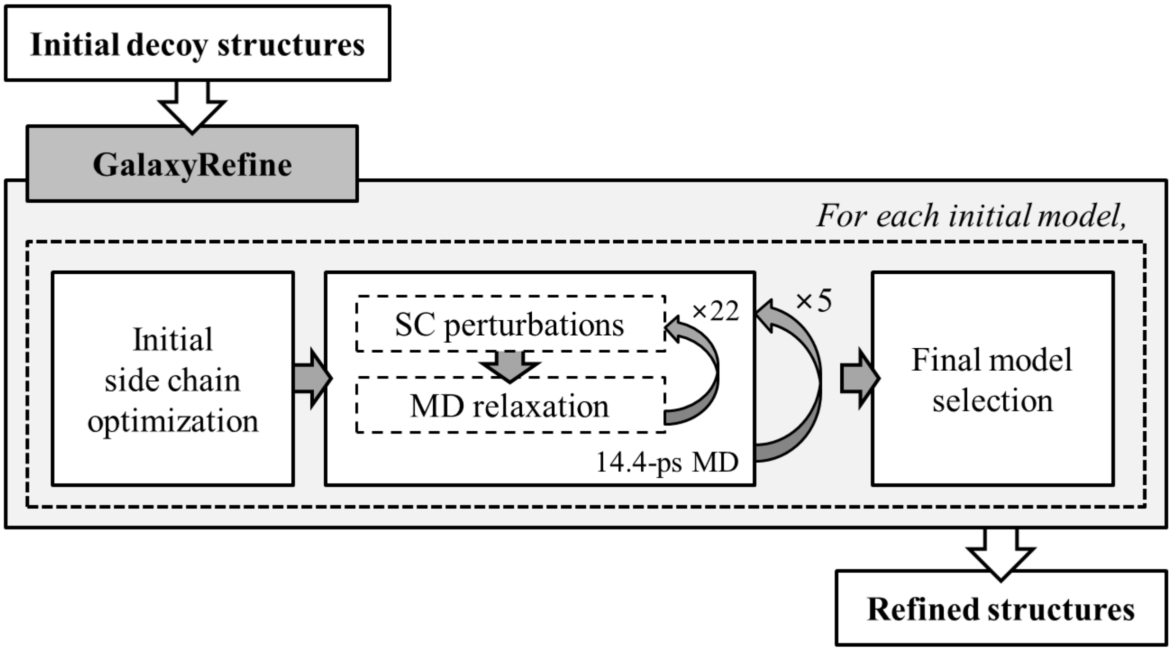


**Figure S2.** Modified GalaxyRefine flow chart for WeFold.

Each model was first subjected to side chain optimization based on a graph-theory algorithm, and then relaxed by 22 times of side chain perturbations and subsequent short (0.6-ps) molecular dynamics (MD) simulations with a 4-fs time step. The total MD simulation time for this run is 14.4-ps including 1.2-ps of pre-relaxation. For each initial model, 5 runs of the 14.4-ps relaxation were performed, and the lowest-energy model out of the 5 final MD snapshots was selected. The energy function for GalaxyRefine [5,6] consists of physics-based energy terms such as the CHARMM22-based molecular mechanics energy [7], FACTS solvation free energy [8], and knowledge-based energy terms such as hydrogen bond energy [9], dipolar DFIRE potential energy [10], and side-chain [11] and backbone torsion angle energy. The same energy function was used for side chain optimization and MD simulation.

*Selection steps*

The refined models were finally assessed and ranked by McGuffin's ModFOLD5_single method and Wallner's ProQ2 [12] method, which are described further below. We call the former pipeline *wfKsrFdit-BW-Sk-McG* and the latter *wfKsrFdit-BW-Sk-BW*. In pipeline *wfKsrFdit-BW-Sk-McG*, Zhang's models were additionally added to the pool of models for final ranking with ModFOLD5_single from target T0795 onwards.

***Selection by the ModFOLD5_single method.*** The latest version of the ModFOLD server [13,14,15] (ModFOLD5) was used to score and rank the final set of models for the wfKsrFdit-BW-Sk-McG pipeline. The ModFOLD5 server is capable of working in quasi-single model

mode or in multiple-model/clustering mode. The first stage of the algorithm generates ~115-130 Tertiary Structure (TS) models using the multi-template approach [16] which forms the basis of the IntFOLD3 server [17]. Usually, in the default server mode (ModFOLD5), a straightforward clustering approach is used whereby all submitted models are pooled together with the IntFOLD3 TS models and clustered using ModFOLDclust2 [18]. The global QA score output from ModFOLDclust2 is simply the mean of the global QA scores obtained from the ModFOLDclustQ method and the original ModFOLDclust method [13,19]. ModFOLDclustQ is similar to the previous ModFOLDclust method, however with ModFOLDclustQ a modified version of the structural alignment free Q-measure [20] is used instead of the TM-score [21], which is used by ModFOLDclust, in order to carry out all-against-all pairwise model comparisons.

The ModFOLD5 server also operates in a quasi-single model mode (ModFOLD5_single) whereby each 3D model is compared in isolation against the pool of reference IntFOLD3 models using a global and local scoring approach similar to that used for ModFOLDclust2. However, for the WeFold pipelines, because we were just submitting models for the Human/All groups targets, this meant that all of the CASP11 server models were available to us prior to submission. We therefore used the server models as the ModFOLD5_single reference comparison set for all submissions, instead of just the IntFOLD3 models. Following ranking with ModFOLD5_single, the per-residue error estimates, or local QA scores, were taken directly from ModFOLDclust and were added to the B-factor column in each model file. Finally, the top 5 models, ranked according to the ModFOLD5_single global score, were submitted.

***Selection by the ProQ2 method.*** A new implementation of ProQ2 [22] as a scoring function in Rosetta was used to score and rank the final set of models for the *wfKsrFdit-BW-Sk-BW* and *wfZhng-Sk-BW* (described in 2.1.2) pipelines. ProQ2 is a single-model method that estimates model accuracy using a support vector machine (SVM) to predict the local quality of a protein model by combining structural and sequence-based features calculated over a sequence window from the model (see the references for details). The local quality is measured by S-score [23] $S_i=1/(1+(d_i/3)^2)$, where $d_i$ is the distance deviation of residue in a superposition that maximize the sum of $S_i$ over the whole protein. For the case of ranking the local predicted $S_i$-scores are summed to a global score from which the top 5 models are selected. In the official quality assessment category in CASP11, ProQ2 was one of the best if not the best single-model quality assessment method [24].

***Foldit Players Pipelines: wf-AnthropicDreams, WeFold-Contenders, WeFold-GoScience, wf-Void_Crushers, and WeFold-Wiskers***

Five Foldit-based teams requested the ability to select and submit their own CASP11 submissions from a pool of their own team's solutions. Each of these five teams was provided with any top-scoring solutions produced by team members, as well as team solutions that were flagged for special interest. This was a new feature added to Foldit after CASP10: a 'Share with

Scientists' button, allowing players to flag a particular prediction as interesting (even if it was not a top-scoring Foldit solution).

As Foldit does not allow different teams to share solutions with one another, these five CASP11 teams were completely independent from one another. In the case that Foldit players built on previous work by other WeFold contributors (e.g. Keasar's selection of server models), each Foldit-based team submitted predictions under a specialized WeFold alias: *wf-AnthropicDreams*, *WeFold-Contenders*, *WeFold-GoScience*, *wf-Void_Crushers*, and *WeFold-Wiskers*.

### *The Zhang pipelines: wfZhng-Ksr and wfZhng-Sk-BW*

Zhang provided decoys for (assumed) single domain targets. These decoys had been generated by three pipelines: (1) I-TASSER server [25], (2) QUARK server [26], and (3) an interplay pipeline of I-TASSER and QUARK [27]. While the I-TASSER pipeline used a uniform template-based protocol for all targets, QUARK ran the *ab initio* protocol for hard targets and the template-based protocol for easy ones, where the category of the targets was decided automatically by the LOMETS threading programs [27,28]. These decoys should not be confused with models generated by Zhang's human group, which was also based on an automated approach, but using models from other CASP servers [29,30]. In each pipeline, the initial conformations generated by the structure assembly simulations were clustered by SPICKER [31] and then refined by FG-MD [32]. Sets of 400-1550 full-atom models per target were pooled from the clusters of the three pipelines, and were made available to the WeFold groups at http://zhanglab.ccmb.med.umich.edu/decoys/casp11/. Due to the limited accuracy of the domain parsers, in particular for the hard targets [33], the Zhang group provided only the decoy sets for those targets that were deemed to be of a single domain by the servers.

Zhang's models were clustered by the Wallner group using the clustering application in Rosetta with cluster radii 0.5Å, 1Å, 2Å, and 3Å. No filtering prior to clustering was needed here since the total number of models was much smaller than in the case of Foldit. The cluster centers from the run, with the total number of cluster centers closest to 100 (based on the number of structures that the QA/scoring groups could handle at the time), were selected and served as the basis for two pipelines: *wfZhng-Ksr* and *wfZhng-Sk-BW*.

The *wfZhng-Ksr* pipeline simply aimed to select the best five models out of the cluster centers, using MESHI-score, and submit them. In the alternative *wfZhng-Sk-BW* pipeline, Seok Lab refined the representatives from each cluster using GalaxyRefine [5] and the Wallner group selected the best 5 using ProQ2 [12].

### *UNRES pipelines: wfCPUNK and wf-Baker-UNRES*

There were 2 UNRES-based pipelines: *wfCPUNK* and *wf-Baker-UNRES* and they mainly

differed in the distance restraints they used which were provided by (a) Floudas' lab and (b) Baker's lab respectively. The *wfCPUNK* procedure used was similar to that used during the CASP10 exercise [34] with modifications resulting from updates of its components. The *wf-Baker-UNRES* pipeline was a new addition in CASP11 that started submitting targets from target T0812 onwards and, consequently, only submitted results for 10 targets.

(a) For the wfCPUNK pipeline, the consensus secondary structure (SS) SVM model, conSSert [35], was utilized. conSSert uses as features the probabilities for coil, helix, and strand as predicted by PSSPRED [36], PSIPRED [37], RAPTORX [38], and SPINE-X [39]. conSSert consists of 3 one vs. all binary classifiers that are combined to provide a 3-class prediction. conSSert has been shown previously to provide significant improvements in the prediction of ordered secondary structure, especially for the prediction of strands [35].

Tertiary contact prediction for the wfCPUNK pipeline was based on a consensus-template based approach. The procedure began with a SPARKS-X [39] run for the full target sequence and the calculation of a normalized Z-score, based on alignment span. The normalized Z-score allows for the identification of potential protein domain boundaries based on the definition of protein domains in the template library, and was utilized for the subsequent splitting of the target sequence into domains. SPARKS-X was used to produce a ranked list of templates for the sequence segments of each identified domain. Delaunay triangulation was applied to identify $C^{\beta}$ contacts in the top 25 structural templates. A consensus-score, based on a summation of the Z-scores for every template in which a given contact was observed, was used to rank the contacts. For a given target, the consensus-scores were normalized by the maximum observed value, so that the scores range between 0 and 1.

(b) All CASP related submissions to the GREMLIN webserver [40] were made public and provided to WeFold community. In addition to the predicted contacts, the webserver provides an overlay of the contacts on the top 10 HHsearch hits. If the top HHsearch [41] hits did not make a large portion of the predicted contacts, these targets were deemed worthy for human intervention. For each target multiple attempts were made to create an optimal alignment for contact prediction. This includes trimming the target sequences to conserved regions, trying different e-value cutoffs, iterations and different alignment generation software. The first scan of e-values was made using HHblits [42] using the UniProt [43] database from 2013_03. If not enough effective sequences were found (< 5 sequences per length) Jackhmmer [44] with a newer UniRef90 database from 2014_04 was used. The e-values tried include 1E-04, 1E-06, 1E-10, 1E-20 and 1E-40. The iterations tried include 1, 2, 4 and 8. The default was 1E-10 with 4 iterations. After manual inspection of the results, the GREMLIN authors shared their preferred predictions on the WeFold forums.

The restraints were imposed on the virtual-bond dihedral angles between the consecutive $C^{á}$ atoms and virtual side-chain distances, respectively. The backbone virtual-bond-dihedral-angle restraints were assigned based on the secondary structure predicted by conSSert [35] (as described above). The boundaries of angle restraints were from $30^{o}$ to $70^{o}$ for the helical and

from -220$^o$ to -140$^o$ or from 140$^o$ to 220$^o$ (or, alternatively, a continuous interval from 140$^o$ to 220$^o$ when shifting the dihedral-angle interval to [0$^o$,360$^o$]) for extended structure. A quaternary penalty function of the form given by eq. 1 was applied

$$U_{restr} = \begin{cases} \frac{1}{4}k(\gamma - \gamma_{\min})^4 & \text{for } \gamma < \gamma_{\min} \\ \frac{1}{4}k(\gamma_{\max} - \gamma)^4 & \text{for } \gamma > \gamma_{\max} \\ 0 & \text{otherwise} \end{cases} \quad (1)$$

where $\gamma_{min}$ and $\gamma_{max}$ are the allowed range of the variation of the virtual-bond-dihedral angle $\gamma$ and $k$ is the force constant; we assumed $k$=0.1 in this study, which corresponds to weak restraints.

To determine the distance restraints for the *wfCPUNK* pipeline, the consensus-template contacts were converted into distance restraints with 8 Å adapted as the cut-off distance. The consensus-scores for each contact were multiplied by a factor of 0.01 and used as the weights for the restraints, with the restraint function having the same quaternary form as that for angles (eq 1).

For the *wf-Baker-UNRES* pipeline, distance restraints were derived from the contact prediction carried out with the GREMLIN [40] method. GREMLIN works by constructing a global statistical model that simultaneously captures the conservation and co-evolution patterns in the input multiple sequence alignment. The alignments were generated using HHblits [42] and Jackhammer [45] with varying e-value and number of iterations. Strongly co-evolving residue pairs as identified by this approach were used as restraints in modeling [45].

Sampling was carried out by applying restrained Multiplexed Replica Exchange Molecular Dynamics (MREMD) [46,47,48] simulations with the coarse-grained UNRES force field [49,50,51,52,53] for each of the targets considered. For each target, the MREMD simulation consisted of 64 trajectories run at 32 different temperatures (2 trajectories per temperature, temperature range from 200 to 500 K), each trajectory consisting of 20,000,000 steps with a 4.9 fs time step, replicas exchanged and snapshots recorded every 20,000 steps. Subsequently, 200 last snapshots were taken from each of the trajectories (12,800 snapshots total) and Weighted-Histogram Analysis Method (WHAM) [54] was used to calculate the ensemble averages and probabilities of the conformations, as described in our earlier work [55]. The heat-capacity graph was computed and analyzed and the clustering temperature $T_c$ selected (usually 20 K less than that of the major heat-capacity peak) to carry out subsequent analysis.

The Ward's minimum-variance method [56] was used to obtain 5 clusters corresponding to the 5 models to be submitted to CASP. These clusters (models) were ranked according to their decreasing summary probability, as described in our earlier work [55]. For each cluster, the average structure (each component conformation being weighted by using its probability calculated by WHAM at $T_c$) was subsequently computed and the conformation of the cluster

closest to the respective average structure was selected as the respective coarse-grained model. The final models, which were subsequently submitted to CASP, were obtained by converting the coarse-grained models to all-atom structures using the PULCHRA method [57] and SCWRL [58].

### *The PTIGRESS pipelines: wfHHpred-PTIGRESS and wfKeasar-PTIGRESS*

The following pipelines were constructed with the goal to refine the models predicted by the HHpred-A method and by Keasar's selection of server models, which both performed well in CASP10. In wfHHpred-PTIGRESS, Model 1 from HHpred-A was selected for refinement by Princeton_TIGRESS [59]. If the size of the structure was ≤ 154 residues, 5 refinements of the starting HHpred-A model were submitted, using the full Princeton_TIGRESS pipeline. If the size was > 154 residues, 1 model was submitted, using only the MD portion of Princeton_TIGRESS. The cutoff of 154 was shown previously [59] to be optimal in distinguishing when to use the Rosetta FastRelax [3] method vs. molecular dynamics to maximize refinement by GDT_TS. In wfKeasar-PTIGRESS, Princeton_TIGRESS refined the 5 server models scoring highest according to the Keasar method. Princeton_TIGRESS [59] was enhanced for CASP11 to include an improved SVM classifier of refined and degraded structures, as well as an improved MD protocol to handle larger structures. Among refinement methods currently implemented as servers, Princeton_TIGRESS was found to demonstrate the most consistent (highest # GDT_TS > 0) refinement, improving Model 1 78% of the time, as well as the most substantial net refinement (highest $\sum_{i=1} GDT_i$), in blind predictions in the refinement category during CASP11 [60].

### *Mixed pipelines: wfAll-Cheng, wfAll-MD-RFLB, wfMix-KFa, wfMix-KFb*

There were several pipelines that selected from a combination of server, Keasar-Foldit, and Zhang models, including: (a) *wfAll-Cheng*, (b) *wfAll-MD-RFLB*, (c) *wfMix-KFa*, (d) *wfMix-KFb*, (e) *wfMix-KPa*, and (f) *wfMix-KPb*. The wfAll-MD-RFLB pipeline is described below.

*(a) wfAll-Cheng*

The *wfAll-Cheng* method collected models generated by all CASP11 servers and WeFold methods. Each chain was extracted from the multi-chain models as a single model. Models with incorrect information, such as incorrect residue numbers and names or incorrect PDB format, were filtered out. A fully pairwise model comparison tool, APOLLO [61], was used to evaluate all the models. APOLLO calculates a GDT_TS score (Global Distance Test Total Score) [46] between one model and each of other models using the TM-score [21] program. The predicted score of a model is the average GDT_TS score between it and all the other models. The top five models with highest scores were then refined using i3Drefine [62,63], which employs hydrogen-bonding network optimization and atomic-level energy minimization using a composite physics

and knowledge-based force field. The residue-level local quality of the refined models was assessed by ModFOLDclustQ [18], a novel model quality assessment program that compares 3D models of proteins without the need for CPU intensive structural alignments. The residue-specific local quality scores were added into the refined top models. The energy-minimized models were submitted as final predictions.

*(b) wfAll-MD-RFLB*

The wfAll-MD-RFLB pipeline employed the dominance criterion to rank models generated by other WeFold pipelines. This criterion can rank the models using conflicting metrics, called objectives, simultaneously.

Model A is said to dominate model B when both the following conditions are satisfied:

1. Model A is not worse than B in any objective;
2. Model A is strictly better than B in at least one objective.

The ranking of each model is defined by performing all possible pairwise comparisons and calculating the number of other models it dominates. Additionally, all solutions which are not dominated by any other composes a set, called Pareto-optimal, that meets an equilibrium situation between the conflicting metrics [64,65].

Each model from a large dataset of structures generated by other WeFold pipelines was submitted to a two-step energy minimization with the steepest descent algorithm. While in the first step no constraints were applied to the protein, in the second one all covalent bonds were constrained with the LINCS algorithm [66]. The calculation of potential energy and solvation energy was carried out with the GROMACS 4.6.5 [67] suite using the AMBER99SB-ILDN force-field [68]. The GBSA implicit solvation model [69] was used with the OBC algorithm for calculating the Born radii [70]. The potential and solvation energy were used as objectives and the dominance among models was calculated by the 2PG Sort Dominance Front software. This method does not rely on similarity between the target and other proteins.

(c-d) *wfMix-KFa* and *wfMix-KFb*

The *wfMix-KFa* and *wfMix-KFb* pipelines made use of two different versions of Seder, V1.0 and V2.0 [71]. While Seder V1.0 takes into account the distribution of inverse distances delineated into residue types, the newer version of Seder makes additional distance classes and uses these refined distributions to provide an estimate for the closeness of a given model to the native x-ray conformation. Briefly, Seder calculates the distribution of inverse distances associated with a given model. It then uses machine learning [72] to process this information. In the training phase the inverse distance distributions of a given model are mapped to the TM-Score [21] of that model to the native structure of the same sequence. For the WeFold collaboration two dedicated pipelines were set up. For initialization purposes, and since sometimes WeFold models were unavailable for a given target, only the server models submitted to CASP were initially used. As WeFold models became available they were downloaded from the WeFold site and were used

instead of the CASP server models. These models were then ranked using the Seder V1.0 and Seder V2.0 scoring functions.

(e-f) *wfMix-KPa* and *wfMix-KPb*

The pipelines *wfMixKPa* and *wfMixKPb* were based on an extension and improvement of the MQAPsingle methodology that was among the most successful Model Quality Assessment techniques in CASP10 experiment [73]. This program submits the target sequence to the GeneSilico Fold prediction metaserver [74] to collect approximately one hundred of 3D models for the target protein. In parallel, it executes the following three modules. First module predicts secondary structure, solvent accessibility and contact maps for the target sequence using third-party methods. These predictions are compared with values of the corresponding features calculated directly from the 3D structural models by the DSSP program [75]. These (dis)agreement terms, together with in-house implementation of the DFIRE [76] statistical potential and the number of unsatisfied hydrogen bond donors/acceptors, are used to estimate GDT_TS score [77] of each of the input and reference models. The second module calculates the all possible pairwise comparisons between the input models and the reference models *only*. Two measures of similarity between a pair of models are applied: GDT_TS and Q-score [78,79], the latter measures the structural similarity between two models by comparing their internal residue distances. Then, 3D-Jury algorithm [80] is applied to calculate the consensus scores of the input model(s). The third module is based on an assumption that values of "pure" single-model scoring function, on average, decrease as models become more similar to the native structure. Thus, the model that is the closest to the native structure should provide the highest correlation coefficient of a score (provided by such a single-model MQAP) versus distance, when used as the reference in pairwise comparisons with the remaining models [81]. Finally, to predict the GDT_TS score of the input model(s), the primary scores provided by the above-mentioned three modules and a linear regression algorithm were used.

## Description of WeFold2 Refinement Pipelines

### *wfFdit-BW-KB-BW, wfFdit-K-McG, wfFdit_BW_K_SVGroup, and wfFdit_BW_SVGroup*

The refinement pipelines were created based exclusively on models generated by Foldit players who were given the starting models provided by the prediction center. For the *wfFdit-BW-KB-BW*, *wfFdit-K-McG*, and *wfFdit_BW_K_SVGroup* pipelines, refinement models were constructed using Foldit, then filtered and clustered by Wallner using the procedure described in 2.1.1.2, then refined with KoBaMIN [82], and finally ranked using the ProQ2, ModFOLD5_single, and the PSN-QA methods, respectively. ProQ2 and ModFOLD5_single are described in subsection "Selection step" and PSN-QA is described below. The pipeline wfFdit_BW_SVGroup is similar to wfFdit_BW_K_SVGroup, but chooses from unrefined clusters rather than from KoBaMIN-refined clusters, as wfFdit_BW_K_SVGroup does.

***Selection by the PSN-QA method.*** The Protein Side-chain Network-Quality Analysis (PSN-QA)

ranking tool is based on a graph-theoretical translation of a protein structure based primarily on its side-chain connectivity network. A detailed methodology of the derivation of the protein graph is available in the literature [83]. In brief, a PDB file is read as the primary input and based on side-chain atom-atom distance cutoff of 4.5Å, a connection is defined between two residues, separated by at least one amino acid (i+/-j >1). The number of such atom-atom connections between any two amino acid pairs is then normalized by the geometric mean of normalization values of those residues which were derived from a database of high-resolution protein structures [84]. Mathematically this is represented as:

$$I_{ij} = n_i * 100 / \mathrm{sqrt}(N_i * N_j)$$

where $I_{ij}$ is the interaction strength between the residue pairs, $n_{ij}$ is the number of sidechain atom pairs within a distance cut-off of 4.5 Å, between residues i and j, and $N_i$ and $N_j$ are the normalization values for residues i and j.

A PSN thus constructed results in an n*n matrix of varying interaction strengths ($I_{ij}$). The next step is to segregate this raw-matrix into bins of binary adjacency matrices where certain $I_{ij}$ satisfying minimum interaction strength ($I_{min}$) are converted to ones and the remaining are converted to zeroes. The resulting adjacency matrices at each $I_{min}$ hold crucial information regarding the protein residue connectivity like size of the largest cluster, size of the top 3 k-1 & k-2 communities, and many other network parameters as a function of $I_{min}$ [85,86]. The transition of these parameter values over $I_{min}$ follows a common pattern for native proteins and becomes strikingly different for decoy structures. This has been used to train a SVM model on good native proteins (5422) and bad decoys (20000+) to recognize a decoy whose side chain connections are native like in their global connections or not [87,88]. The PSN-QA tool provides a rank value based on which the models are sorted and ranked. An advantage of this method is that the scores are derived based on the inherent nature of folded proteins and does not require a comparison with experimentally derived structures. PSN-QA uses information derived from interactions of side-chain atoms to judge the quality of a predicted model. Hence, the use of PSN-QA score in selecting native-like structure is most effective on models that have significant backbone overlap with the template structure. Apart from PSN-QA, recently a new method called Network Similarity Score (NSS) which compares protein structures based on the spectral properties of weighted protein networks, has been developed [89,90]. This method captures global changes in the protein structure from subtle variations in the side chain orientations. NSS can serve as a powerful structure validation tool in future, in comparing atomistic details of side chain interactions.

## Description of the WeFold3 Pipelines

The pipelines of the third WeFold round, during CASP12, can be divided into four main categories based on their initial decoy generators: Rosetta, UNRES-generated, CASP12 server

models, and a combination of CASP12 servers and WeFold models.

***The Rosetta pipelines: wfDB_BW_SVGroup, wfRosetta-MUfold, wfRosetta-ProQ-MESHI, wfRosetta-ProQ-ModF6, wfRosetta-Wallner, and Rstta-PQ-MESHI-MSC***

These pipelines shared their initial components, splitting only in their last steps (Figure 2). They started with BAKER-ROSETTASERVER decoys of target domains generated by the Rosetta@home distributed computing project (http://boinc.bakerlab.org/rosetta). These pipelines differ from BAKER-ROSETTASERVER in model selection, refinement, and domain assembly. Pipeline steps prior to decoy generation such as domain boundary prediction and difficulty prediction assessment, PDB template detection, sequence /structure alignment, contact prediction, and restraint generation were carried out by BAKER-ROSETTASERVER. All domains were modeled using the Rosetta comparative modeling protocol (RosettaCM) [91], and difficult domains, were also modeled using the Rosetta fragment assembly methodology (RosettaAB) [92]. As ProQ2 has been integrated recently into Rosetta [93], quality assessment scores for each decoy were calculated directly from Rosetta generated silent files without the need to extract millions of PDB files. Silent files are Rosetta-specific file formats used for efficient concatenated storage of large numbers of structures. In total, 32,474,636 decoys were generated by BAKER-ROSETTASERVER and scored using ProQ2 during CASP12.

The Rosetta sets included hundreds of thousands of decoys per domain, just like the Foldit sets had in WeFold2. However, rather than applying filtering and clustering to reduce the complexity like they did in WeFold2, the Wallner group filtered each set by selecting the top 1,000 decoys according to the ProQ2 score. There was a set of 1,000 decoys per protein domain as identified by BAKER-ROSETTASERVER.

The resulting 1,000 or more decoys were then scored using different quality score algorithms in the different pipelines. In the *wfDB_BW_SVGroup* branch, the SVLab group used their SVM-based algorithm; the *wfRosetta-MUfold* branch used MUFOLD for the selection. The *wfRosetta-ProQ-MESHI* and *Rstta-PQ-MESHI-MSC* branches used two different scoring functions that utilize the same features but different machine learning approaches [1]. Finally, the *wfRosetta-ProQ-ModF6* branch used ModFOLD6, and the *wfRosetta-Wallner* branch just used ProQ2. Another major difference between the pipelines was the way they handled the splitting of the target protein to domains when applicable. Some of the pipelines simply failed to handle the domain issue and submitted the decoys as independent domains, but this consequently negatively affected their CASP performance on multidomain targets.

***The UNRES pipelines: wf-BAKER-UNRES and wfCPUNK***

These pipelines are similar to those used during the WeFold2 exercise with modifications resulting from updates of their components. Please refer to Section 2.1.4 for a brief description and to the Supplementary Materials for a more detailed description.

***Mixed pipelines: wfAll-Cheng and wfRstta-PQ2-Seder***

The *wfAll-Cheng* pipeline is similar to the WeFold2 pipeline of the same name except that the average ranking of APOLLO and Qprob[94] (a single model quality assessment method) was used to select models in WeFold3. Please refer to Section 2.1.6 for a brief description and to the Supplementary Materials Section for a more detailed description. *wfRstta-PQ2-Seder* uses Seder v1.0 to pick from a pool of candidate protein models obtained from a combination of all CASP12 submitted server models and the Rosetta models selected by ProQ2 for the Rosetta pipelines.

***The server models pipelines: wfMESHI-TIGRESS and wfMESHI-Seok***

These pipelines differ in the refinement and final selection methods used. They started with the entire set of CASP server models, which were scored by MESHI_Score [1]. The *wfMESHI-TIGRESS* pipeline is similar to the *wfKeasar-PTIGRESS* pipeline in WeFold2, which was briefly described in section 2.1.5. Both pipelines use the same TIGRESS refinement algorithm [59]. No re-ranking of models was performed after refinement, meaning that the refined version of the model ranked 1 by MESHI was submitted as *wfMESHI-TIGRESS* model 1.

The *wfMESHI-Seok* branch was newly tested in WeFold3. The CASP server structures were scored by MESHI_Score [1] and the number of structures to be refined was reduced to 48 (due to time constraints) by taking those with the highest MESHI scores that are structurally distinct with mutual TM-score lower than 0.95. The selected structures were refined with an improved version of GalaxyRefine [5,95], which includes a newly developed knowledge-based potential that considers solvation states of interacting atoms as well as their distances. The refined models were ranked by the new potential, and the top five models were submitted as final predictions.

## Large Scale Analysis of WeFold2 Pipelines

Figures S3-S20 show box and whiskers plots for each one of the steps in the Keasar-Foldit pipelines. Each figure corresponds to one of the submitted targets. These pipelines start with the server models that are released by the CASP organizers (stage 1 and stage 2). These steps correspond to the first two columns in the figures, which is labeled srvr 1 and srvr 2 in the x-axis. Besides the name of the component in the pipeline, the x-axis shows the number of models that are created or handled at each step. From the server models, Keasar selects a subset of 10-20 models which are marked as dots in the third column and Khatib selects 1-5 of them which are given to the Foldit players. Khatib's selected models are marked as triangles. The fourth column shows the box and whiskers plot for the hundreds of thousands of models generated by the Foldit players. In each figure the GDT_TS value of the best model submitted to CASP11 considering all CASP teams is marked with a dashed green line, which is labeled with the name of the team that submitted the best model. These plots show that some of the models generated by the players are better than the best model submitted to CASP11 by all groups in most cases. The fifth column shows a box and whiskers plot for the cluster representatives calculated by Wallner's method and the sixth column shows the GDT_TS values of the same models refined by the GalaxyRefine method. These GDT_TS values of the clusters are slightly improved by the

refinement algorithm as it can be seen in the plots. The columns that follow show the selection by the different QA algorithms. The 5 models submitted by each group are marked in colors according to whether they were submitted as model 1, 2, 3, 4, or 5. Columns 7th and 8h show the selection by Wallner/ProQ2 (pipeline *wfKsrFdit-BW-Sk-BW*) and by ModFOLD5_single (pipeline *wfKsrFdit-BW-Sk-McG*), which were solely based on Keasar-Foldit generated models for this target. The rest of the columns, 9th-13th show the selection by mixed pipelines *wfMix-KFa*, *wfMix-KFb*, *wfMix-KPa*, *wfMix-KPb*, and *wfAll-Cheng*, respectively.

Box and whisker plots for the WeFold2 Keasar-Foldit Pipelines:

Column 1: Srvr 1 = Servers stage1
Column 2: Srvr 2 = Servers stage2
Column 3: Keasar picks from servers, then Khatib picks from Keasar's selection
Column 4: Foldit players generate models
Column 5: Wallner finds clusters
Column 6: Seok's lab refines clusters
Columns 7-13: Different groups select from these clusters, or from a combination of these and Zhang's clusters, or from a combination of all the models shared by various WeFold groups and servers.

Green line is the best model submitted to CASP11 for that target considering all the CASP11 groups

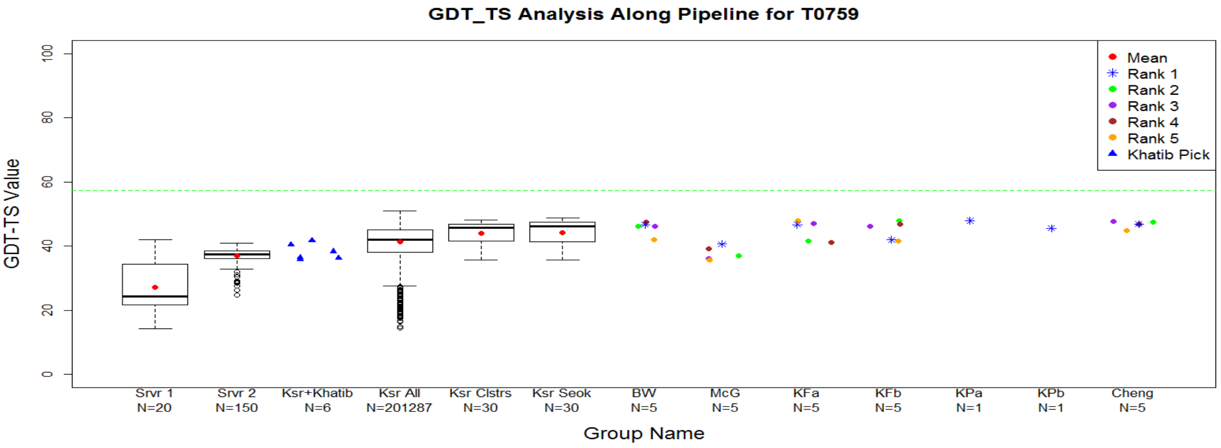

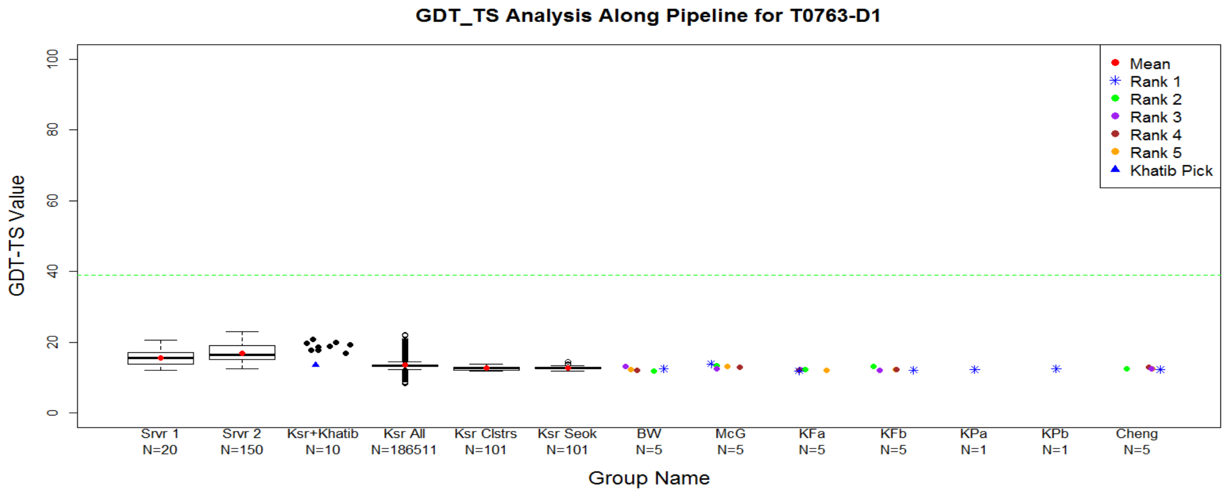
GDT_TS Analysis Along Pipeline for T0763-D1
GDT-TS Value
Group Name
Mean
Rank 1
Rank 2
Rank 3
Rank 4
Rank 5
Khatib Pick
Srvr 1 N=20
Srvr 2 N=150
Ksr+Khatib N=10
Ksr All N=186511
Ksr Clstrs N=101
Ksr Seok N=101
BW N=5
McG N=5
KFa N=5
KFb N=5
KPa N=1
KPb N=1
Cheng N=5

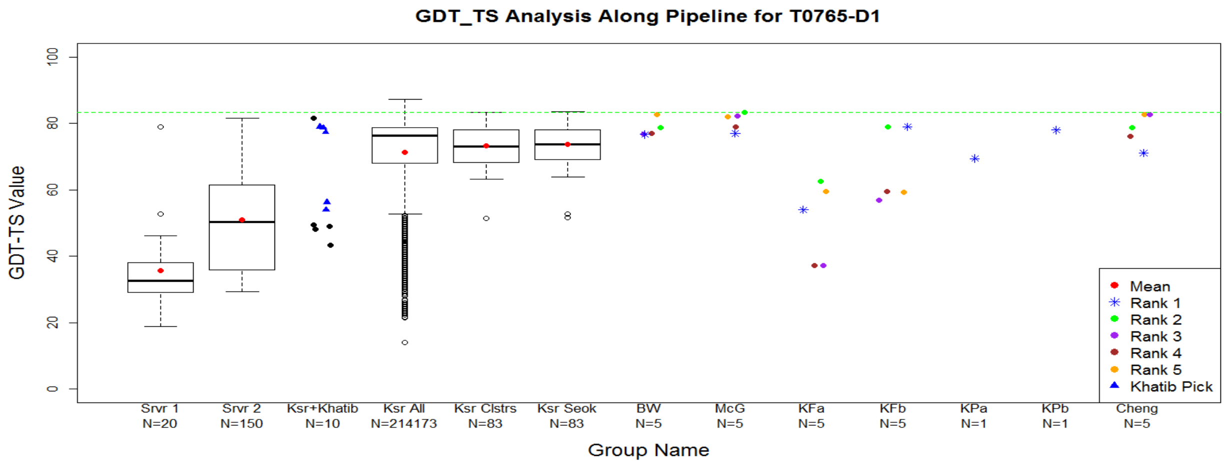
GDT_TS Analysis Along Pipeline for T0765-D1
GDT-TS Value
Group Name
Mean
Rank 1
Rank 2
Rank 3
Rank 4
Rank 5
Khatib Pick
Srvr 1 N=20
Srvr 2 N=150
Ksr+Khatib N=10
Ksr All N=214173
Ksr Clstrs N=83
Ksr Seok N=83
BW N=5
McG N=5
KFa N=5
KFb N=5
KPa N=1
KPb N=1
Cheng N=5

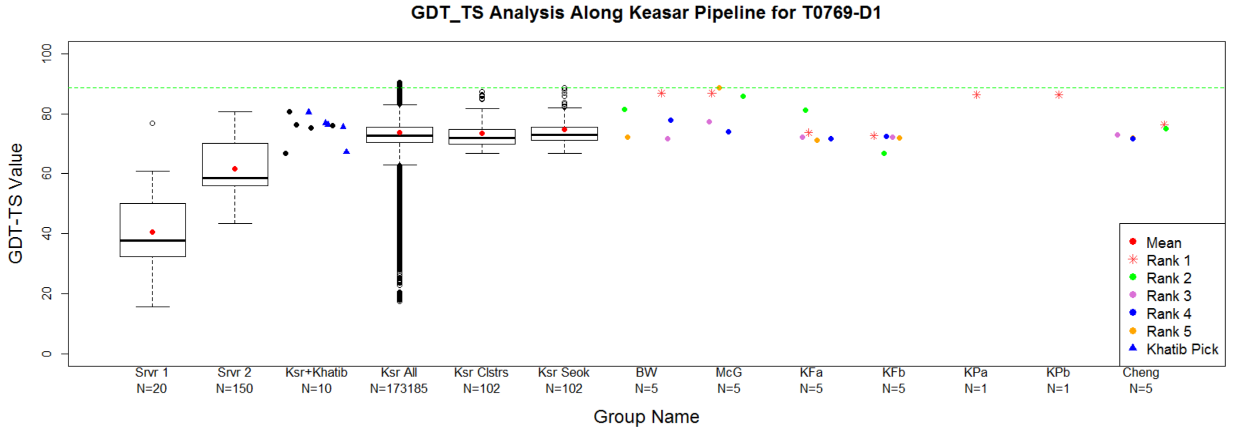
GDT_TS Analysis Along Keasar Pipeline for T0769-D1
GDT-TS Value
Group Name
Mean
Rank 1
Rank 2
Rank 3
Rank 4
Rank 5
Khatib Pick
Srvr 1 N=20
Srvr 2 N=150
Ksr+Khatib N=10
Ksr All N=173185
Ksr Clstrs N=102
Ksr Seok N=102
BW N=5
McG N=5
KFa N=5
KFb N=5
KPa N=1
KPb N=1
Cheng N=5

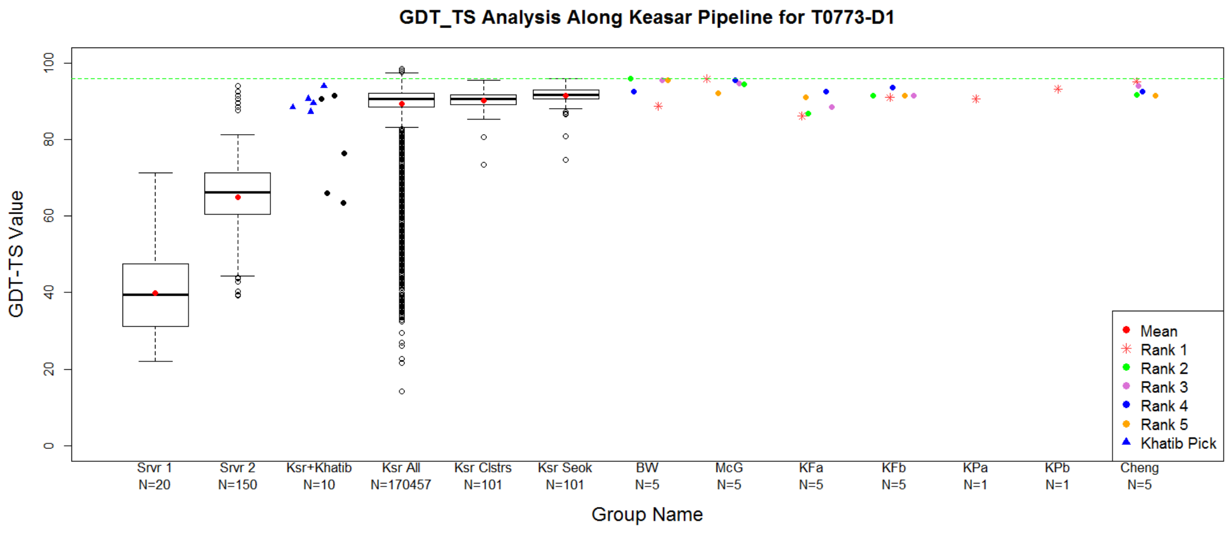
GDT_TS Analysis Along Keasar Pipeline for T0773-D1
GDT-TS Value
Group Name
Srvr 1 N=20
Srvr 2 N=150
Ksr+Khatib N=10
Ksr All N=170457
Ksr Clstrs N=101
Ksr Seok N=101
BW N=5
McG N=5
KFa N=5
KFb N=5
KPa N=1
KPb N=1
Cheng N=5
Mean
Rank 1
Rank 2
Rank 3
Rank 4
Rank 5
Khatib Pick

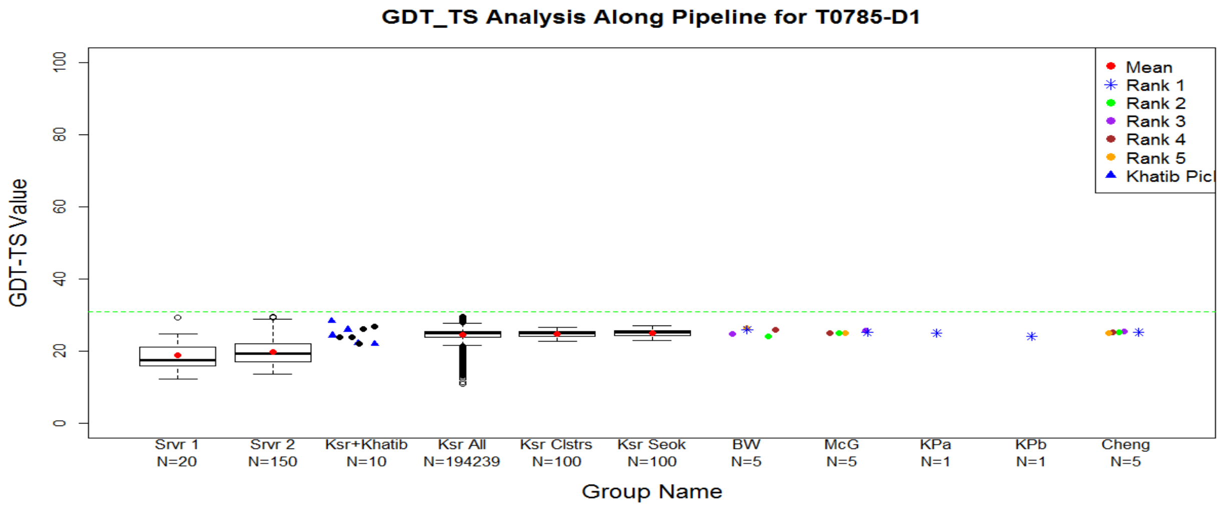
GDT_TS Analysis Along Pipeline for T0785-D1
GDT-TS Value
Group Name
Srvr 1 N=20
Srvr 2 N=150
Ksr+Khatib N=10
Ksr All N=194239
Ksr Clstrs N=100
Ksr Seok N=100
BW N=5
McG N=5
KPa N=1
KPb N=1
Cheng N=5
Mean
Rank 1
Rank 2
Rank 3
Rank 4
Rank 5

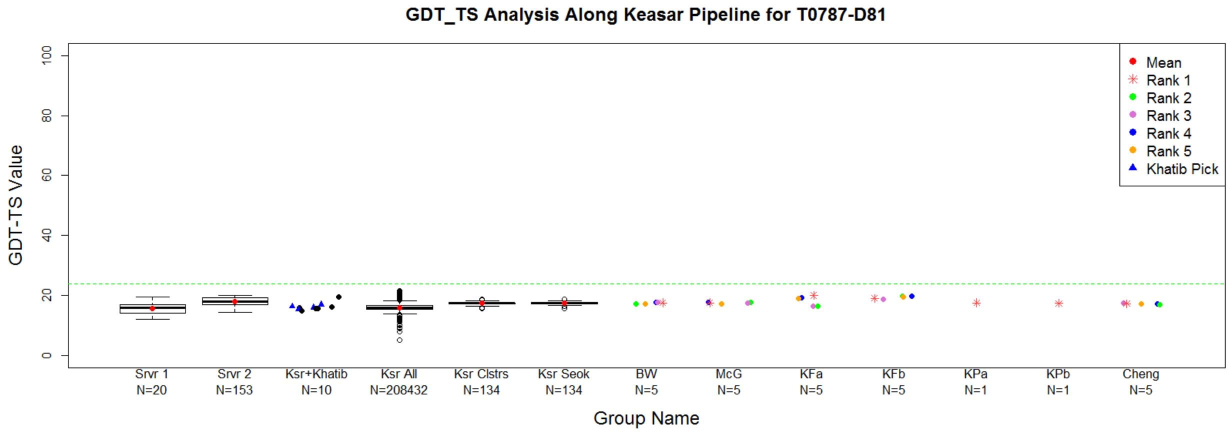
GDT_TS Analysis Along Keasar Pipeline for T0787-D81
GDT-TS Value
Group Name
Srvr 1 N=20
Srvr 2 N=153
Ksr+Khatib N=10
Ksr All N=208432
Ksr Clstrs N=134
Ksr Seok N=134
BW N=5
McG N=5
KFa N=5
KFb N=5
KPa N=1
KPb N=1
Cheng N=5
Mean
Rank 1
Rank 2
Rank 3
Rank 4
Rank 5
Khatib Pick

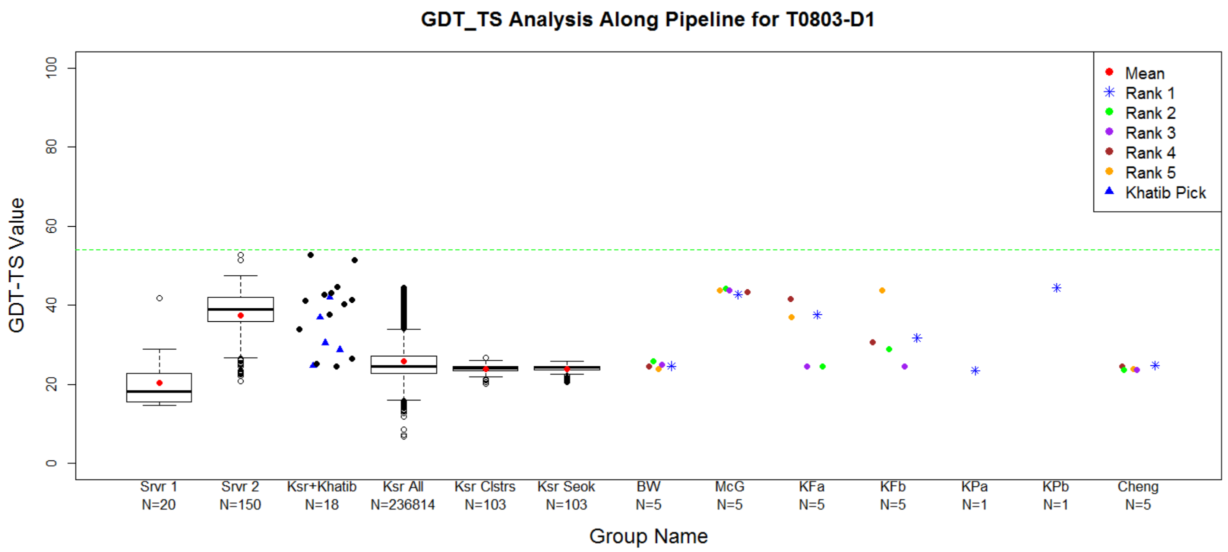
GDT_TS Analysis Along Pipeline for T0803-D1
GDT-TS Value
0
20
40
60
80
100
Mean
Rank 1
Rank 2
Rank 3
Rank 4
Rank 5
Khatib Pick
Srvr 1 N=20
Srvr 2 N=150
Ksr+Khatib N=18
Ksr All N=236814
Ksr Clstrs N=103
Ksr Seok N=103
BW N=5
McG N=5
KFa N=5
KFb N=5
KPa N=1
KPb N=1
Cheng N=5
Group Name

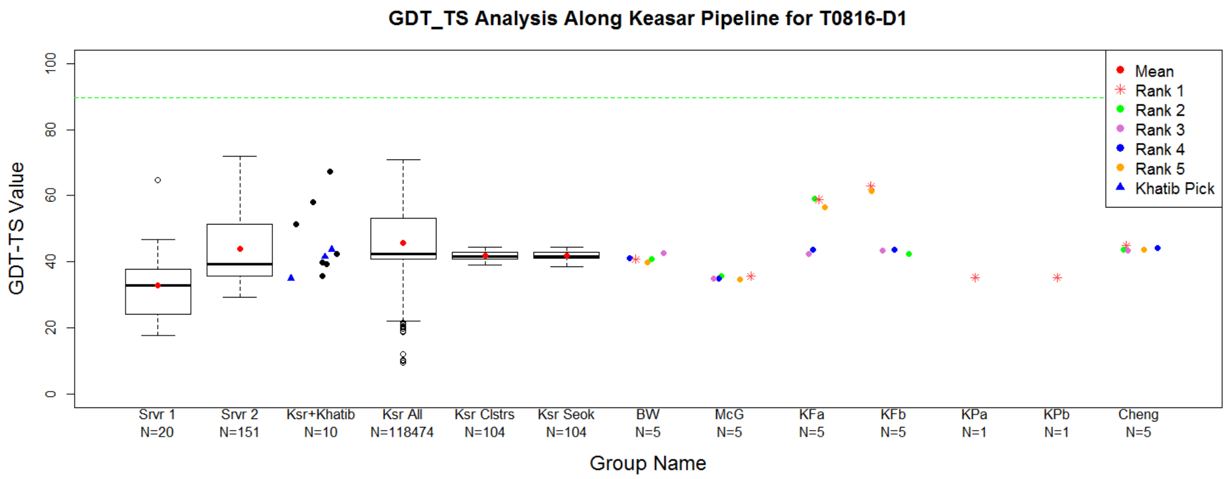
GDT_TS Analysis Along Keasar Pipeline for T0816-D1
GDT-TS Value
0
20
40
60
80
100
Mean
Rank 1
Rank 2
Rank 3
Rank 4
Rank 5
Khatib Pick
Srvr 1 N=20
Srvr 2 N=151
Ksr+Khatib N=10
Ksr All N=118474
Ksr Clstrs N=104
Ksr Seok N=104
BW N=5
McG N=5
KFa N=5
KFb N=5
KPa N=1
KPb N=1
Cheng N=5
Group Name

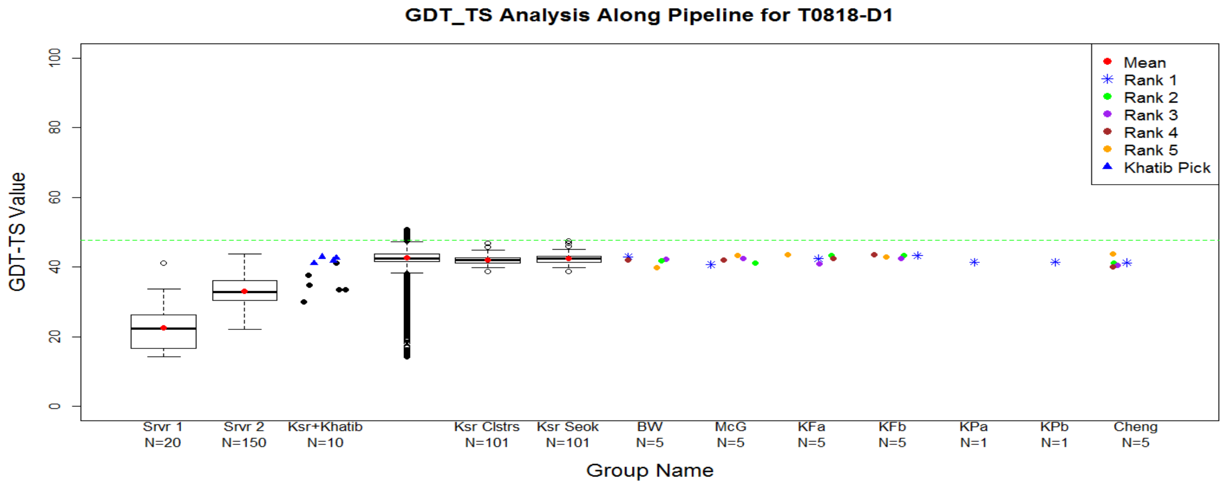
GDT_TS Analysis Along Pipeline for T0818-D1
GDT-TS Value
0
20
40
60
80
100
Mean
Rank 1
Rank 2
Rank 3
Rank 4
Rank 5
Khatib Pick
Srvr 1 N=20
Srvr 2 N=150
Ksr+Khatib N=10
Ksr Clstrs N=101
Ksr Seok N=101
BW N=5
McG N=5
KFa N=5
KFb N=5
KPa N=1
KPb N=1
Cheng N=5
Group Name

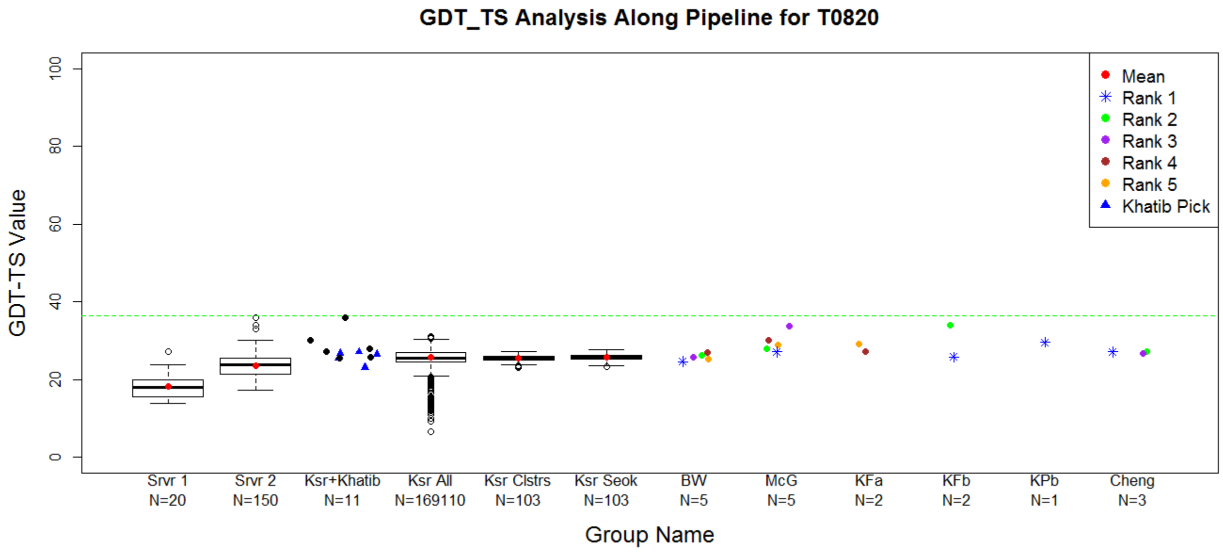
GDT_TS Analysis Along Pipeline for T0820
GDT-TS Value
0
20
40
60
80
100
Srvr 1 N=20
Srvr 2 N=150
Ksr+Khatib N=11
Ksr All N=169110
Ksr Clstrs N=103
Ksr Seok N=103
BW N=5
McG N=5
KFa N=2
KFb N=2
KPb N=1
Cheng N=3
Group Name
Mean
Rank 1
Rank 2
Rank 3
Rank 4
Rank 5
Khatib Pick

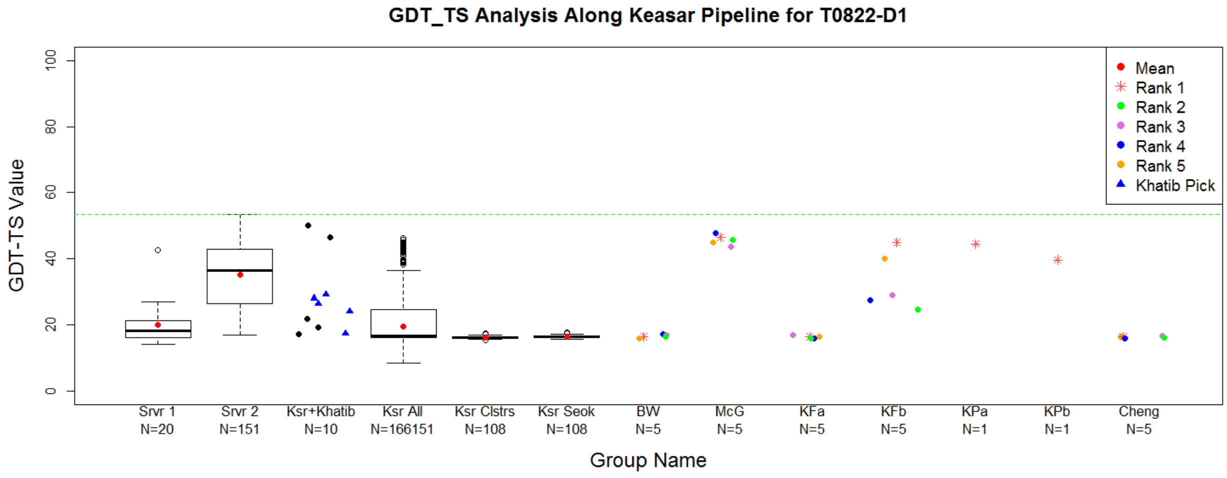
GDT_TS Analysis Along Keasar Pipeline for T0822-D1
GDT-TS Value
0
20
40
60
80
100
Srvr 1 N=20
Srvr 2 N=151
Ksr+Khatib N=10
Ksr All N=166151
Ksr Clstrs N=108
Ksr Seok N=108
BW N=5
McG N=5
KFa N=5
KFb N=5
KPa N=1
KPb N=1
Cheng N=5
Group Name
Mean
Rank 1
Rank 2
Rank 3
Rank 4
Rank 5
Khatib Pick

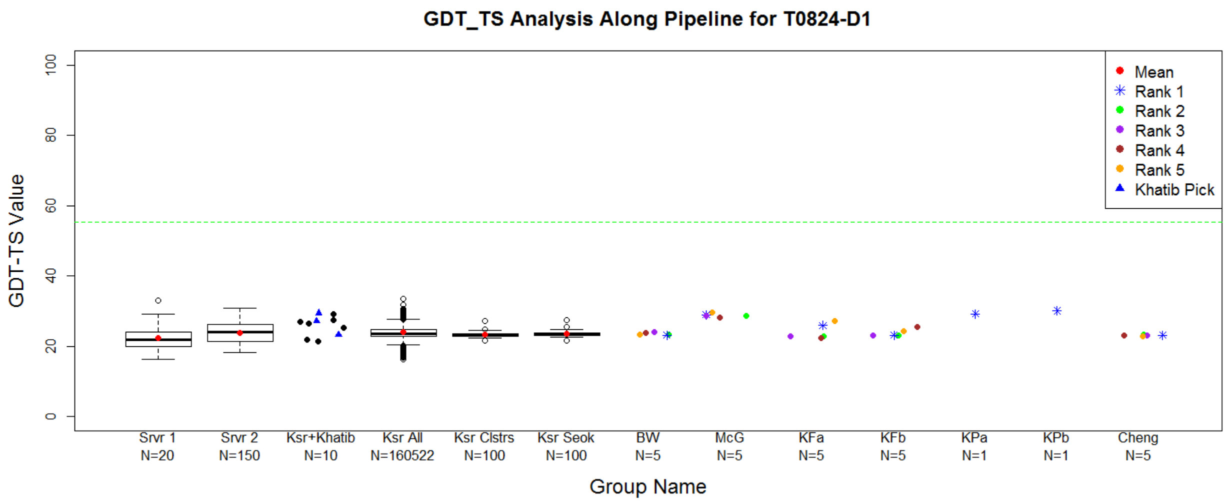
GDT_TS Analysis Along Pipeline for T0824-D1
GDT-TS Value
0
20
40
60
80
100
Mean
Rank 1
Rank 2
Rank 3
Rank 4
Rank 5
Khatib Pick
Srvr 1 N=20
Srvr 2 N=150
Ksr+Khatib N=10
Ksr All N=160522
Ksr Clstrs N=100
Ksr Seok N=100
BW N=5
McG N=5
KFa N=5
KFb N=5
KPa N=1
KPb N=1
Cheng N=5
Group Name

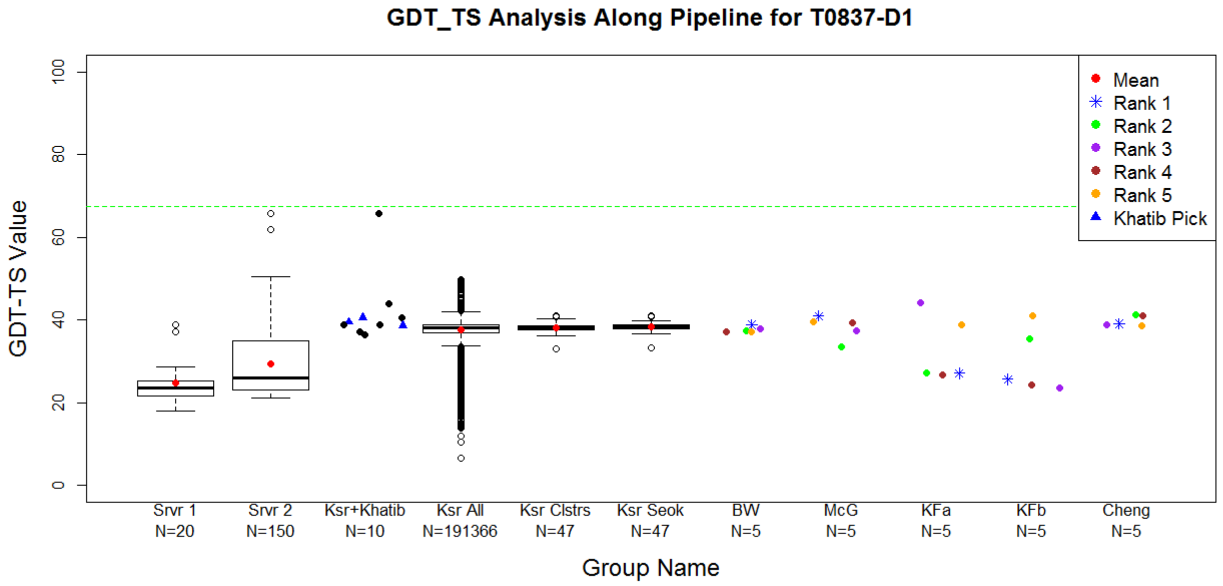
GDT_TS Analysis Along Pipeline for T0837-D1
GDT-TS Value
0
20
40
60
80
100
Mean
Rank 1
Rank 2
Rank 3
Rank 4
Rank 5
Khatib Pick
Srvr 1 N=20
Srvr 2 N=150
Ksr+Khatib N=10
Ksr All N=191366
Ksr Clstrs N=47
Ksr Seok N=47
BW N=5
McG N=5
KFa N=5
KFb N=5
Cheng N=5
Group Name

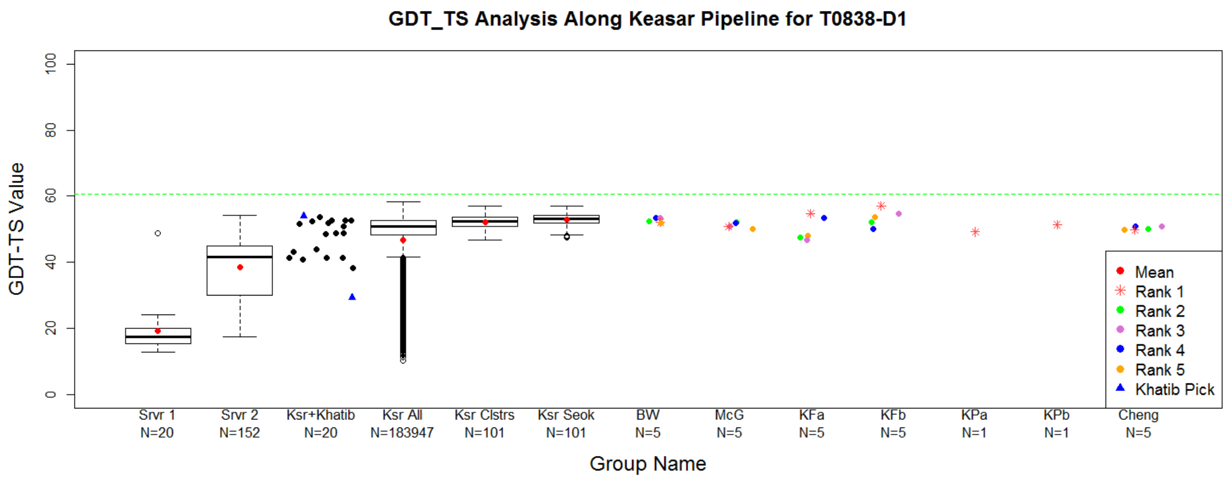
GDT_TS Analysis Along Keasar Pipeline for T0838-D1
GDT-TS Value
0
20
40
60
80
100
Srvr 1 N=20
Srvr 2 N=152
Ksr+Khatib N=20
Ksr All N=183947
Ksr Clstrs N=101
Ksr Seok N=101
BW N=5
McG N=5
KFa N=5
KFb N=5
KPa N=1
KPb N=1
Cheng N=5
Group Name
Mean
Rank 1
Rank 2
Rank 3
Rank 4
Rank 5
Khatib Pick

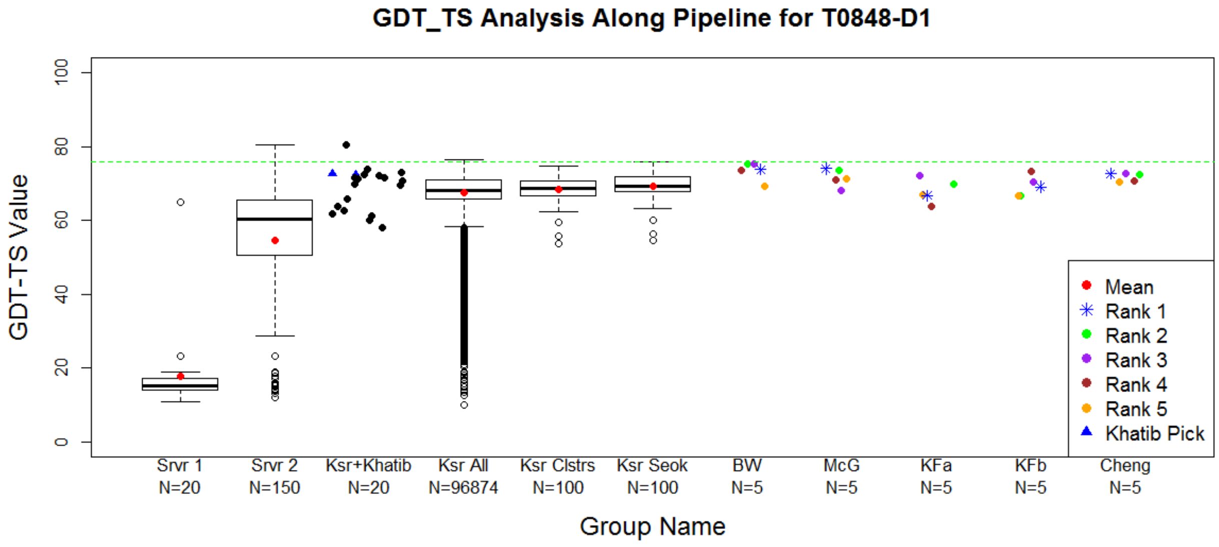
GDT_TS Analysis Along Pipeline for T0848-D1
GDT-TS Value
0
20
40
60
80
100
Srvr 1 N=20
Srvr 2 N=150
Ksr+Khatib N=20
Ksr All N=96874
Ksr Clstrs N=100
Ksr Seok N=100
BW N=5
McG N=5
KFa N=5
KFb N=5
Cheng N=5
Group Name
Mean
Rank 1
Rank 2
Rank 3
Rank 4
Rank 5
Khatib Pick

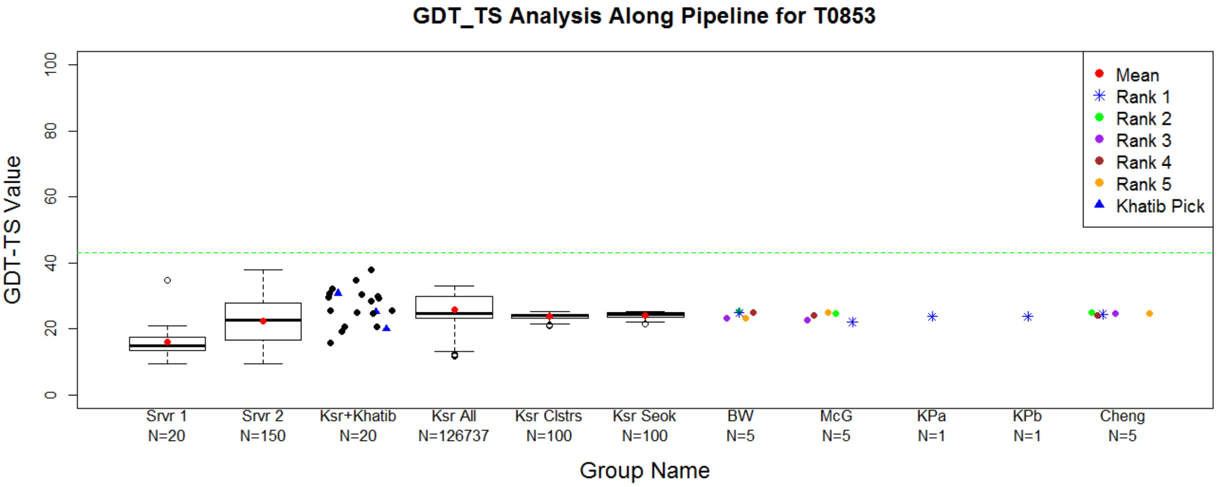


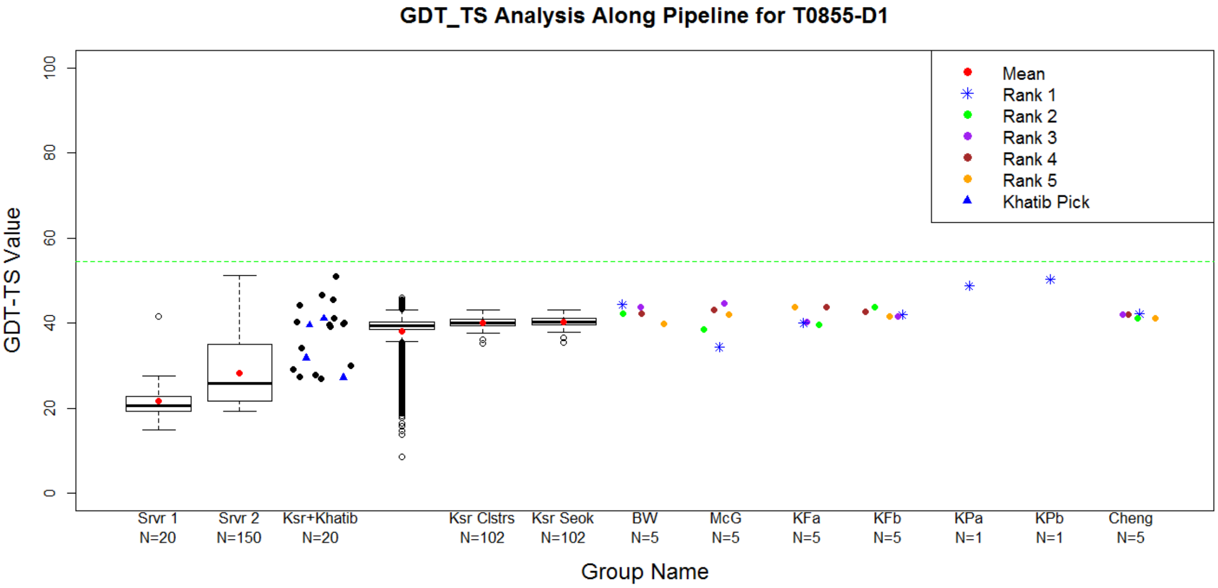


The following box and whiskers plots show each one of the steps in the Zhang pipelines. The first column shows a box and whiskers plot for the GDT_TS values of all the models created by Zhang's methods. The second column shows a box and whiskers plot for the cluster representatives calculated by Wallner's method and the third column shows the GDT_TS values of the same models refined by the GalaxyRefine method. The columns that follow show the selection by the different QA algorithms. The 5 models submitted by each group are marked in colors according to whether they were submitted as model 1, 2, 3, 4, or 5. Columns 4th and 10th show the selection by pipelines wfKsrFdit-BW-Sk-McG (which combined models from Keasar-Foldit and Zhang), wfZhng-Sk-BW, wfZhng-Ksr, wfMixKFa, wfMixKFb, wfMixKPa,

wfMixKPb, and wfAll-Cheng, respectively. The last five combined models from Keasar-Foldit, Zhang, and servers.

Zhang pipeline plots

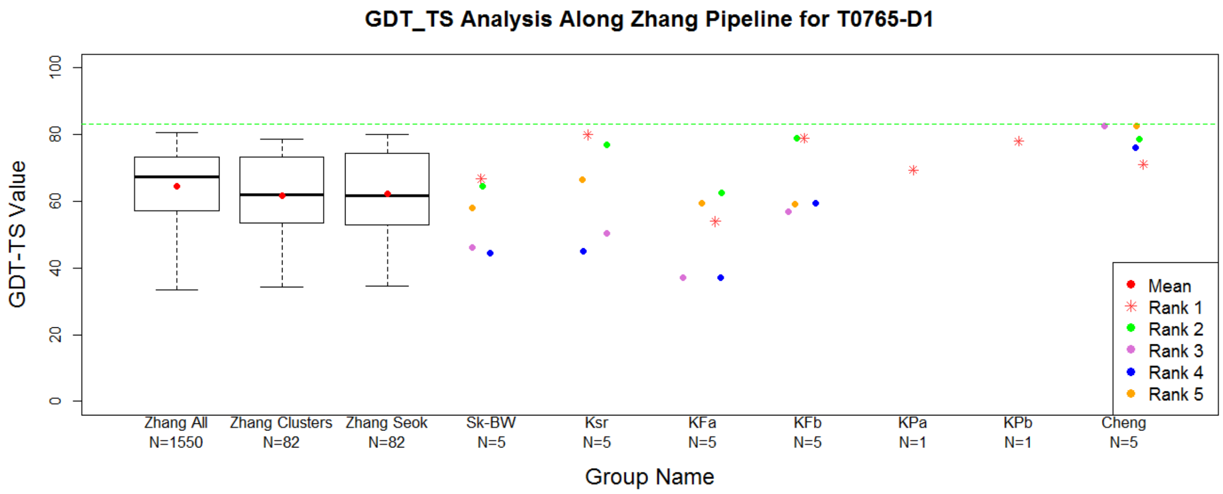


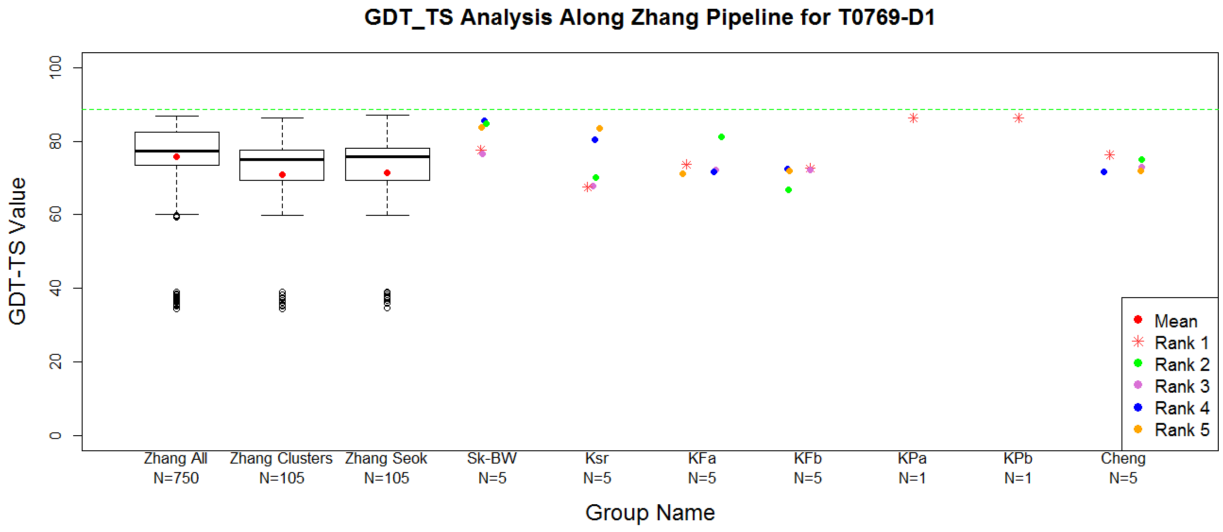

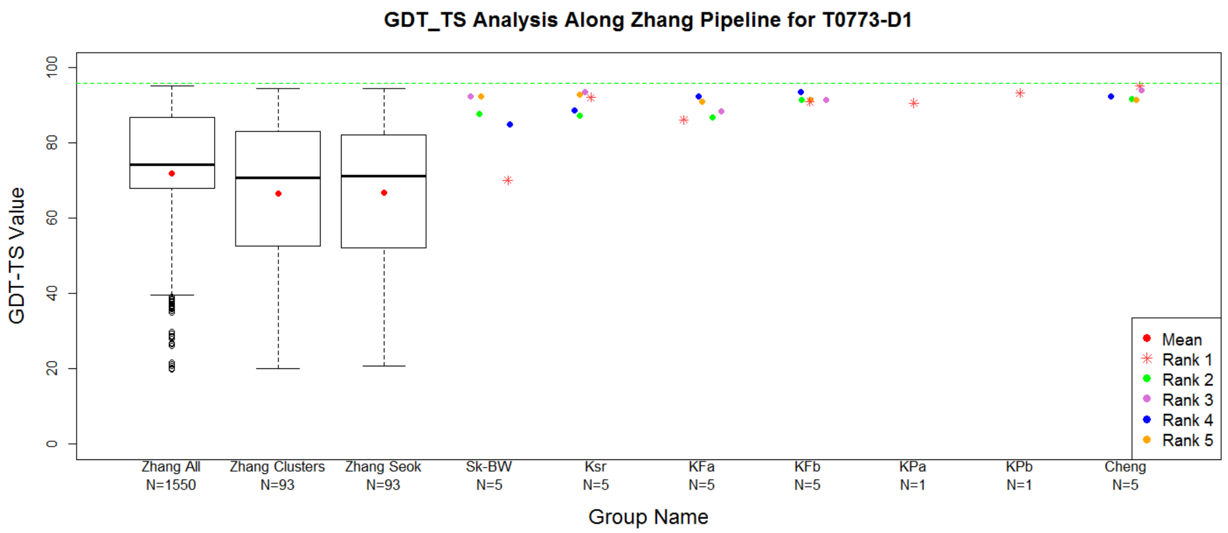
GDT_TS Analysis Along Zhang Pipeline for T0773-D1
GDT-TS Value
0
20
40
60
80
100
Mean
Rank 1
Rank 2
Rank 3
Rank 4
Rank 5
Zhang All N=1550
Zhang Clusters N=93
Zhang Seok N=93
Sk-BW N=5
Ksr N=5
KFa N=5
KFb N=5
KPa N=1
KPb N=1
Cheng N=5
Group Name

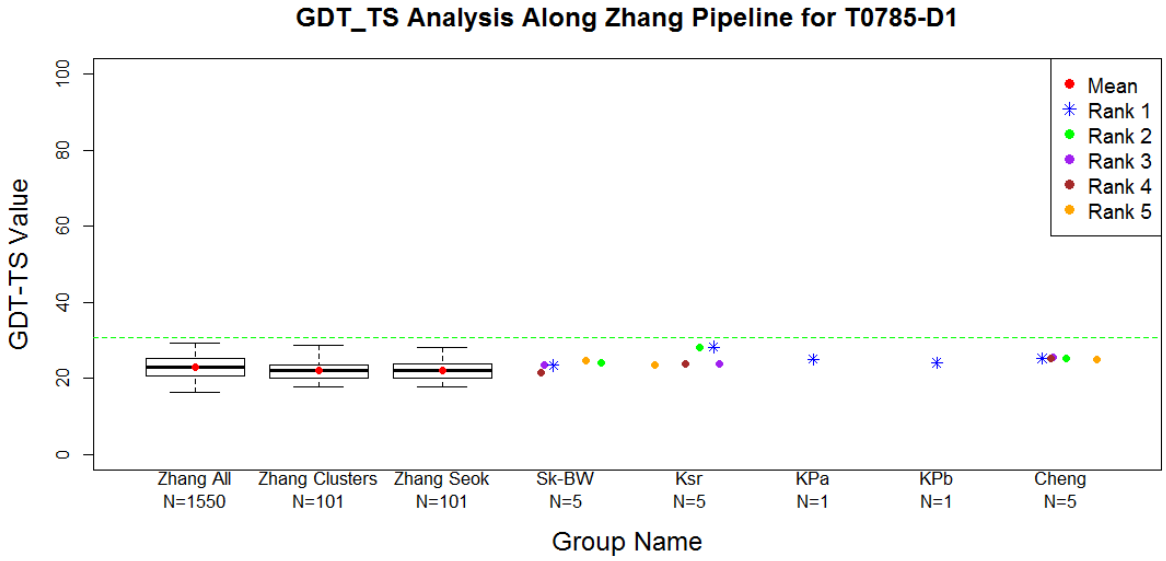
GDT_TS Analysis Along Zhang Pipeline for T0785-D1
GDT-TS Value
0
20
40
60
80
100
Mean
Rank 1
Rank 2
Rank 3
Rank 4
Rank 5
Zhang All N=1550
Zhang Clusters N=101
Zhang Seok N=101
Sk-BW N=5
Ksr N=5
KPa N=1
KPb N=1
Cheng N=5
Group Name

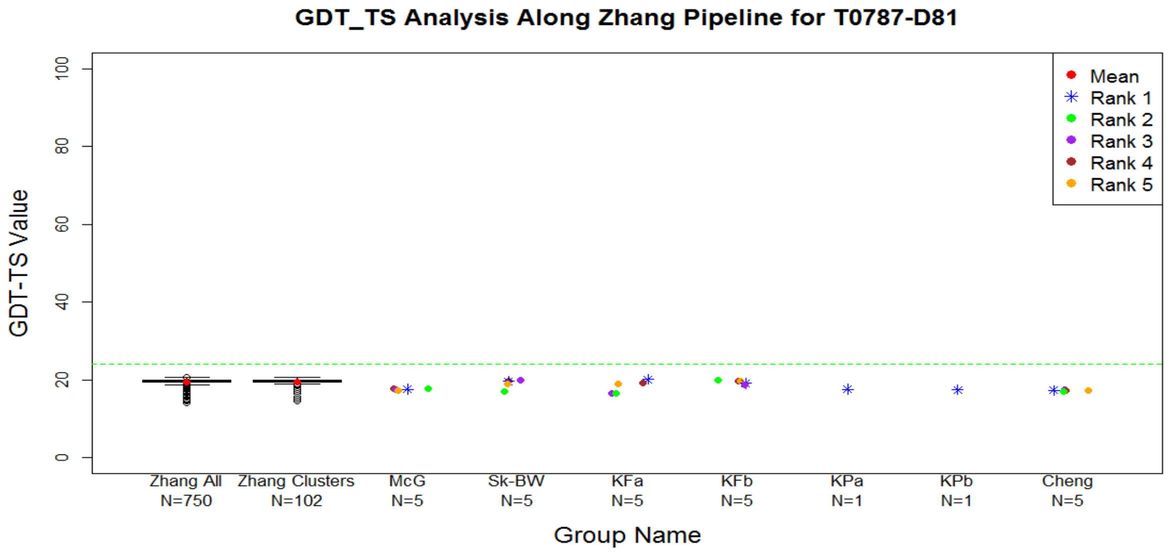

GDT_TS Analysis Along Zhang Pipeline for T0787-D81
GDT-TS Value
0
20
40
60
80
100
Mean
Rank 1
Rank 2
Rank 3
Rank 4
Rank 5
Zhang All N=750
Zhang Clusters N=102
McG N=5
Sk-BW N=5
KFa N=5
KFb N=5
KPa N=1
KPb N=1
Cheng N=5
Group Name


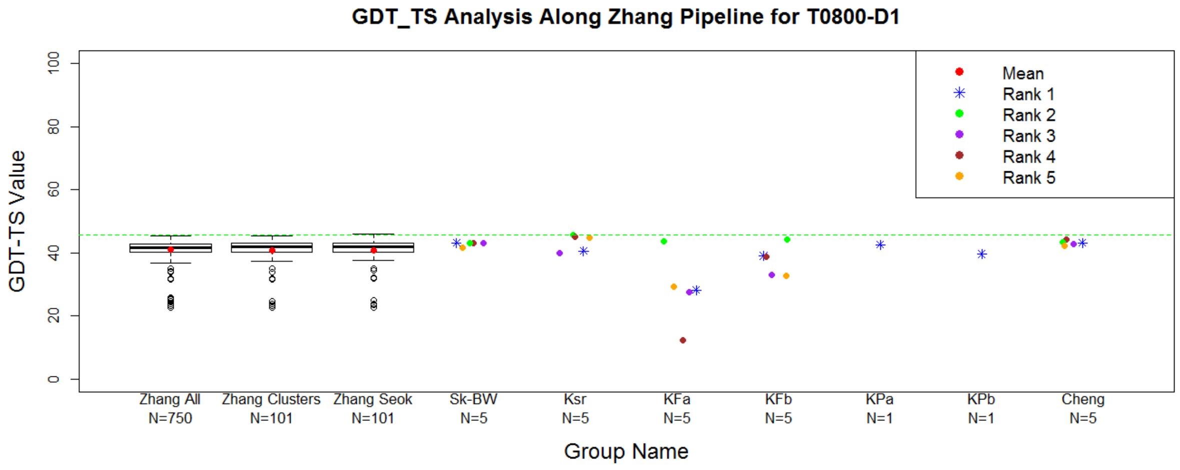

GDT_TS Analysis Along Zhang Pipeline for T0800-D1
GDT-TS Value
0
20
40
60
80
100
Mean
Rank 1
Rank 2
Rank 3
Rank 4
Rank 5
Zhang All N=750
Zhang Clusters N=101
Zhang Seok N=101
Sk-BW N=5
Ksr N=5
KFa N=5
KFb N=5
KPa N=1
KPb N=1
Cheng N=5
Group Name


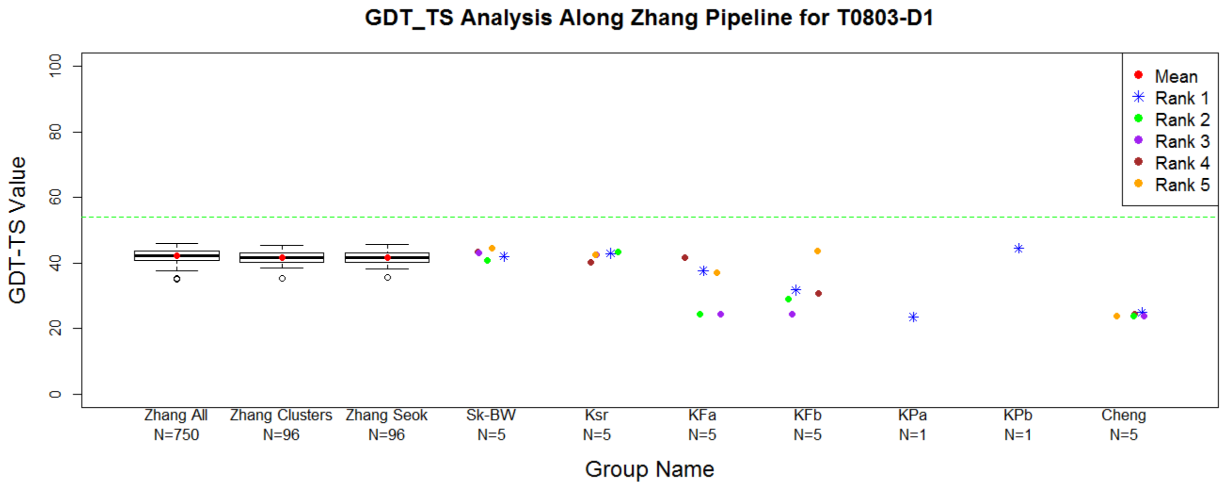

GDT_TS Analysis Along Zhang Pipeline for T0803-D1
GDT-TS Value
0
20
40
60
80
100
Mean
Rank 1
Rank 2
Rank 3
Rank 4
Rank 5
Zhang All N=750
Zhang Clusters N=96
Zhang Seok N=96
Sk-BW N=5
Ksr N=5
KFa N=5
KFb N=5
KPa N=1
KPb N=1
Cheng N=5
Group Name

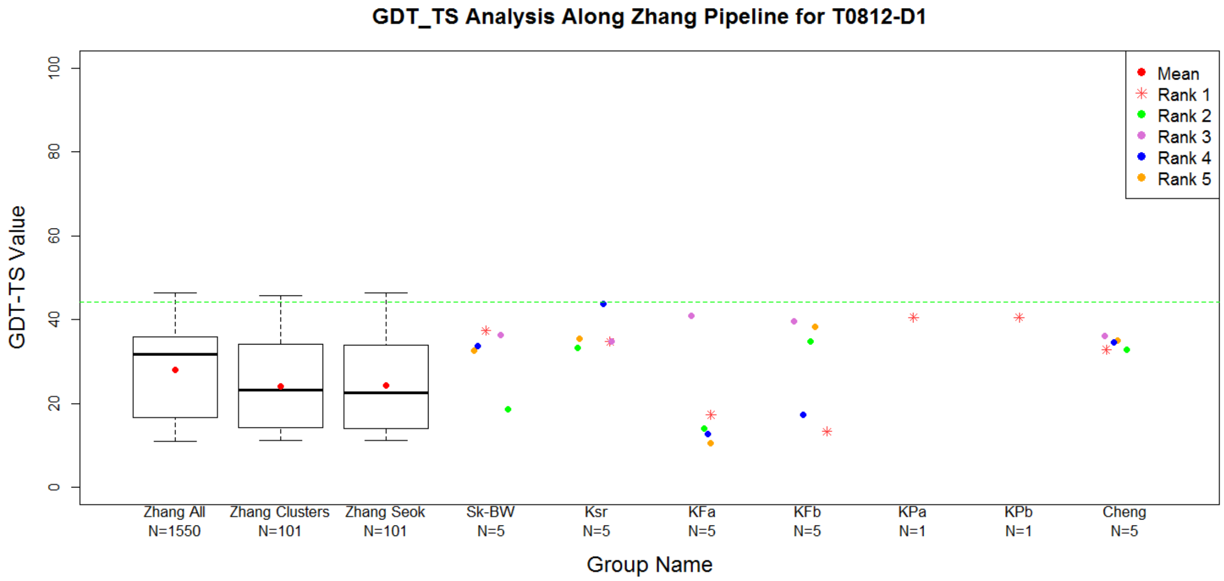
GDT_TS Analysis Along Zhang Pipeline for T0812-D1
GDT-TS Value
0
20
40
60
80
100
Mean
Rank 1
Rank 2
Rank 3
Rank 4
Rank 5
Zhang All N=1550
Zhang Clusters N=101
Zhang Seok N=101
Sk-BW N=5
Ksr N=5
KFa N=5
KFb N=5
KPa N=1
KPb N=1
Cheng N=5
Group Name

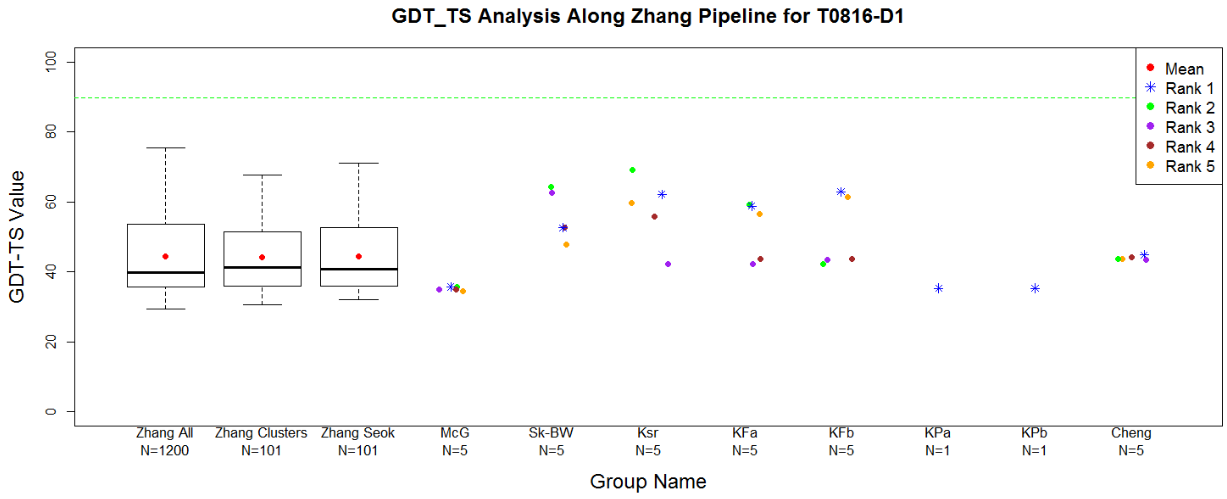
GDT_TS Analysis Along Zhang Pipeline for T0816-D1
GDT-TS Value
0
20
40
60
80
100
Mean
Rank 1
Rank 2
Rank 3
Rank 4
Rank 5
Zhang All N=1200
Zhang Clusters N=101
Zhang Seok N=101
McG N=5
Sk-BW N=5
Ksr N=5
KFa N=5
KFb N=5
KPa N=1
KPb N=1
Cheng N=5
Group Name

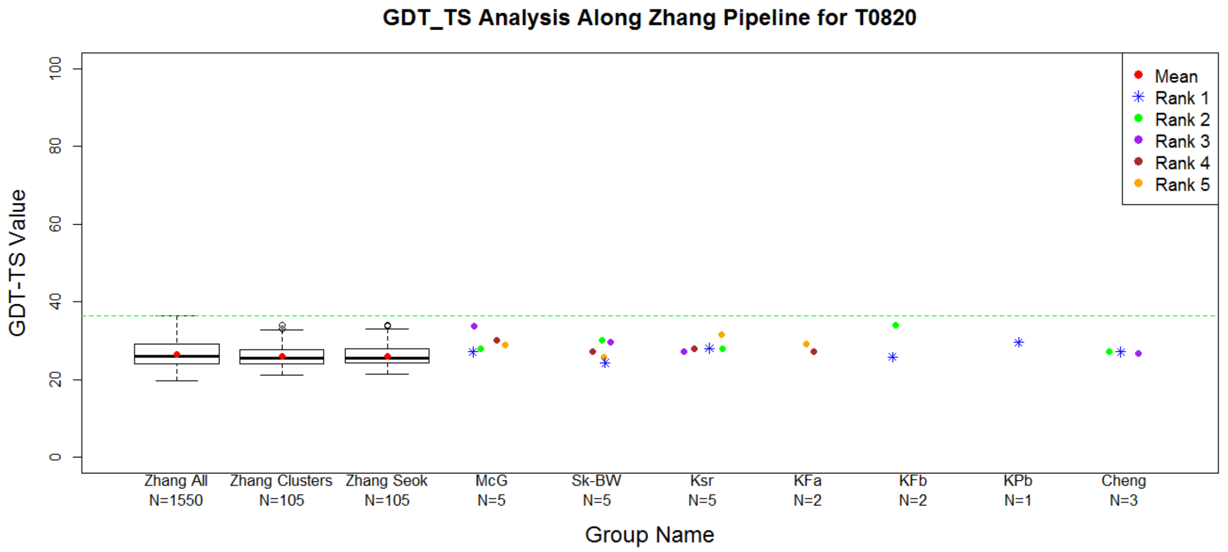
GDT_TS Analysis Along Zhang Pipeline for T0820
GDT-TS Value
0
20
40
60
80
100
Mean
Rank 1
Rank 2
Rank 3
Rank 4
Rank 5
Zhang All N=1550
Zhang Clusters N=105
Zhang Seok N=105
McG N=5
Sk-BW N=5
Ksr N=5
KFa N=2
KFb N=2
KPb N=1
Cheng N=3
Group Name

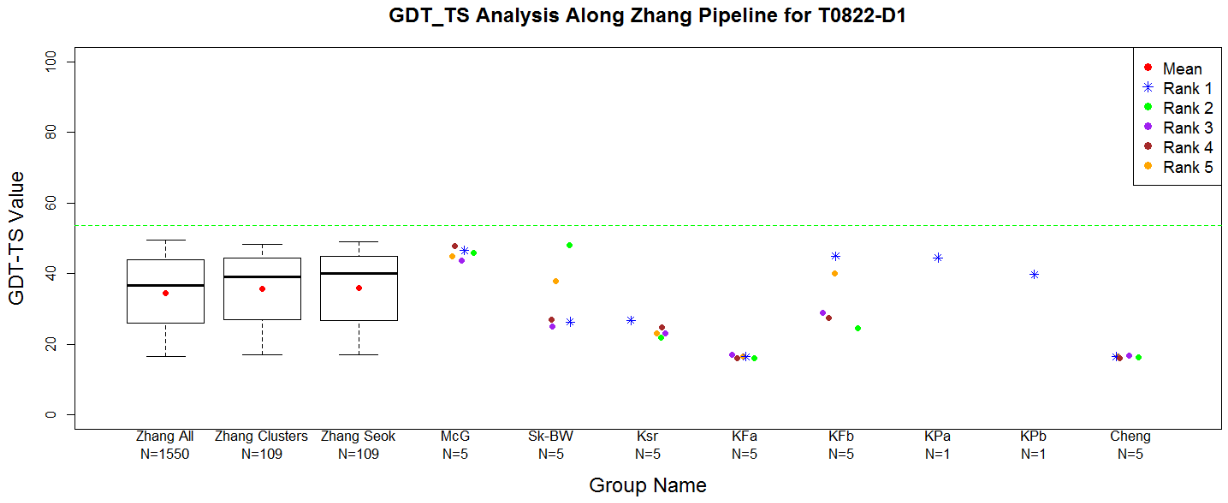
GDT_TS Analysis Along Zhang Pipeline for T0822-D1
GDT-TS Value
0
20
40
60
80
100
Mean
Rank 1
Rank 2
Rank 3
Rank 4
Rank 5
Zhang All N=1550
Zhang Clusters N=109
Zhang Seok N=109
McG N=5
Sk-BW N=5
Ksr N=5
KFa N=5
KFb N=5
KPa N=1
KPb N=1
Cheng N=5
Group Name

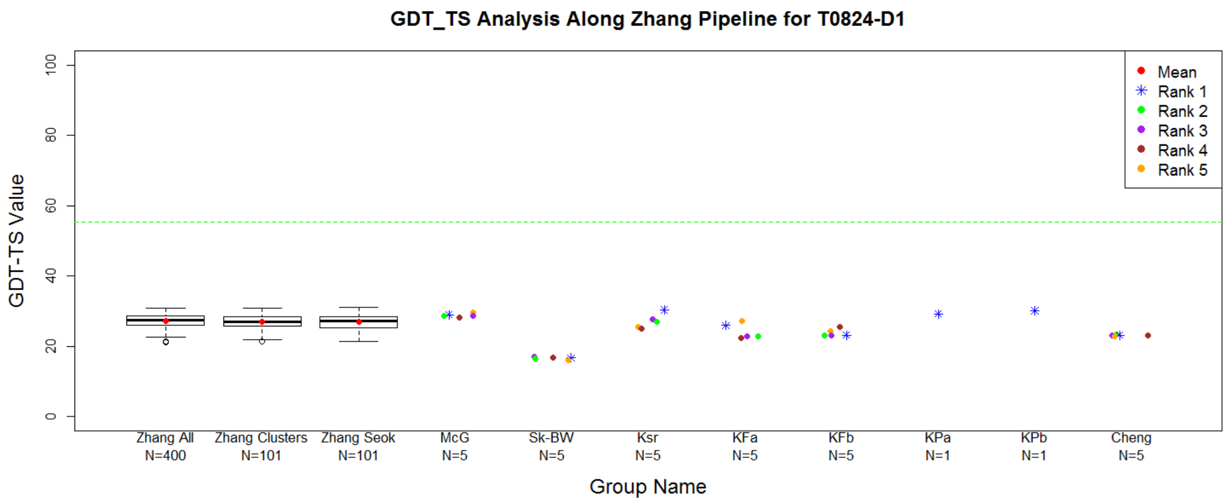

GDT_TS Analysis Along Zhang Pipeline for T0824-D1
GDT-TS Value
0
20
40
60
80
100
Mean
Rank 1
Rank 2
Rank 3
Rank 4
Rank 5
Zhang All
N=400
Zhang Clusters
N=101
Zhang Seok
N=101
McG
N=5
Sk-BW
N=5
Ksr
N=5
KFa
N=5
KFb
N=5
KPa
N=1
KPb
N=1
Cheng
N=5
Group Name


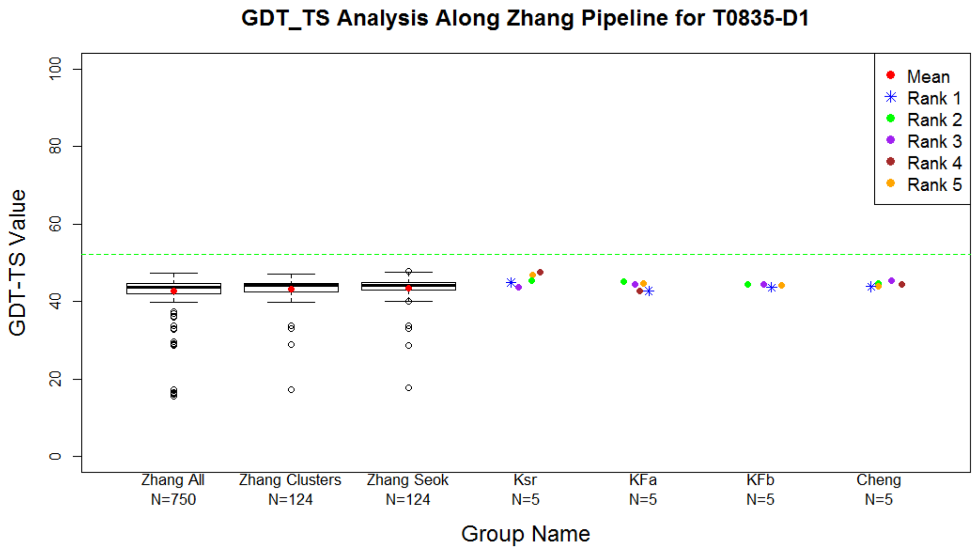

GDT_TS Analysis Along Zhang Pipeline for T0835-D1
GDT-TS Value
0
20
40
60
80
100
Mean
Rank 1
Rank 2
Rank 3
Rank 4
Rank 5
Zhang All
N=750
Zhang Clusters
N=124
Zhang Seok
N=124
Ksr
N=5
KFa
N=5
KFb
N=5
Cheng
N=5
Group Name

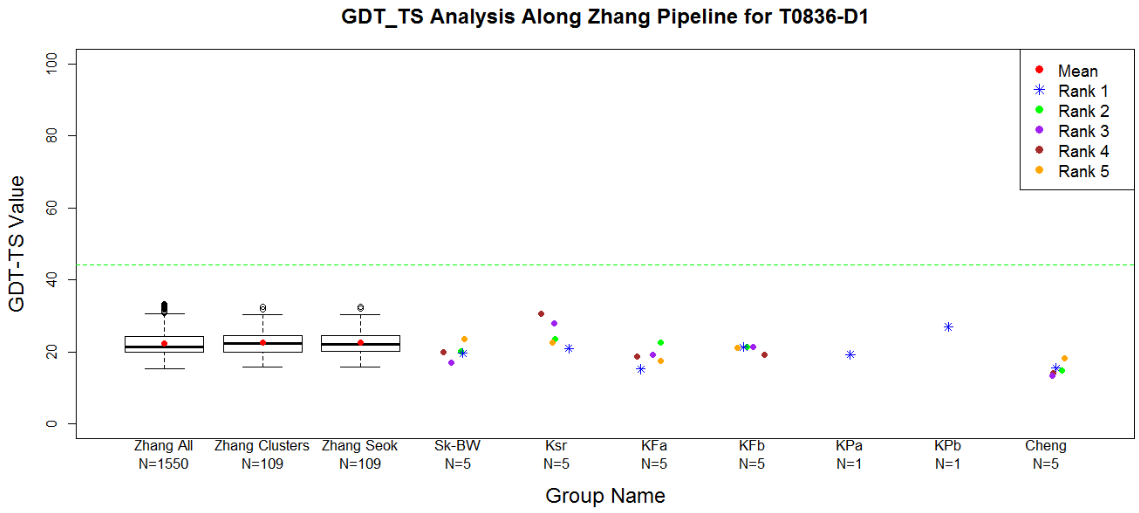
GDT_TS Analysis Along Zhang Pipeline for T0836-D1
GDT-TS Value
0
20
40
60
80
100
Mean
Rank 1
Rank 2
Rank 3
Rank 4
Rank 5
Zhang All
N=1550
Zhang Clusters
N=109
Zhang Seok
N=109
Sk-BW
N=5
Ksr
N=5
KFa
N=5
KFb
N=5
KPa
N=1
KPb
N=1
Cheng
N=5
Group Name

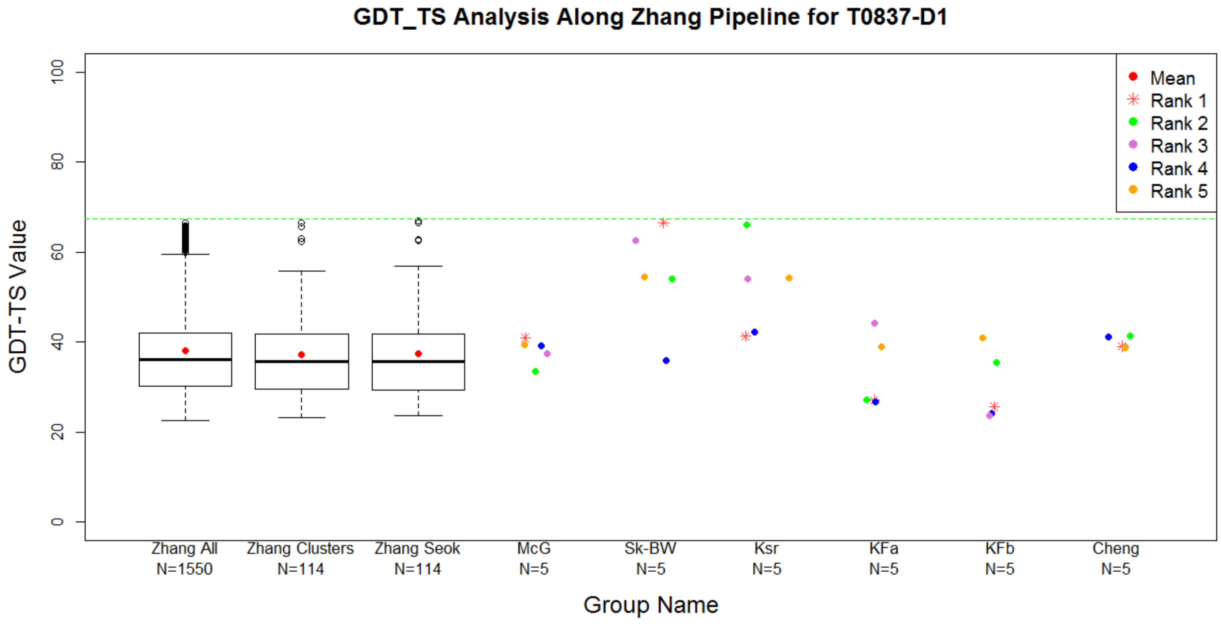
GDT_TS Analysis Along Zhang Pipeline for T0837-D1
GDT-TS Value
0
20
40
60
80
100
Mean
Rank 1
Rank 2
Rank 3
Rank 4
Rank 5
Zhang All
N=1550
Zhang Clusters
N=114
Zhang Seok
N=114
McG
N=5
Sk-BW
N=5
Ksr
N=5
KFa
N=5
KFb
N=5
Cheng
N=5
Group Name

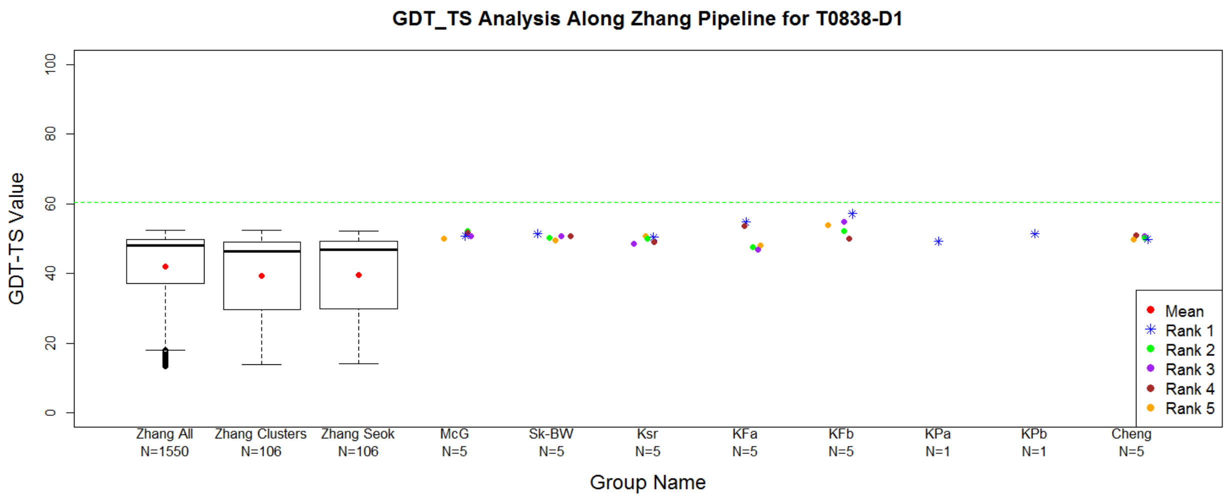


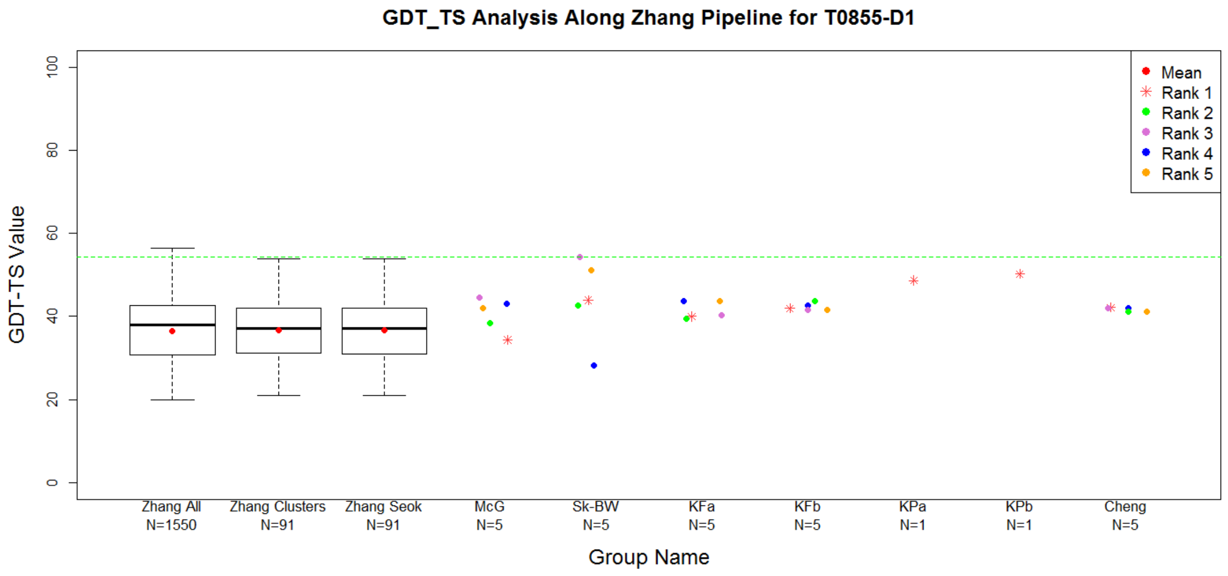


The following box and whiskers plots compare the GDT_TS of the initial set of Keasar-Foldit, Zhang, and CPUNK models from which the different WeFold groups selected 5 models. Columns 1-3 show box and whiskers plots for the entire set of Foldit models, clusters, and refined clusters. Columns 4-6 show plots for entire set of Zhang models, clusters, and refined clusters. Finally, column 7 shows the entire set of models generated by UNRES for the CPUNK pipeline (results fro wfCPUNK are not available for all the targets considered in this analysis). The dashed green line represents the GDT_TS value of the best model submitted to CASP11 for that target considering all the CASP11 groups.

Group Comparison Plots

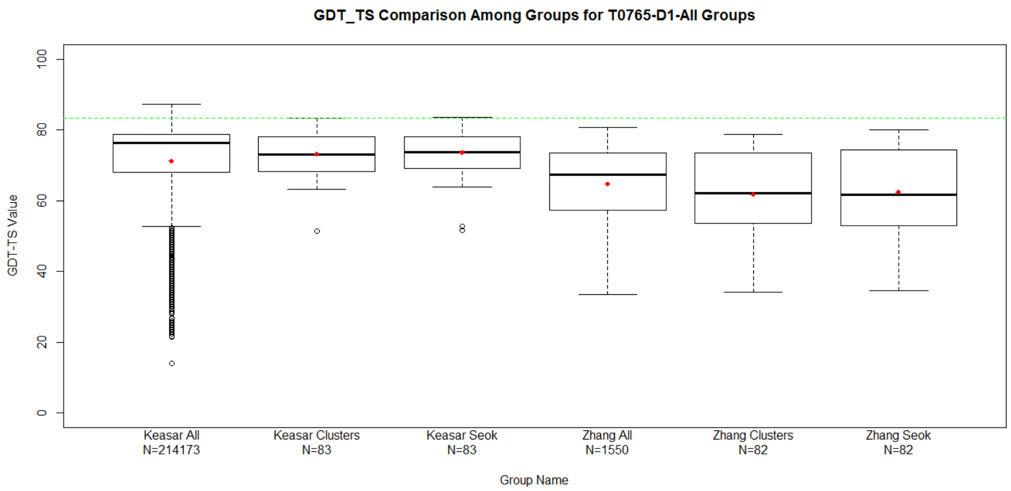
GDT_TS Comparison Among Groups for T0765-D1-All Groups
GDT-TS Value
0
20
40
60
80
100
Keasar All
N=214173
Keasar Clusters
N=83
Keasar Seok
N=83
Zhang All
N=1550
Zhang Clusters
N=82
Zhang Seok
N=82
Group Name

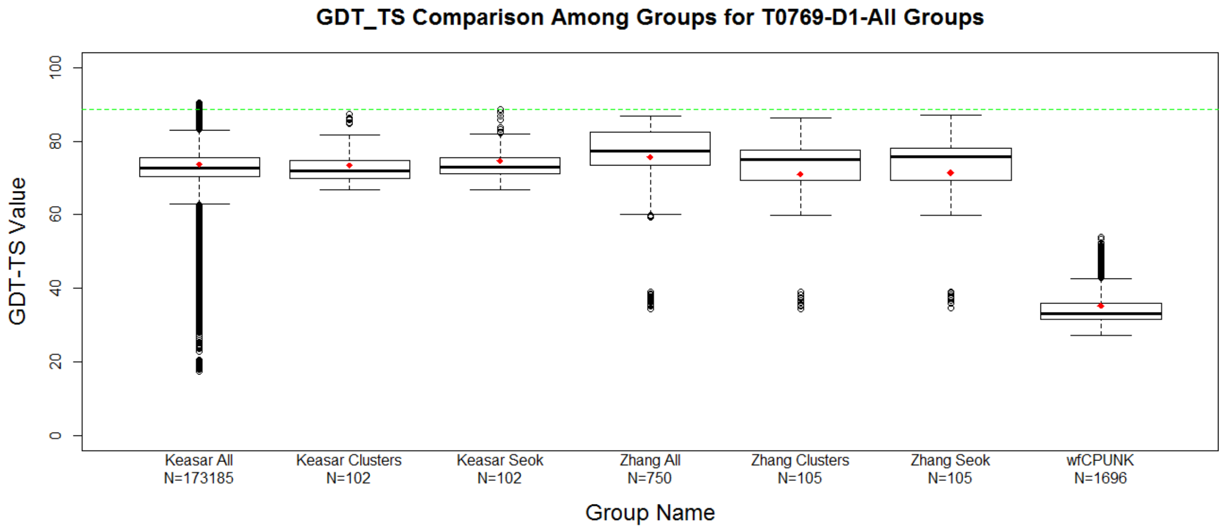
GDT_TS Comparison Among Groups for T0769-D1-All Groups
GDT-TS Value
0
20
40
60
80
100
Keasar All
N=173185
Keasar Clusters
N=102
Keasar Seok
N=102
Zhang All
N=750
Zhang Clusters
N=105
Zhang Seok
N=105
wfCPUNK
N=1696
Group Name

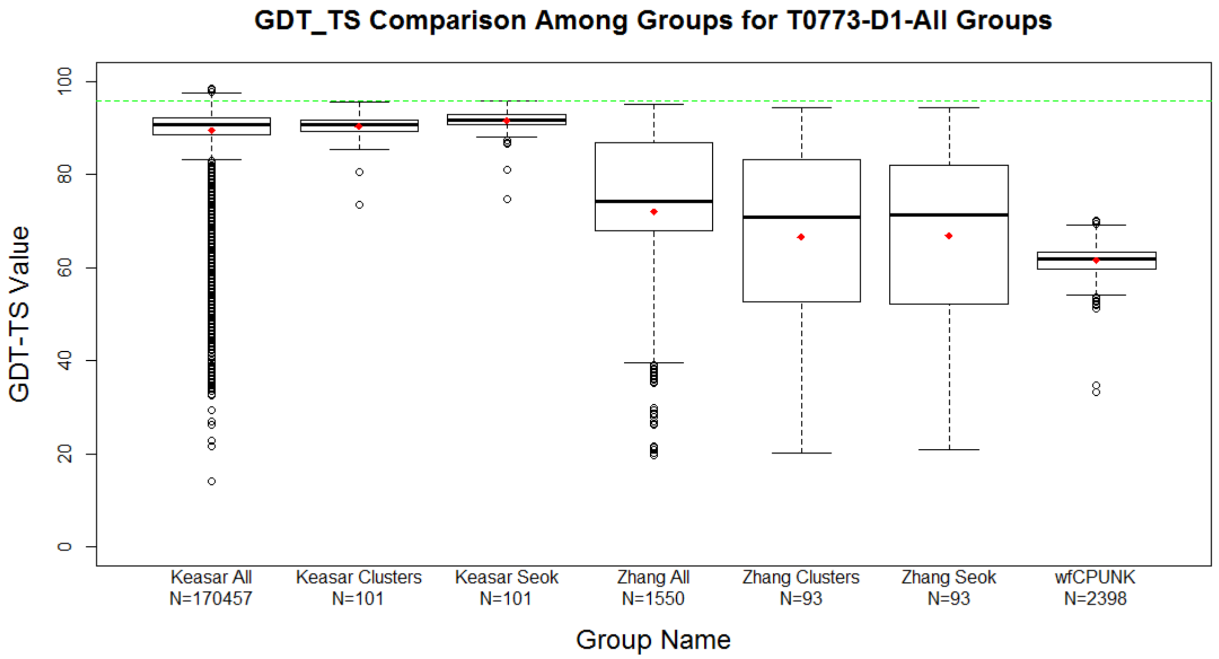
GDT_TS Comparison Among Groups for T0773-D1-All Groups
GDT-TS Value
0
20
40
60
80
100
Keasar All
N=170457
Keasar Clusters
N=101
Keasar Seok
N=101
Zhang All
N=1550
Zhang Clusters
N=93
Zhang Seok
N=93
wfCPUNK
N=2398
Group Name

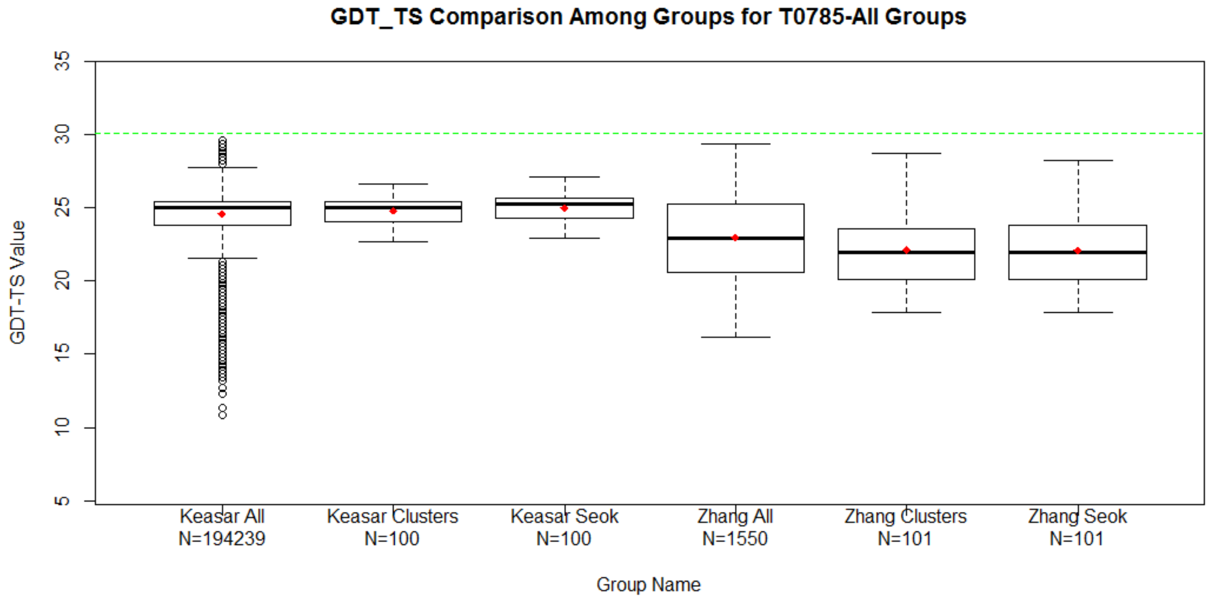
GDT_TS Comparison Among Groups for T0785-All Groups
GDT-TS Value
5
10
15
20
25
30
35
Keasar All
N=194239
Keasar Clusters
N=100
Keasar Seok
N=100
Zhang All
N=1550
Zhang Clusters
N=101
Zhang Seok
N=101
Group Name

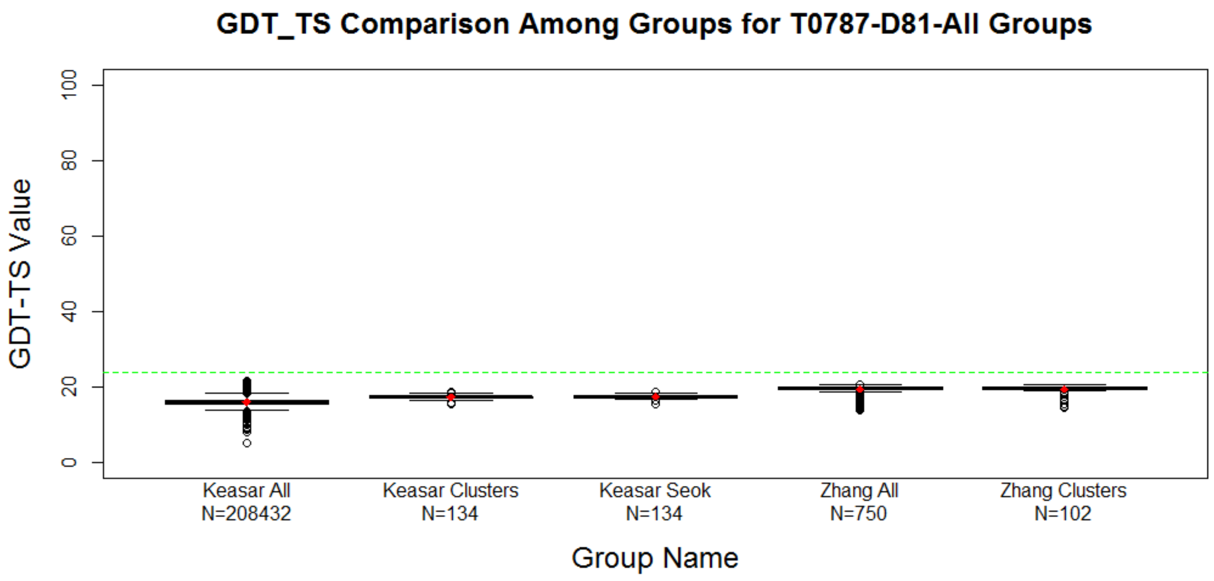
GDT_TS Comparison Among Groups for T0787-D81-All Groups
GDT-TS Value
0
20
40
60
80
100
Keasar All
N=208432
Keasar Clusters
N=134
Keasar Seok
N=134
Zhang All
N=750
Zhang Clusters
N=102
Group Name

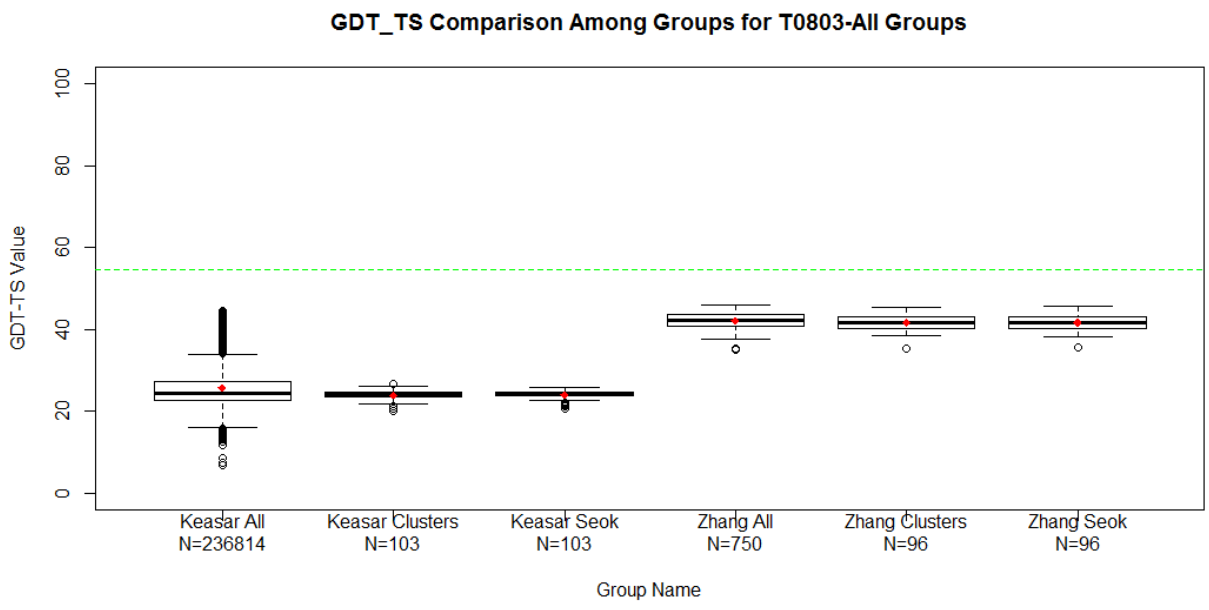
GDT_TS Comparison Among Groups for T0803-All Groups
GDT-TS Value
0
20
40
60
80
100
Keasar All
N=236814
Keasar Clusters
N=103
Keasar Seok
N=103
Zhang All
N=750
Zhang Clusters
N=96
Zhang Seok
N=96
Group Name

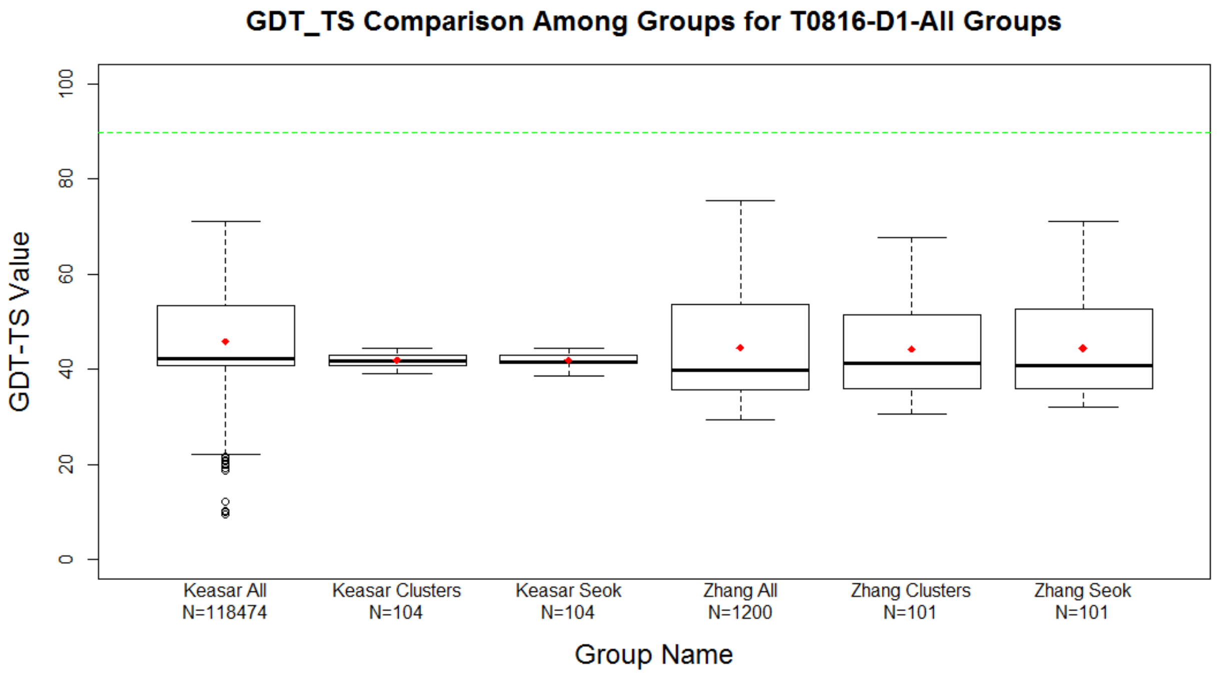
GDT_TS Comparison Among Groups for T0816-D1-All Groups
GDT-TS Value
0
20
40
60
80
100
Keasar All
N=118474
Keasar Clusters
N=104
Keasar Seok
N=104
Zhang All
N=1200
Zhang Clusters
N=101
Zhang Seok
N=101
Group Name

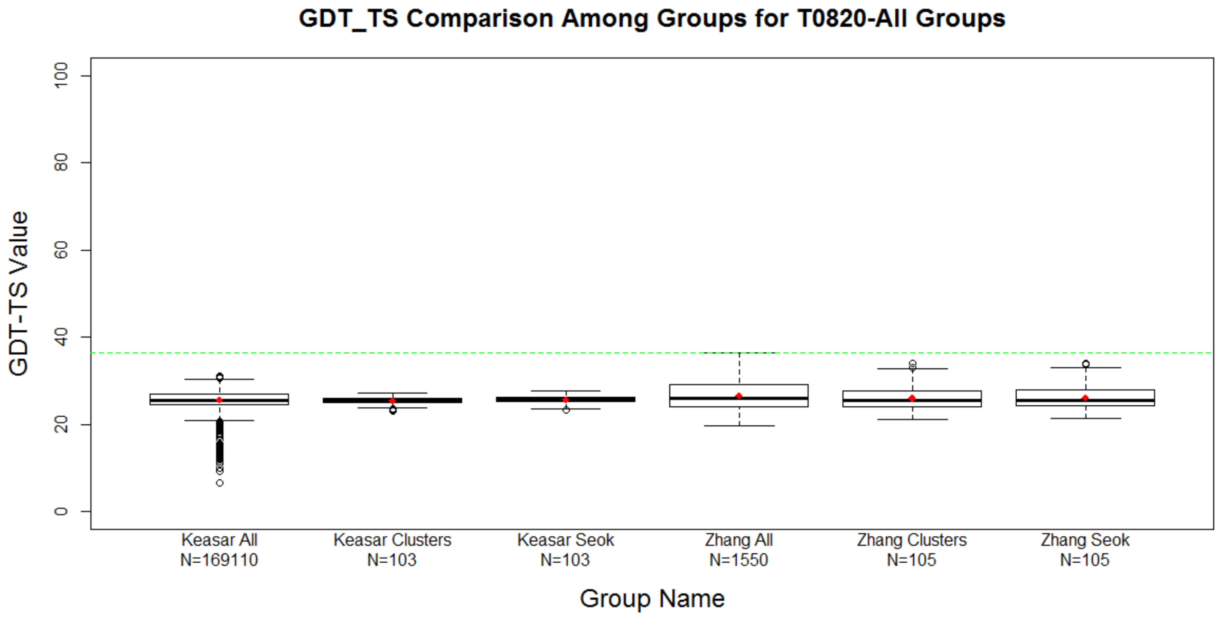
GDT_TS Comparison Among Groups for T0820-All Groups
GDT-TS Value
0
20
40
60
80
100
Keasar All
N=169110
Keasar Clusters
N=103
Keasar Seok
N=103
Zhang All
N=1550
Zhang Clusters
N=105
Zhang Seok
N=105
Group Name

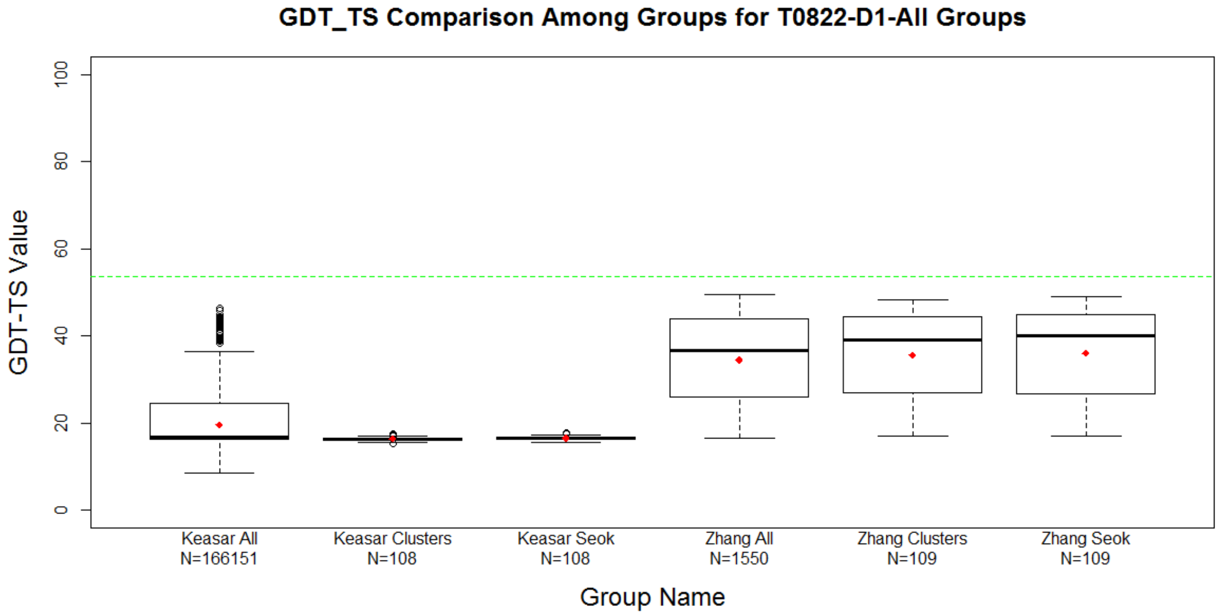
GDT_TS Comparison Among Groups for T0822-D1-All Groups
GDT-TS Value
0
20
40
60
80
100
Keasar All
N=166151
Keasar Clusters
N=108
Keasar Seok
N=108
Zhang All
N=1550
Zhang Clusters
N=109
Zhang Seok
N=109
Group Name

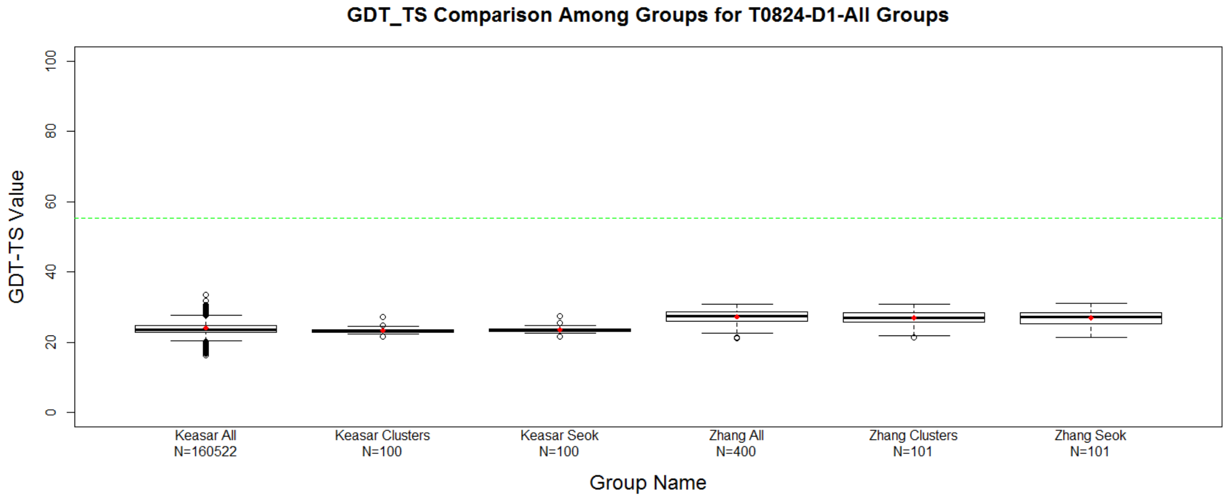
GDT_TS Comparison Among Groups for T0824-D1-All Groups
GDT-TS Value
0
20
40
60
80
100
Keasar All
N=160522
Keasar Clusters
N=100
Keasar Seok
N=100
Zhang All
N=400
Zhang Clusters
N=101
Zhang Seok
N=101
Group Name

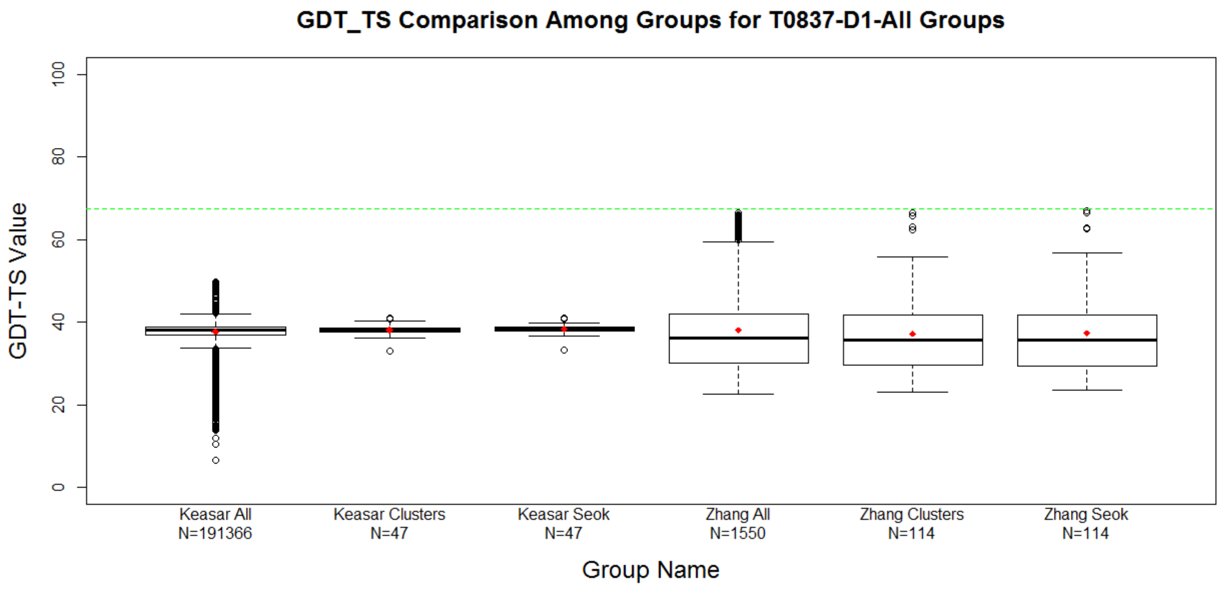

GDT_TS Comparison Among Groups for T0837-D1-All Groups
GDT-TS Value
0
20
40
60
80
100
Keasar All N=191366
Keasar Clusters N=47
Keasar Seok N=47
Zhang All N=1550
Zhang Clusters N=114
Zhang Seok N=114
Group Name


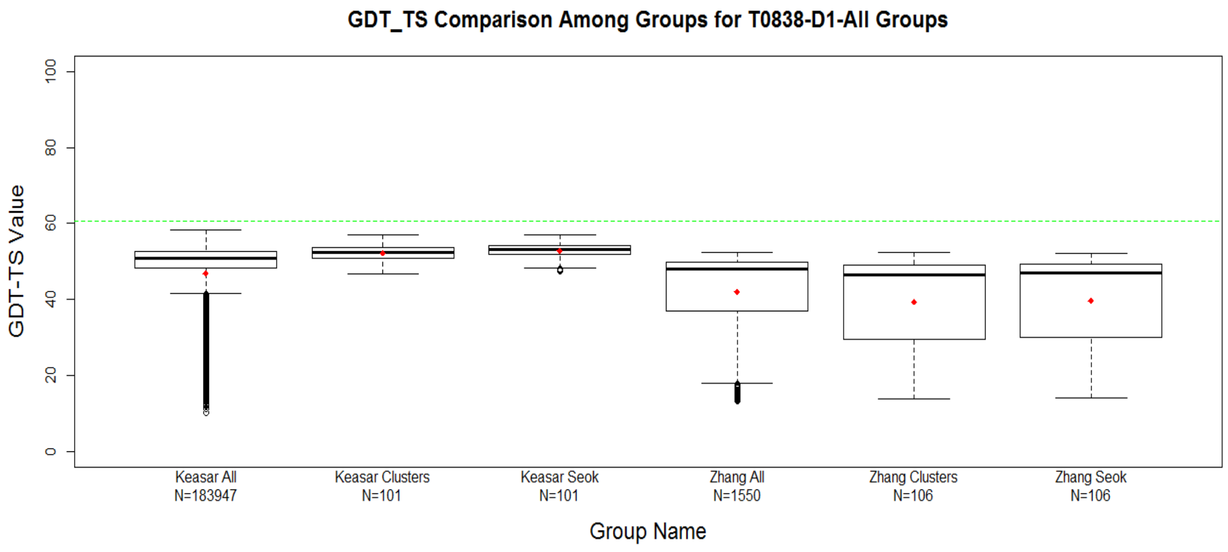

GDT_TS Comparison Among Groups for T0838-D1-All Groups
GDT-TS Value
0
20
40
60
80
100
Keasar All N=183947
Keasar Clusters N=101
Keasar Seok N=101
Zhang All N=1550
Zhang Clusters N=106
Zhang Seok N=106
Group Name

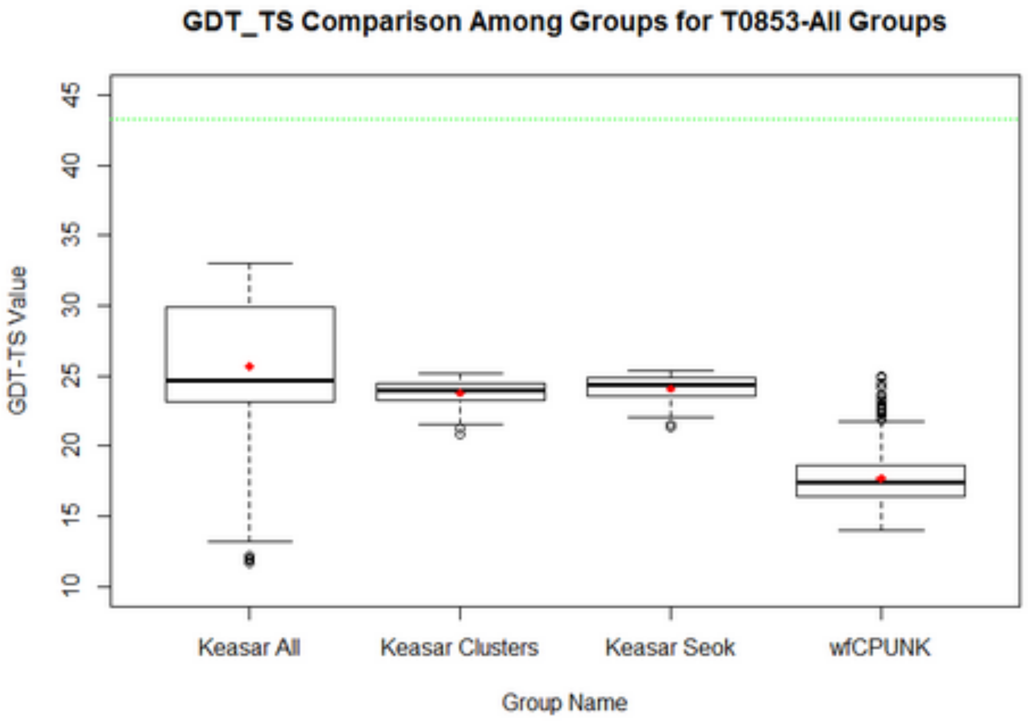


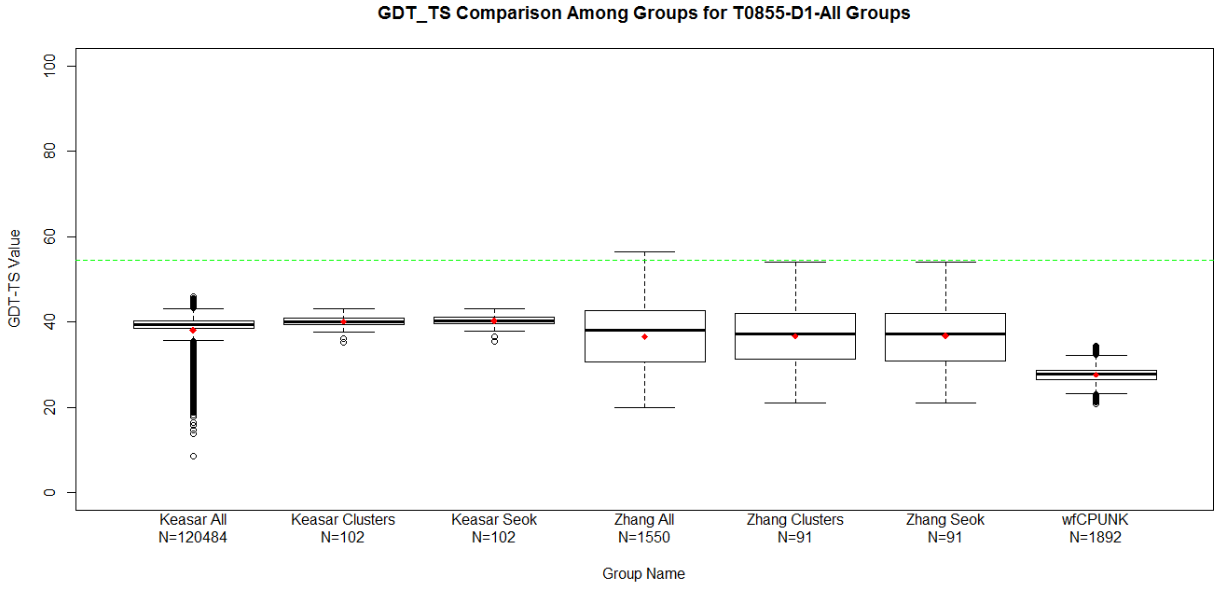


The following box and whiskers plots show each one of the steps in the refinement Foldit pipelines. Each plot corresponds to one of the refinement targets submitted to CASP11 by these pipelines. The first column shows the hundreds of thousands of models generated by Foldit players when given an initial model whose GDT_HA value is represented by the dashed red line.

The GDT_HA value of the best models submitted is marked with a dashed green line, which is labeled with the name of the CASP11 team that submitted the best model. The second column shows a box and whiskers plot for the cluster representatives calculated by Wallner's method and the third column shows the GDT_HA values of the same models refined by KoBaMIN [82]. These GDT_HA values of the clusters are slightly improved by the refinement algorithm as it can be seen in the plots. The columns that follow show the selection by the different algorithms. The 5 models submitted by each group are marked in colors according to whether they were submitted as model 1, 2, 3, 4, or 5. Columns 4th and 5$^{th}$ columns show the selection by the SVLab group from the clusters (pipeline *wfFdit_BW_SVGroup*) and from the refined clusters (pipeline *wfFdit_BW_K_SVGroup*). The 5$^{th}$ and 6$^{th}$ columns show the selection by ModFOLD5_single (pipeline *wfFdit-K-McG)* and by ProQ2 (pipeline *wfFdit-BW-KB-B*W).

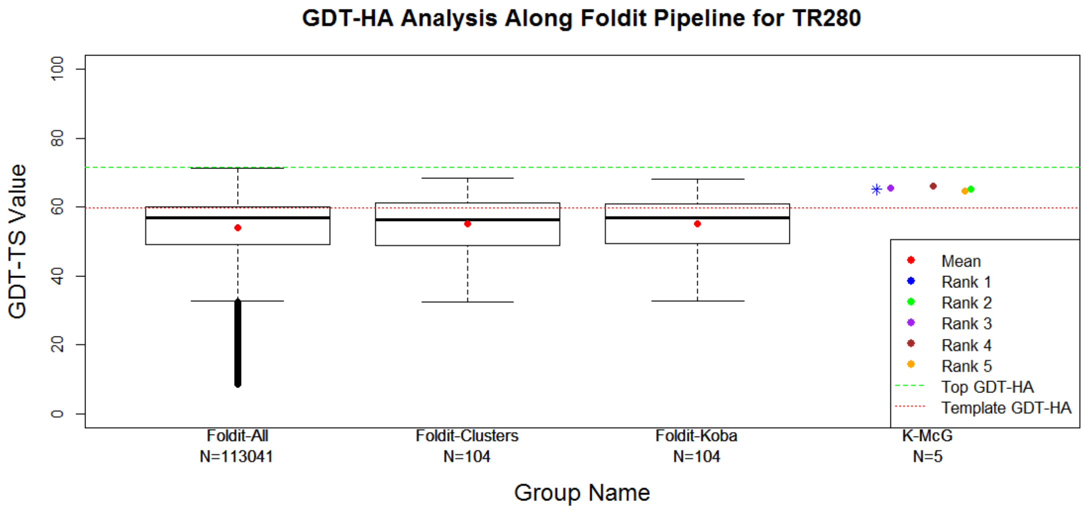


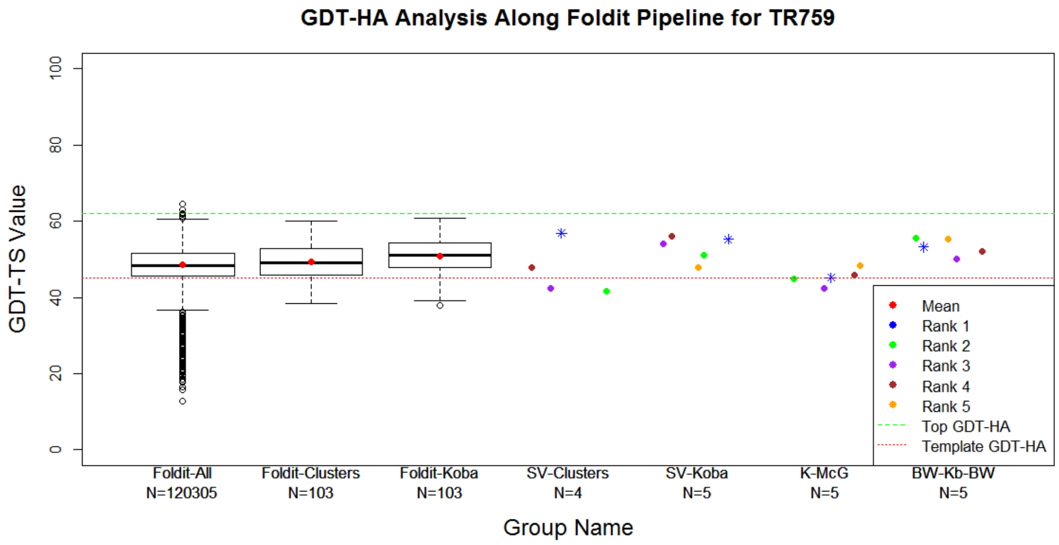

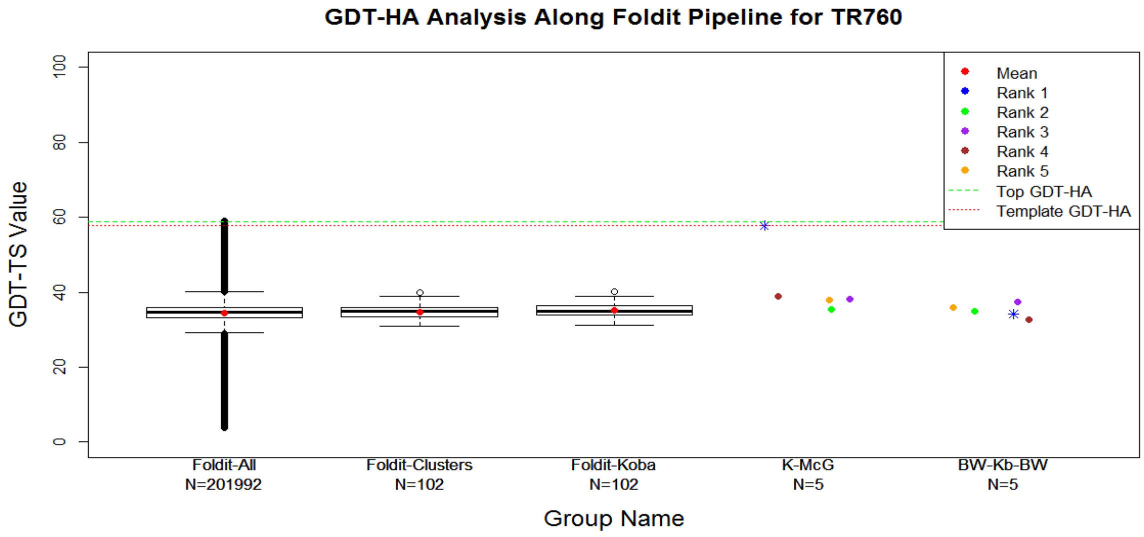
GDT-HA Analysis Along Foldit Pipeline for TR760
GDT-TS Value
Group Name
Foldit-All N=201992
Foldit-Clusters N=102
Foldit-Koba N=102
K-McG N=5
BW-Kb-BW N=5
Mean
Rank 1
Rank 2
Rank 3
Rank 4
Rank 5
Top GDT-HA
Template GDT-HA
0
20
40
60
80
100

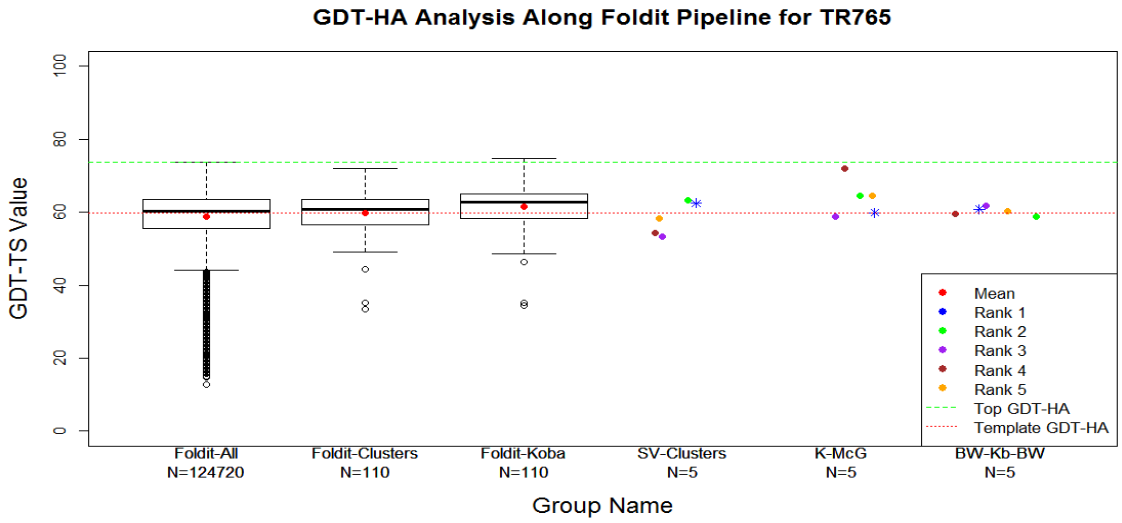
GDT-HA Analysis Along Foldit Pipeline for TR765
GDT-TS Value
Group Name
Foldit-All N=124720
Foldit-Clusters N=110
Foldit-Koba N=110
SV-Clusters N=5
K-McG N=5
BW-Kb-BW N=5
Mean
Rank 1
Rank 2
Rank 3
Rank 4
Rank 5
Top GDT-HA
Template GDT-HA
0
20
40
60
80
100

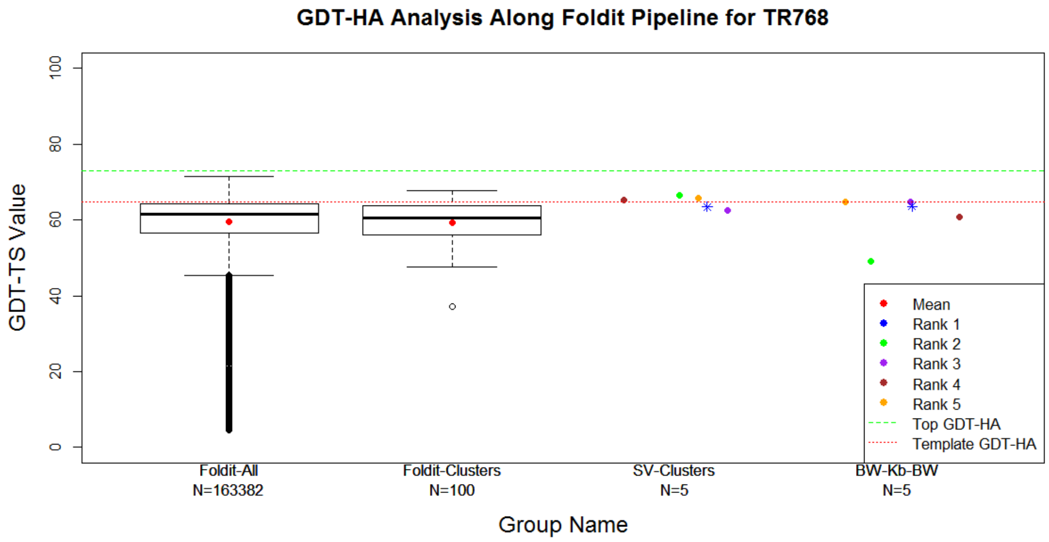
GDT-HA Analysis Along Foldit Pipeline for TR768
GDT-TS Value
0
20
40
60
80
100
Mean
Rank 1
Rank 2
Rank 3
Rank 4
Rank 5
Top GDT-HA
Template GDT-HA
Foldit-All
N=163382
Foldit-Clusters
N=100
SV-Clusters
N=5
BW-Kb-BW
N=5
Group Name

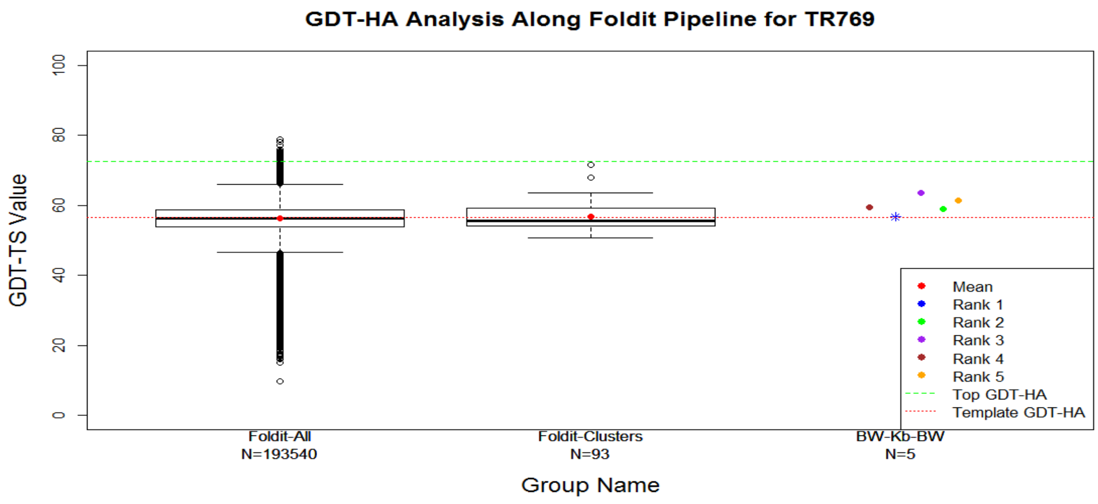
GDT-HA Analysis Along Foldit Pipeline for TR769
GDT-TS Value
0
20
40
60
80
100
Mean
Rank 1
Rank 2
Rank 3
Rank 4
Rank 5
Top GDT-HA
Template GDT-HA
Foldit-All
N=193540
Foldit-Clusters
N=93
BW-Kb-BW
N=5
Group Name

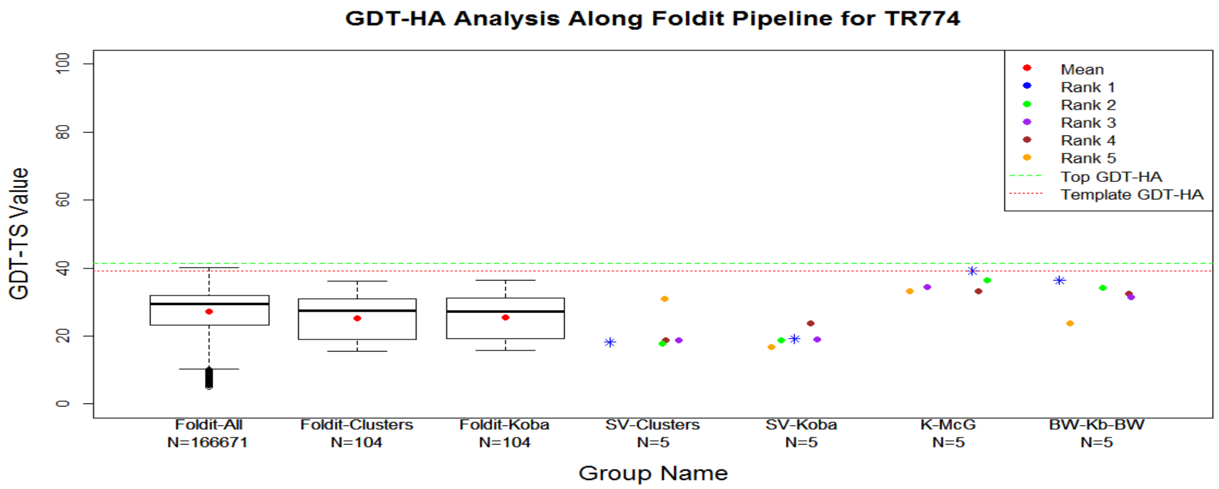
GDT-HA Analysis Along Foldit Pipeline for TR774
GDT-TS Value
Mean
Rank 1
Rank 2
Rank 3
Rank 4
Rank 5
Top GDT-HA
Template GDT-HA
Foldit-All N=166671
Foldit-Clusters N=104
Foldit-Koba N=104
SV-Clusters N=5
SV-Koba N=5
K-McG N=5
BW-Kb-BW N=5
Group Name

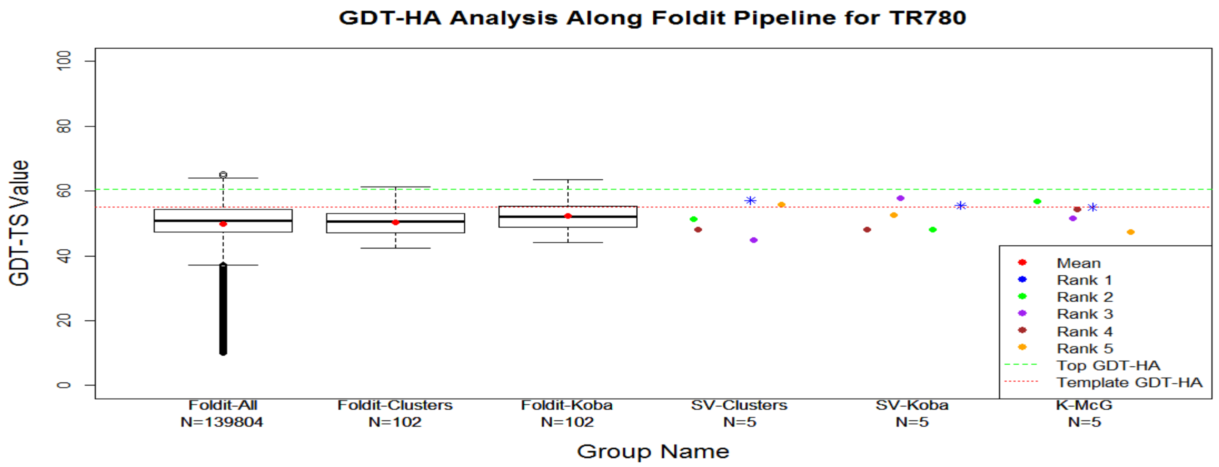
GDT-HA Analysis Along Foldit Pipeline for TR780
GDT-TS Value
Mean
Rank 1
Rank 2
Rank 3
Rank 4
Rank 5
Top GDT-HA
Template GDT-HA
Foldit-All N=139804
Foldit-Clusters N=102
Foldit-Koba N=102
SV-Clusters N=5
SV-Koba N=5
K-McG N=5
Group Name

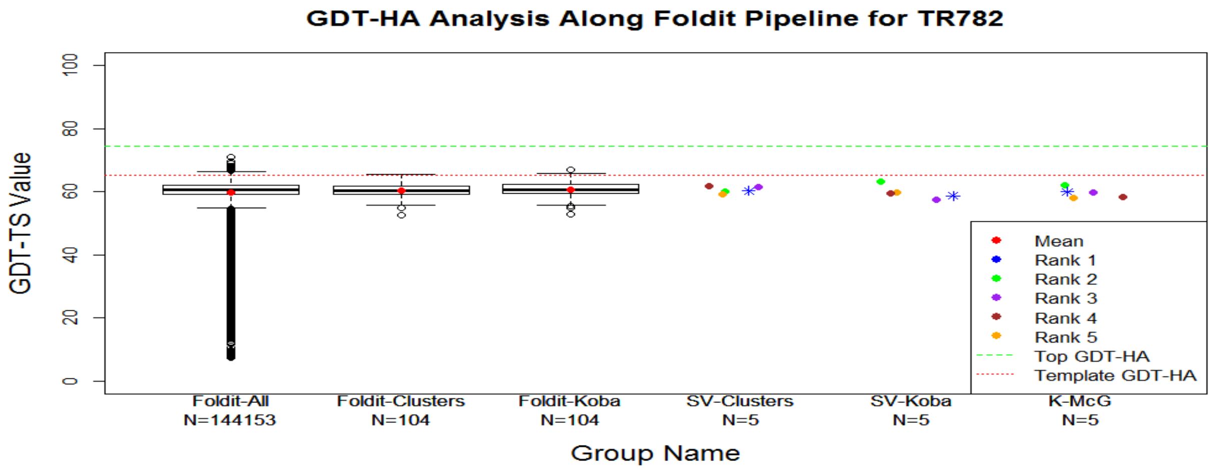
GDT-HA Analysis Along Foldit Pipeline for TR782
GDT-TS Value
Mean
Rank 1
Rank 2
Rank 3
Rank 4
Rank 5
Top GDT-HA
Template GDT-HA
Foldit-All N=144153
Foldit-Clusters N=104
Foldit-Koba N=104
SV-Clusters N=5
SV-Koba N=5
K-McG N=5
Group Name

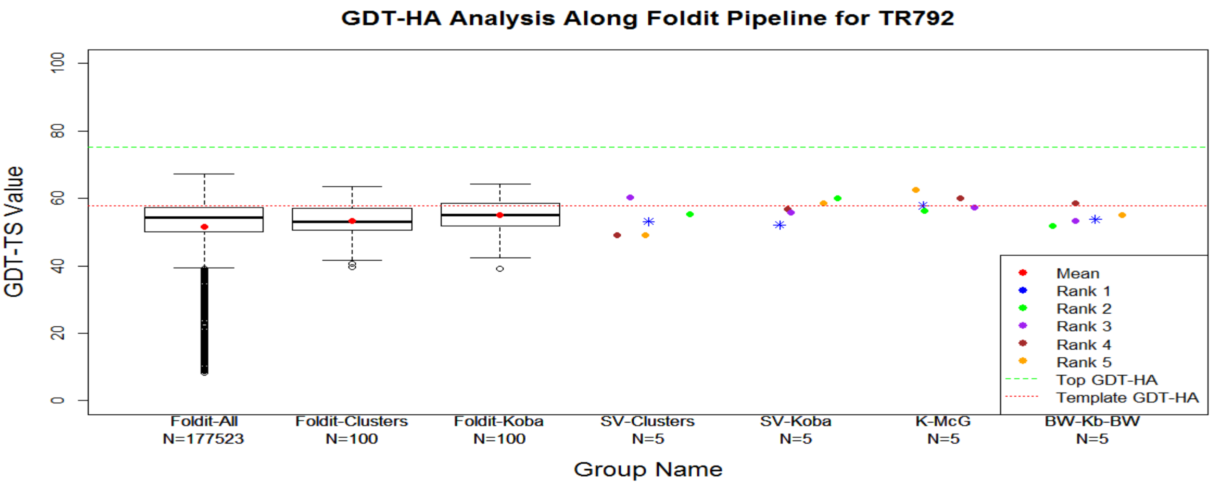
GDT-HA Analysis Along Foldit Pipeline for TR792
GDT-TS Value
0
20
40
60
80
100
Foldit-All N=177523
Foldit-Clusters N=100
Foldit-Koba N=100
SV-Clusters N=5
SV-Koba N=5
K-McG N=5
BW-Kb-BW N=5
Group Name
Mean
Rank 1
Rank 2
Rank 3
Rank 4
Rank 5
Top GDT-HA
Template GDT-HA

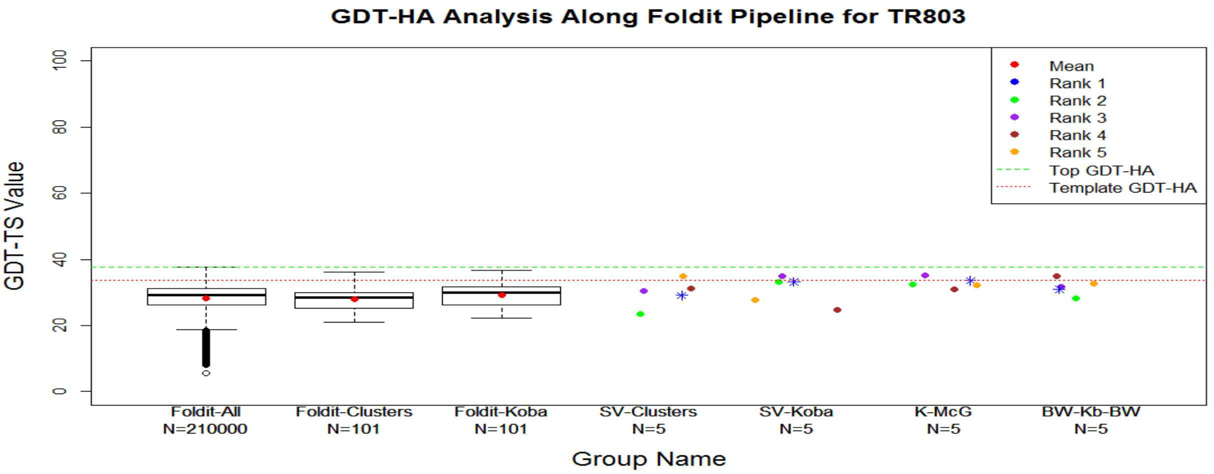
GDT-HA Analysis Along Foldit Pipeline for TR803
GDT-TS Value
0
20
40
60
80
100
Foldit-All N=210000
Foldit-Clusters N=101
Foldit-Koba N=101
SV-Clusters N=5
SV-Koba N=5
K-McG N=5
BW-Kb-BW N=5
Group Name
Mean
Rank 1
Rank 2
Rank 3
Rank 4
Rank 5
Top GDT-HA
Template GDT-HA

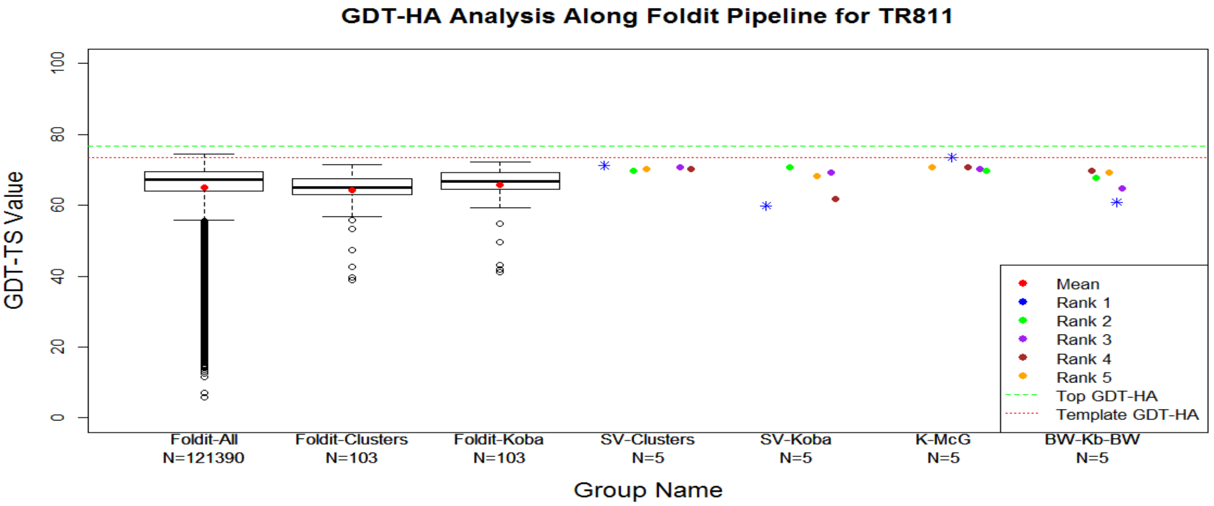
GDT-HA Analysis Along Foldit Pipeline for TR811
GDT-TS Value
0
20
40
60
80
100
Foldit-All N=121390
Foldit-Clusters N=103
Foldit-Koba N=103
SV-Clusters N=5
SV-Koba N=5
K-McG N=5
BW-Kb-BW N=5
Group Name
Mean
Rank 1
Rank 2
Rank 3
Rank 4
Rank 5
Top GDT-HA
Template GDT-HA

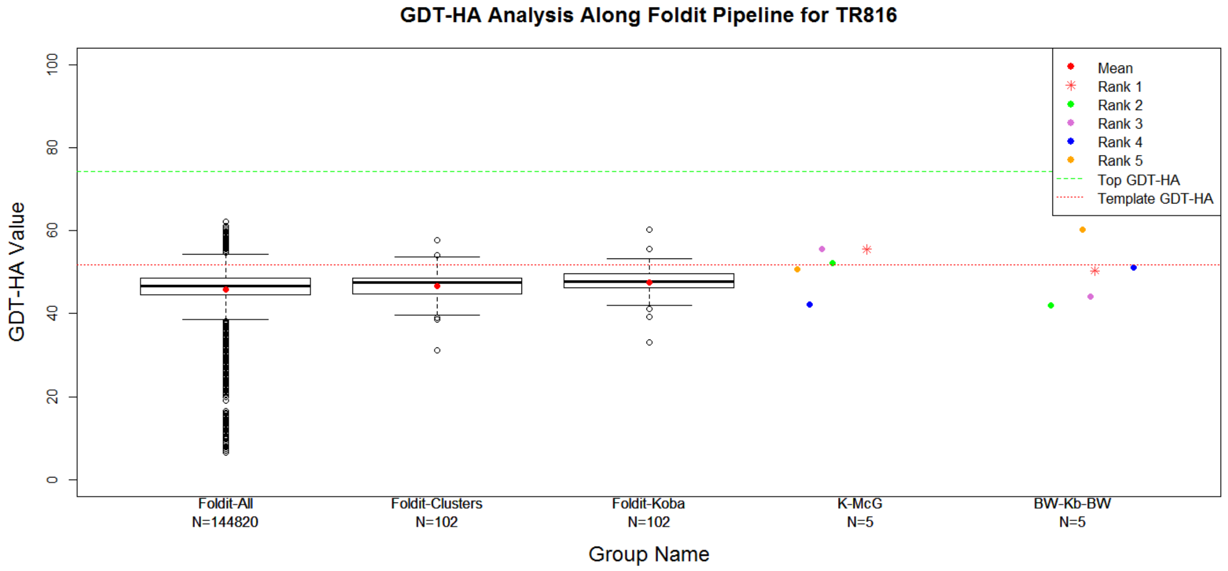
GDT-HA Analysis Along Foldit Pipeline for TR816
GDT-HA Value
Mean
Rank 1
Rank 2
Rank 3
Rank 4
Rank 5
Top GDT-HA
Template GDT-HA
Foldit-All
N=144820
Foldit-Clusters
N=102
Foldit-Koba
N=102
K-McG
N=5
BW-Kb-BW
N=5
Group Name

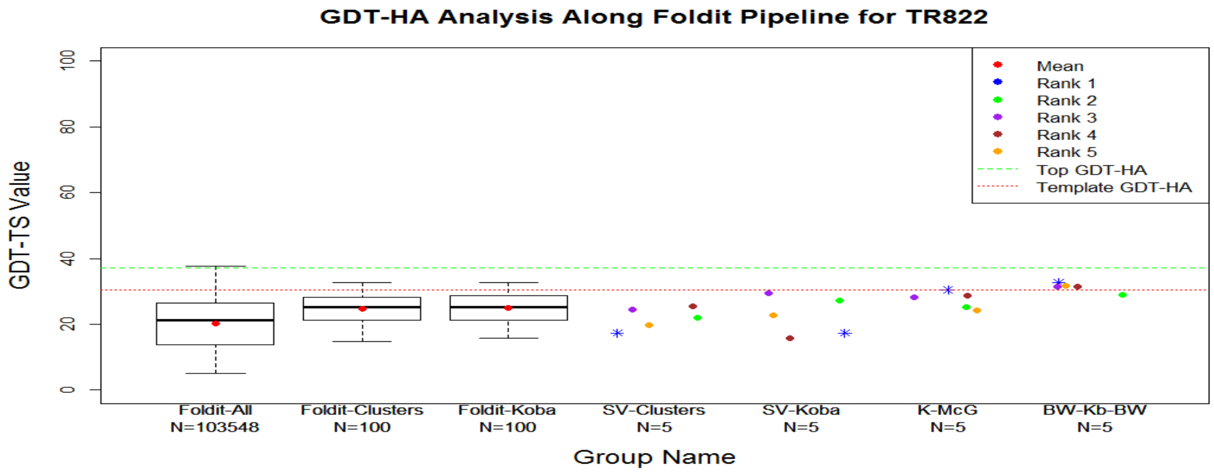
GDT-HA Analysis Along Foldit Pipeline for TR822
GDT-TS Value
Mean
Rank 1
Rank 2
Rank 3
Rank 4
Rank 5
Top GDT-HA
Template GDT-HA
Foldit-All
N=103548
Foldit-Clusters
N=100
Foldit-Koba
N=100
SV-Clusters
N=5
SV-Koba
N=5
K-McG
N=5
BW-Kb-BW
N=5
Group Name

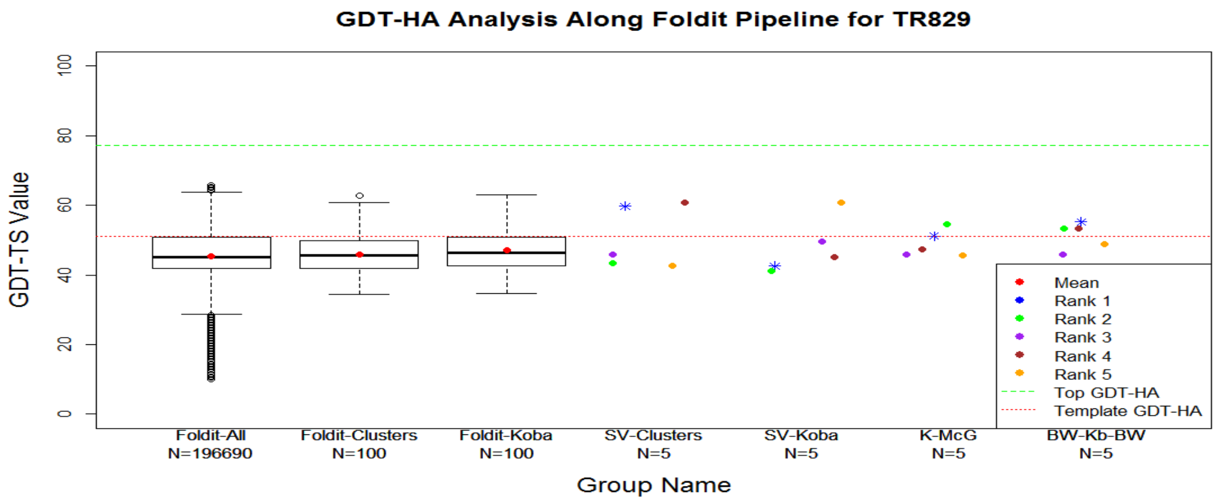
GDT-HA Analysis Along Foldit Pipeline for TR829
GDT-TS Value
Group Name
Foldit-All N=196690
Foldit-Clusters N=100
Foldit-Koba N=100
SV-Clusters N=5
SV-Koba N=5
K-McG N=5
BW-Kb-BW N=5
Mean
Rank 1
Rank 2
Rank 3
Rank 4
Rank 5
Top GDT-HA
Template GDT-HA

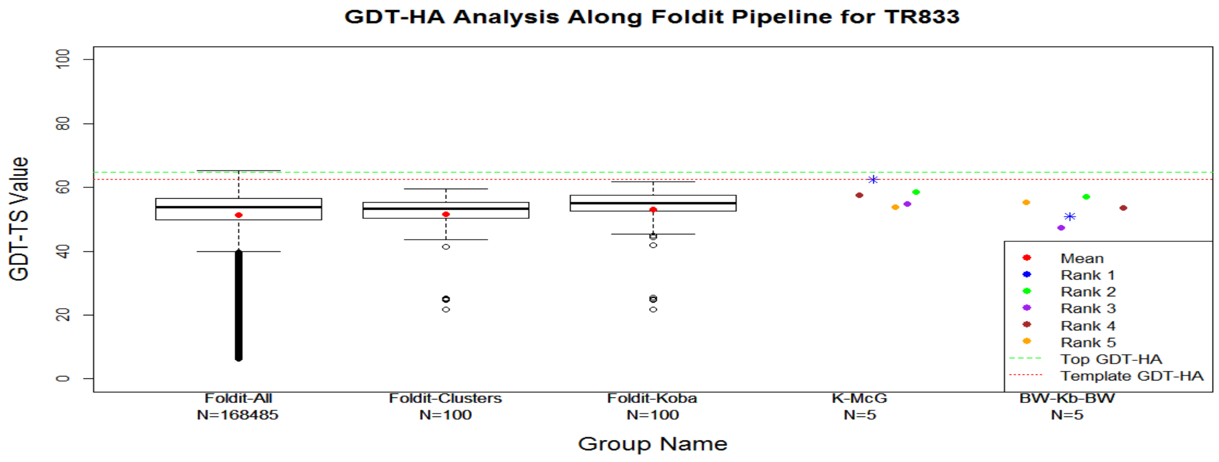
GDT-HA Analysis Along Foldit Pipeline for TR833
GDT-TS Value
Group Name
Foldit-All N=168485
Foldit-Clusters N=100
Foldit-Koba N=100
K-McG N=5
BW-Kb-BW N=5
Mean
Rank 1
Rank 2
Rank 3
Rank 4
Rank 5
Top GDT-HA
Template GDT-HA

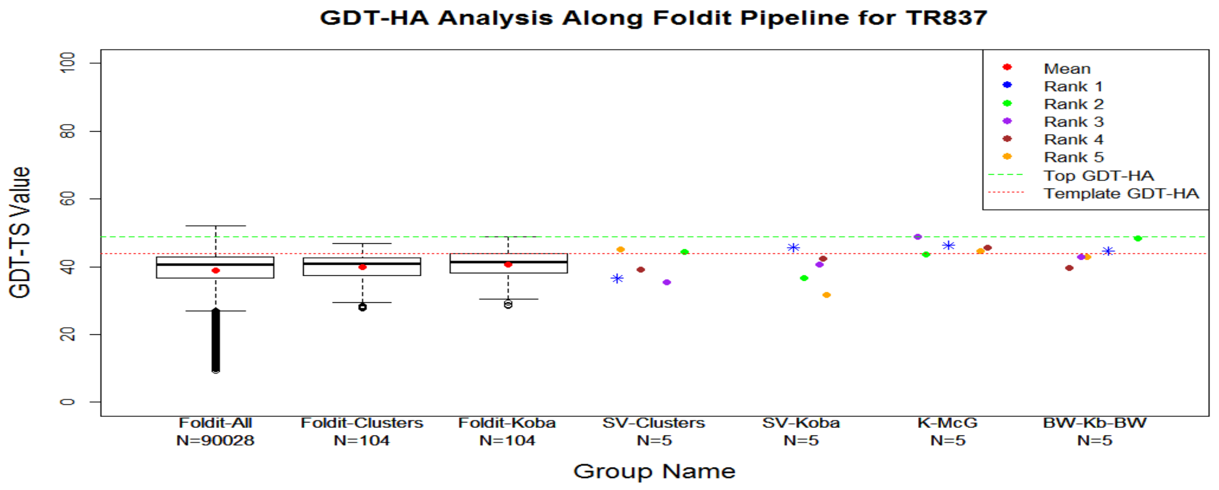
GDT-HA Analysis Along Foldit Pipeline for TR837
GDT-TS Value
Group Name
Foldit-All N=90028
Foldit-Clusters N=104
Foldit-Koba N=104
SV-Clusters N=5
SV-Koba N=5
K-McG N=5
BW-Kb-BW N=5
Mean
Rank 1
Rank 2
Rank 3
Rank 4
Rank 5
Top GDT-HA
Template GDT-HA

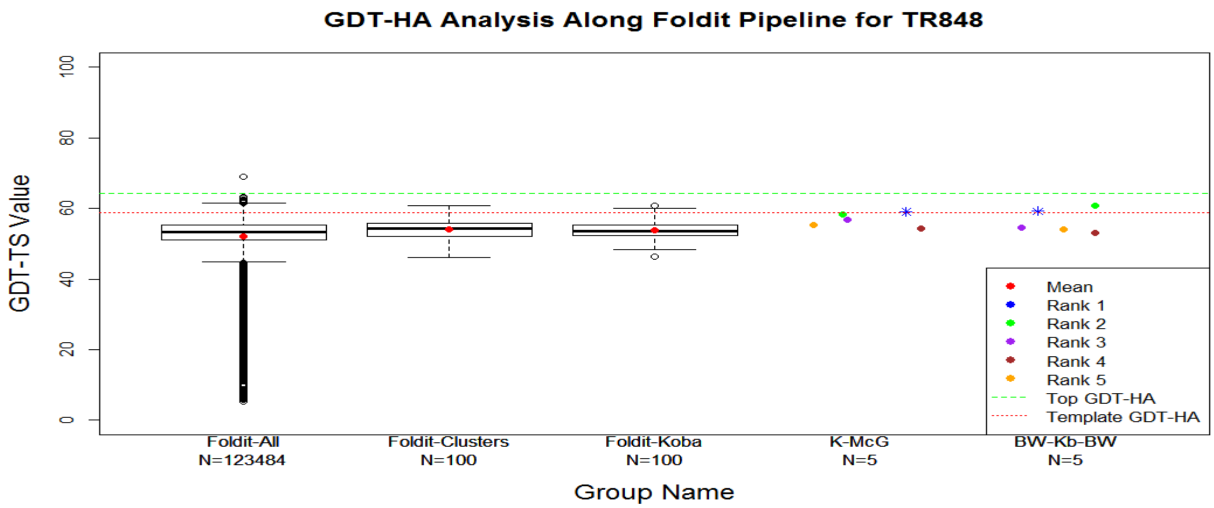
GDT-HA Analysis Along Foldit Pipeline for TR848
GDT-TS Value
Group Name
Foldit-All N=123484
Foldit-Clusters N=100
Foldit-Koba N=100
K-McG N=5
BW-Kb-BW N=5
Mean
Rank 1
Rank 2
Rank 3
Rank 4
Rank 5
Top GDT-HA
Template GDT-HA

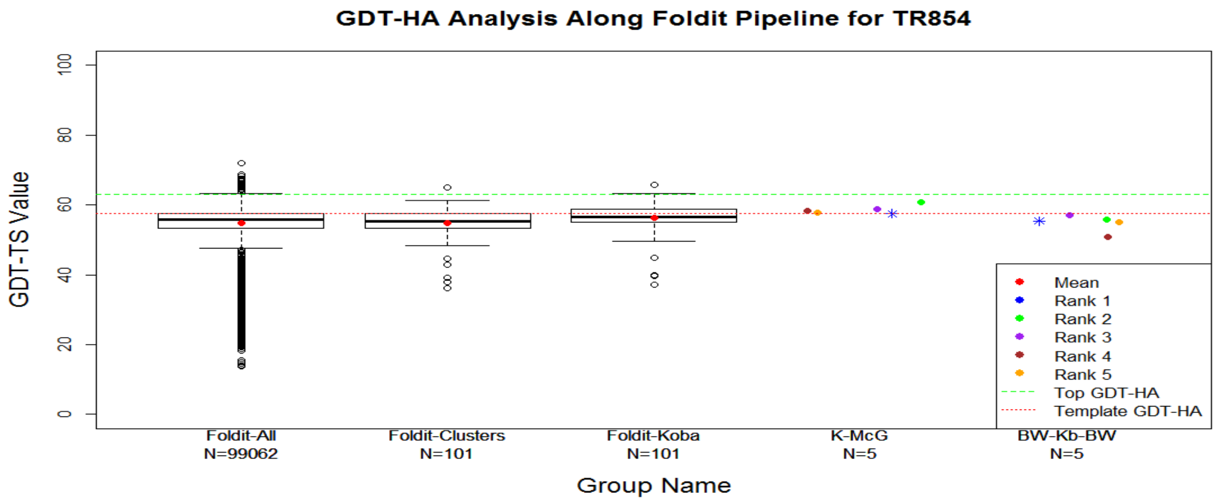
GDT-HA Analysis Along Foldit Pipeline for TR854
GDT-TS Value
Group Name
Foldit-All N=99062
Foldit-Clusters N=101
Foldit-Koba N=101
K-McG N=5
BW-Kb-BW N=5
Mean
Rank 1
Rank 2
Rank 3
Rank 4
Rank 5
Top GDT-HA
Template GDT-HA

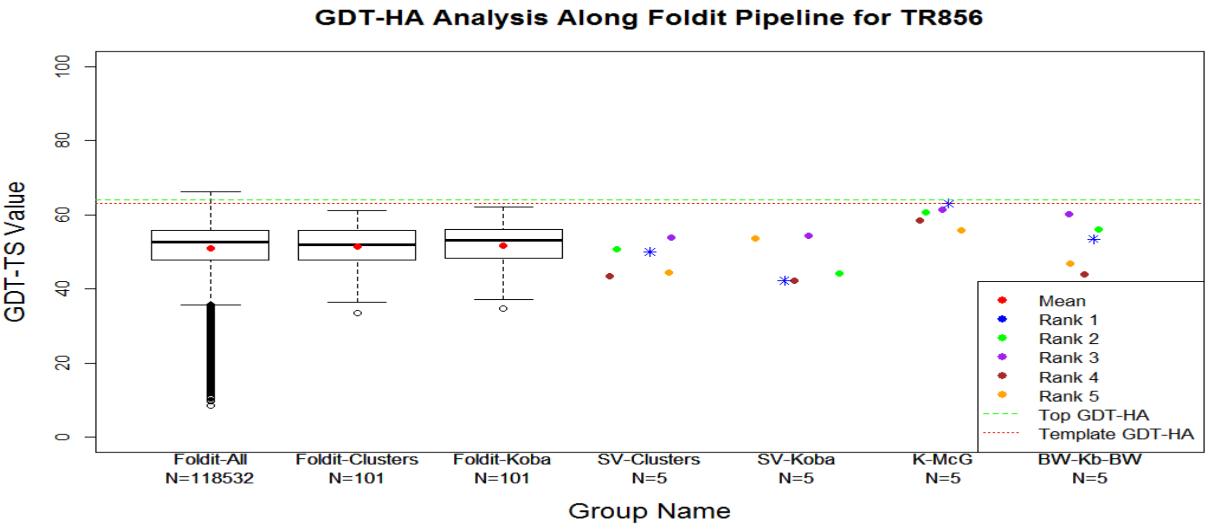


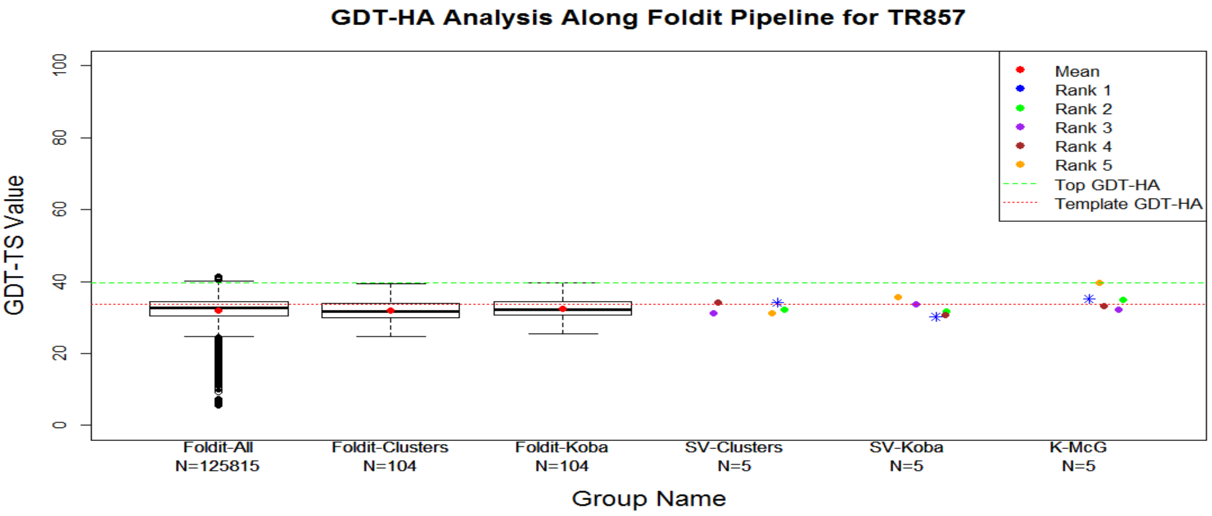


# Large Scale Analysis of WeFold3 Pipelines

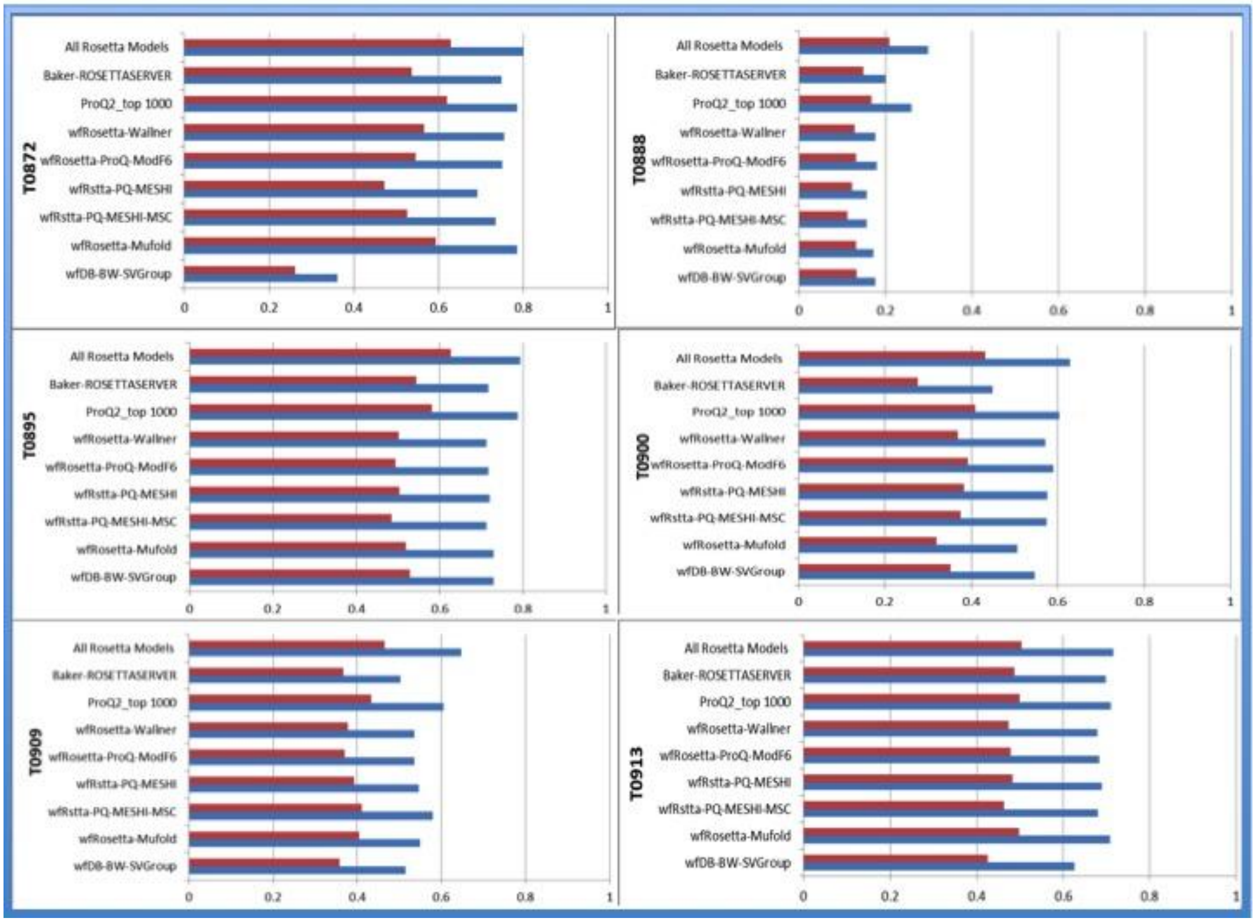
T0872
T0888
T0895
T0900
T0909
T0913
All Rosetta Models
Baker-ROSETTASERVER
ProQ2_top 1000
wfRosetta-Wallner
wfRosetta-ProQ-ModF6
wfRstta-PQ-MESHI
wfRstta-PQ-MESHI-MSC
wfRosetta-Mufold
wfDB-BW-SVGroup
0
0.2
0.4
0.6
0.8
1

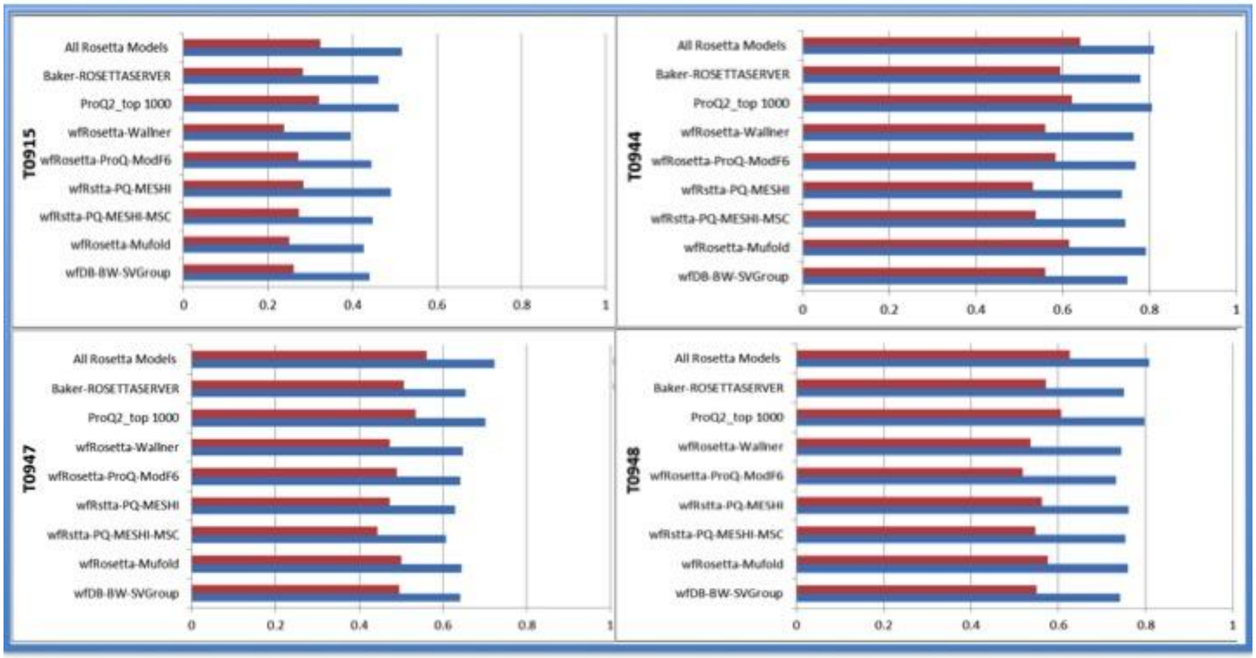
T0915
T0944
T0947
T0948
All Rosetta Models
Baker-ROSETTASERVER
ProQ2_top 1000
wfRosetta-Wallner
wfRosetta-ProQ-ModF6
wfRstta-PQ-MESHI
wfRstta-PQ-MESHI-MSC
wfRosetta-Mufold
wfDB-BW-SVGroup
0
0.2
0.4
0.6
0.8
1

**Figure S3.** Bar plots show the down-selection process across the Rosetta-based pipelines for 10 targets using GDT-HA and GDT-MM. Red bars represent best GDT-HA and blue bars represent best GDT-MM. GDT-MM is a Baker-lab specific metric, where the MAMMOTH alignment algorithm (MM = MAMMOTH) is used for the superposition (slight variations with respect to GDT-TS are based on alignment.) Top bars show best GDT-HA (or MM) among the hundreds of thousands of models generated by Rosetta. Next 2 bars show the best GDT-HA (MM) among the best 5 selected by the BAKER-ROSETTASERVER; next 2 bars show the best GDT-HA (MM) among the one thousand models selected by ProQ2; the remainder bars show the best GDT-HA (MM) among the best 5 selected by the Rosetta-based WeFold groups (one set of bars each.)

Box and whisker plots analysis for the WeFold3 Rosetta-based pipelines:

Column 1: GDT-MM for all hundreds of thousands Rosetta models generated by Baker lab
Column 2: GDT-MM for the Rosetta models in Column 1 were scored using ProQ2 and the top 1000 models were selected. They are represented in this column.
Column 3: GDT-MM for the best five models selected by BAKER-ROSETTASERVER
Column 4: GDT-MM for the best five models selected by *wfRosetta-ProQ-ModF6*
Column 5: GDT-MM for the best five models selected by *wfRosetta-Wallner*
Column 6: GDT-MM for the best five models selected by *wfRosetta-ProQ-MESHI*
Column 7: GDT-MM for the best five models selected by *wfRosetta-ProQ-MESHI-MSC*
Column 8: GDT-MM for the best five models selected by *wfRosetta-ProQ-MESHI*
Column 9: GDT-MM for the best five models selected by *wfRosetta-MUfold*
Column 10: GDT-MM for the best five models selected by *wfDB_BW_SVGroup*

Dashed red line is the GDT-MM of the best model submitted to CASP12 for that target considering all the CASP12 groups

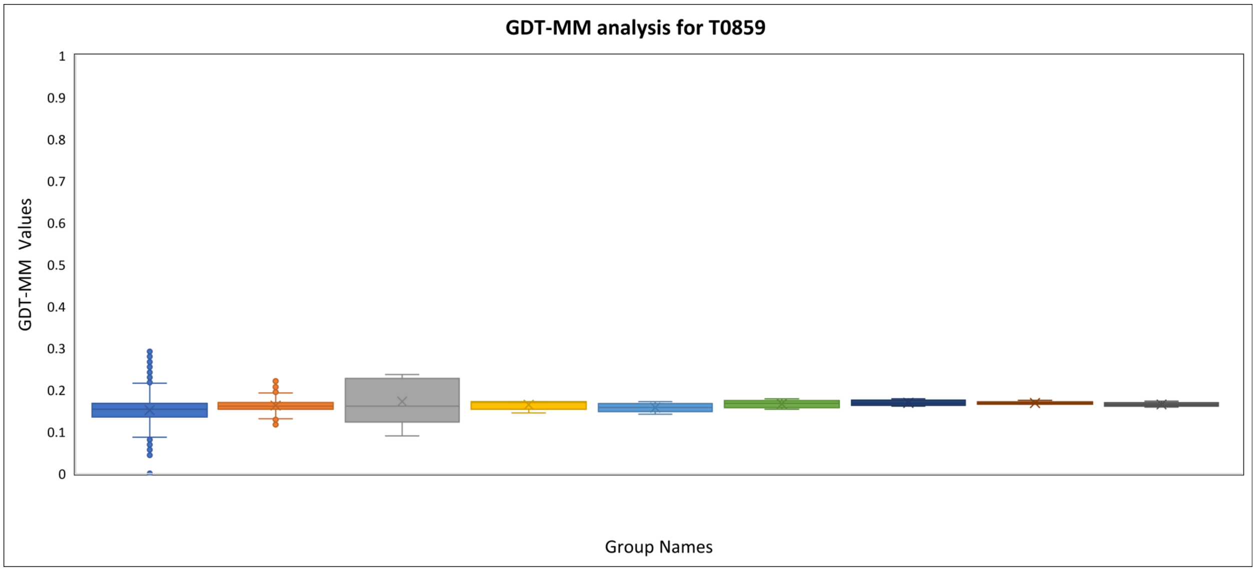

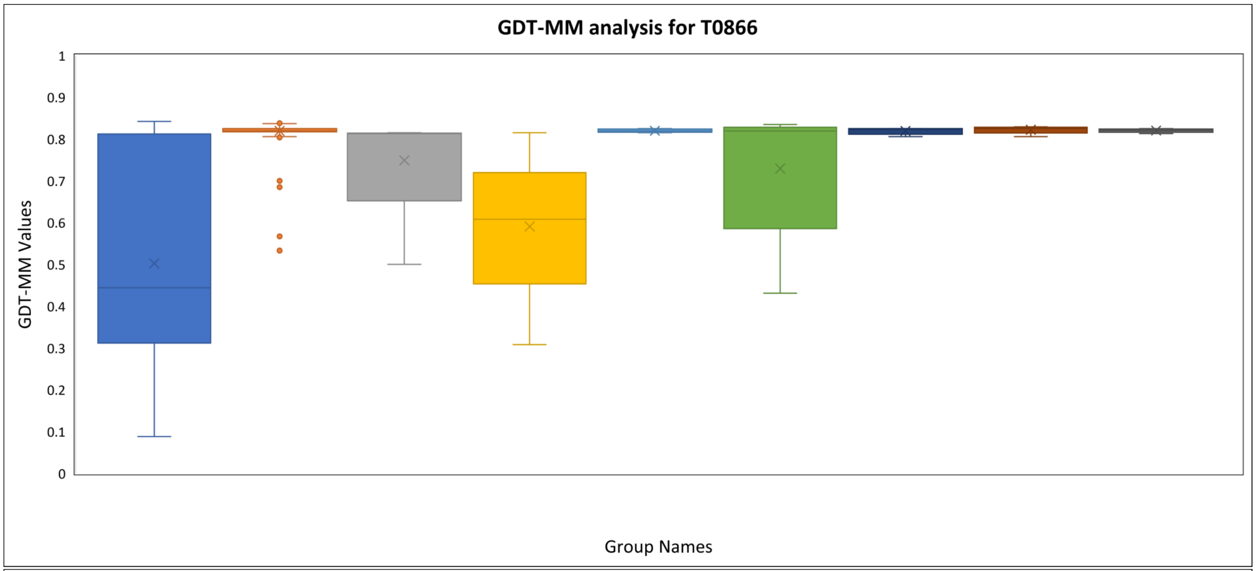
GDT-MM analysis for T0866
1
0.9
0.8
0.7
0.6
0.5
0.4
0.3
0.2
0.1
0
GDT-MM Values
Group Names

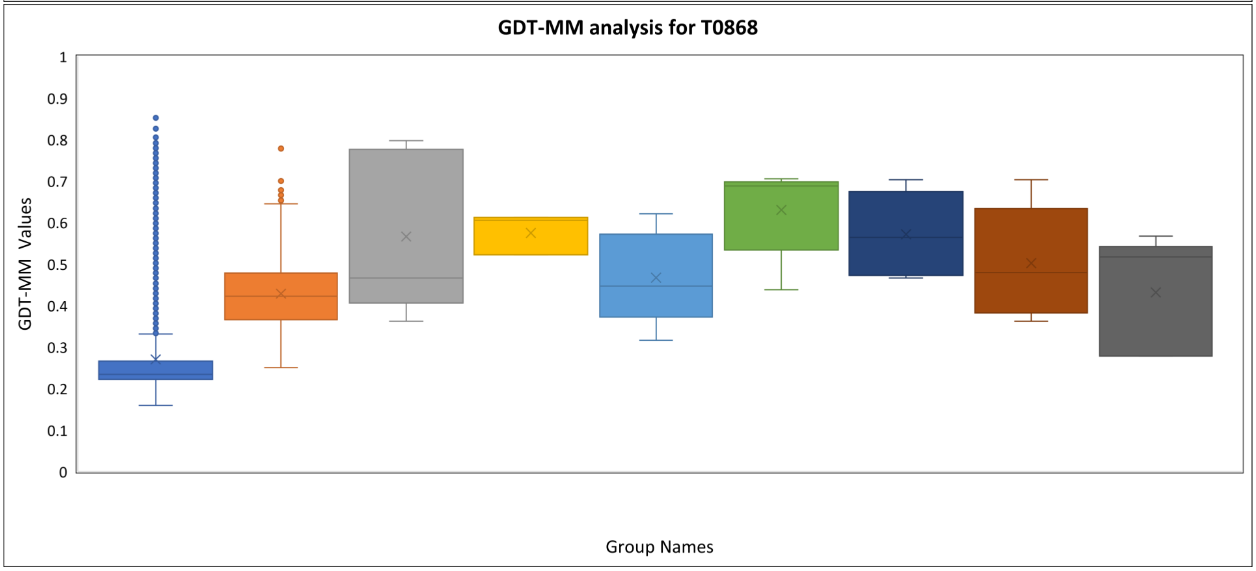
GDT-MM analysis for T0868
1
0.9
0.8
0.7
0.6
0.5
0.4
0.3
0.2
0.1
0
GDT-MM Values
Group Names

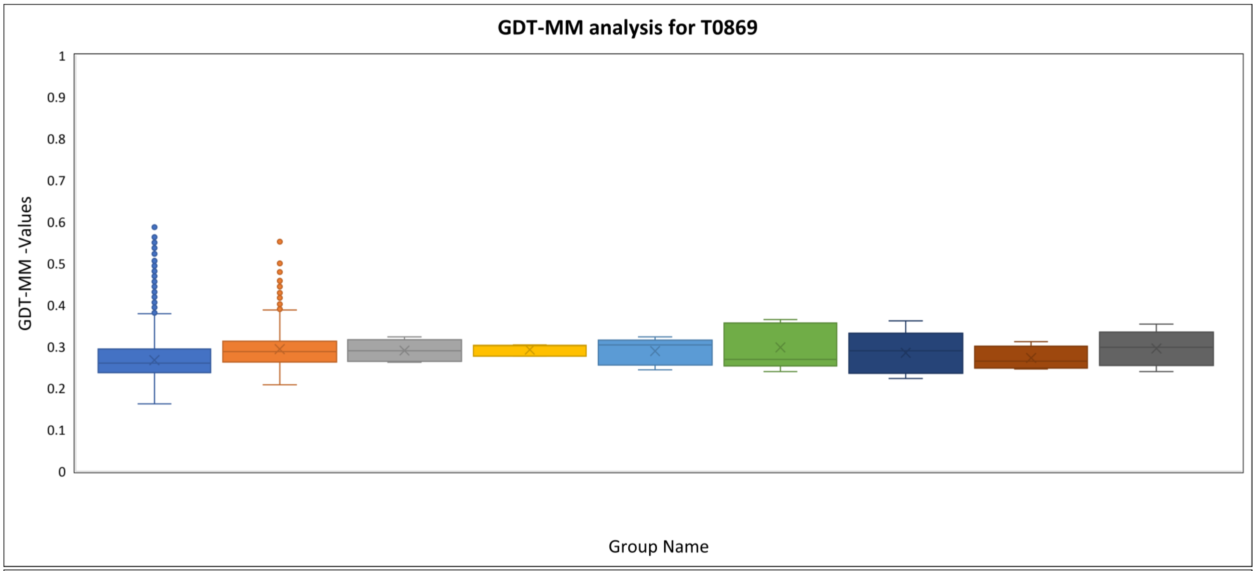
GDT-MM analysis for T0869
GDT-MM -Values
1
0.9
0.8
0.7
0.6
0.5
0.4
0.3
0.2
0.1
0
Group Name

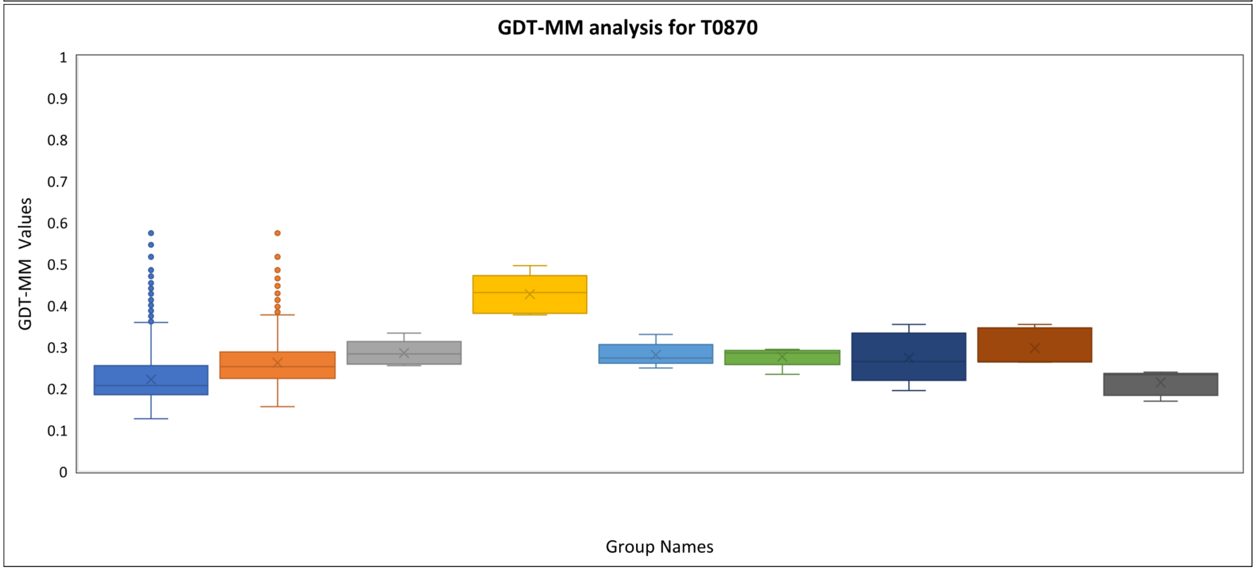
GDT-MM analysis for T0870
GDT-MM Values
1
0.9
0.8
0.7
0.6
0.5
0.4
0.3
0.2
0.1
0
Group Names

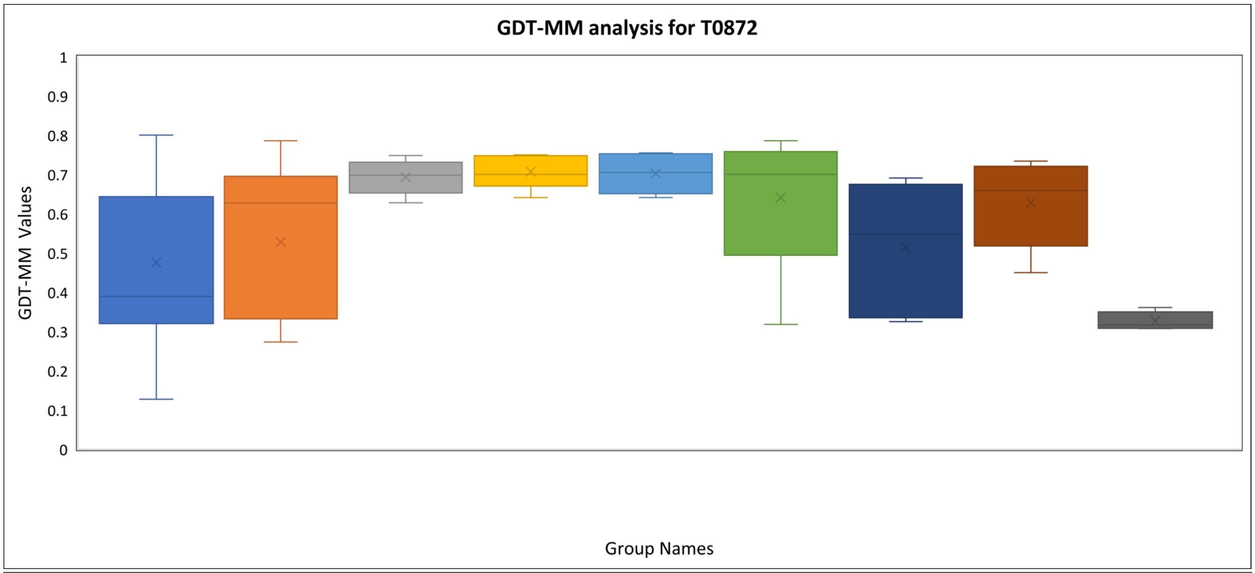
GDT-MM analysis for T0872
GDT-MM Values
1
0.9
0.8
0.7
0.6
0.5
0.4
0.3
0.2
0.1
0
Group Names

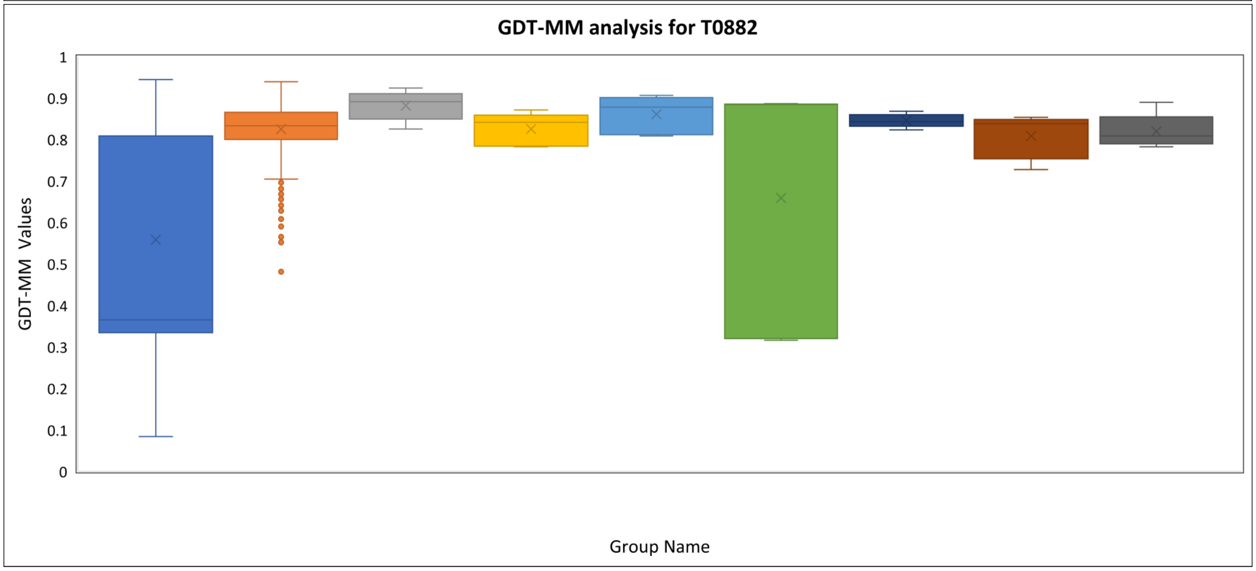
GDT-MM analysis for T0882
GDT-MM Values
1
0.9
0.8
0.7
0.6
0.5
0.4
0.3
0.2
0.1
0
Group Name

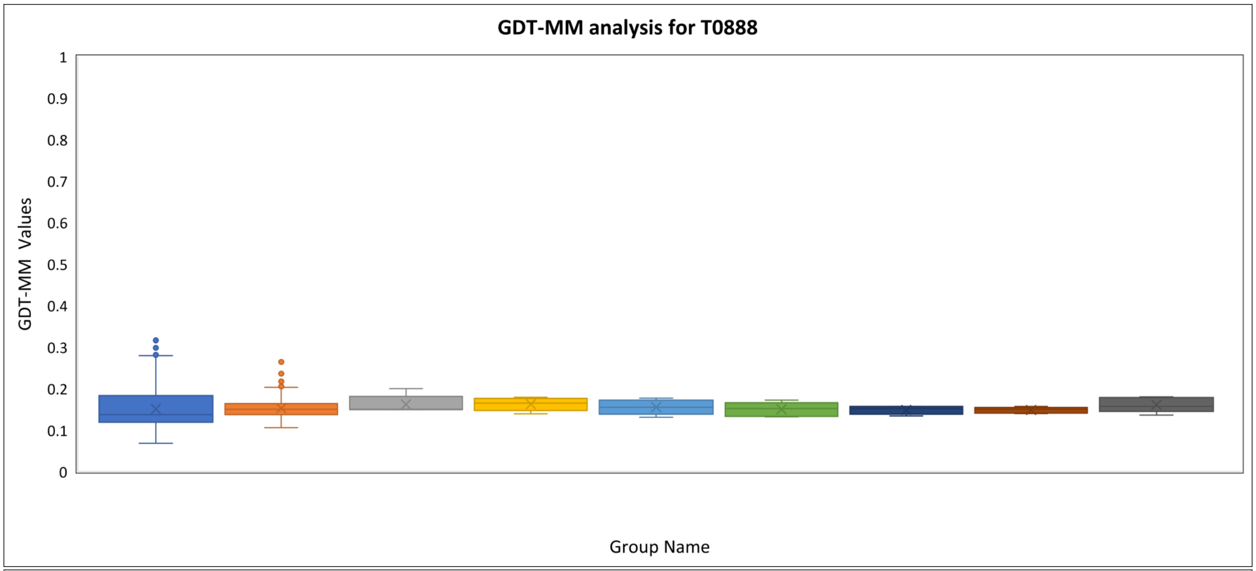
GDT-MM analysis for T0888
GDT-MM Values
1
0.9
0.8
0.7
0.6
0.5
0.4
0.3
0.2
0.1
0
Group Name

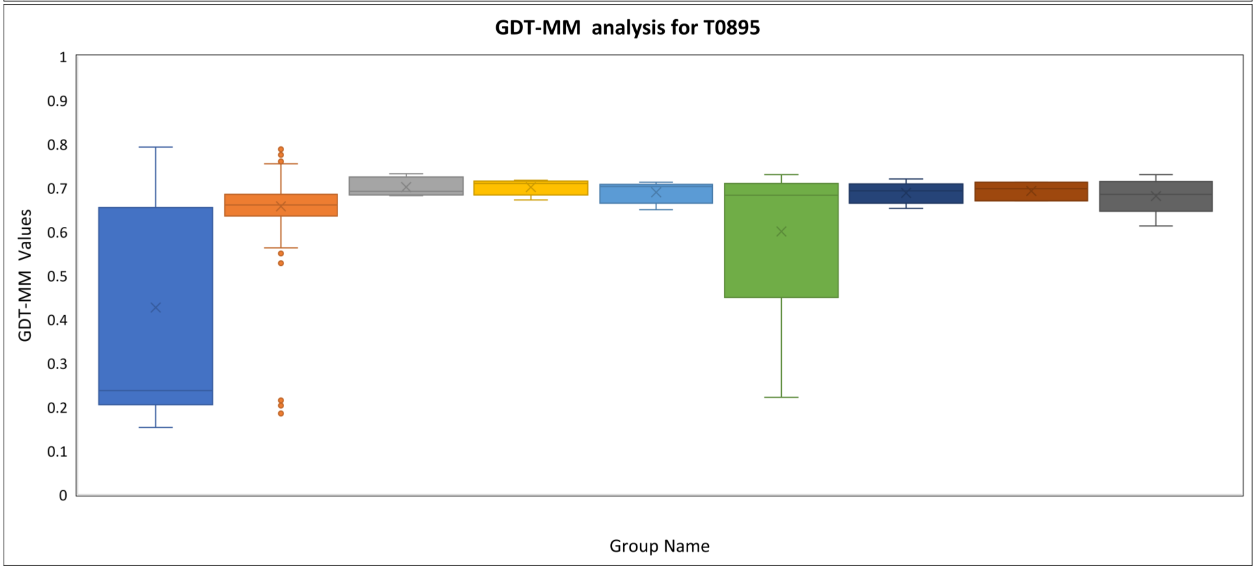
GDT-MM analysis for T0895
GDT-MM Values
1
0.9
0.8
0.7
0.6
0.5
0.4
0.3
0.2
0.1
0
Group Name

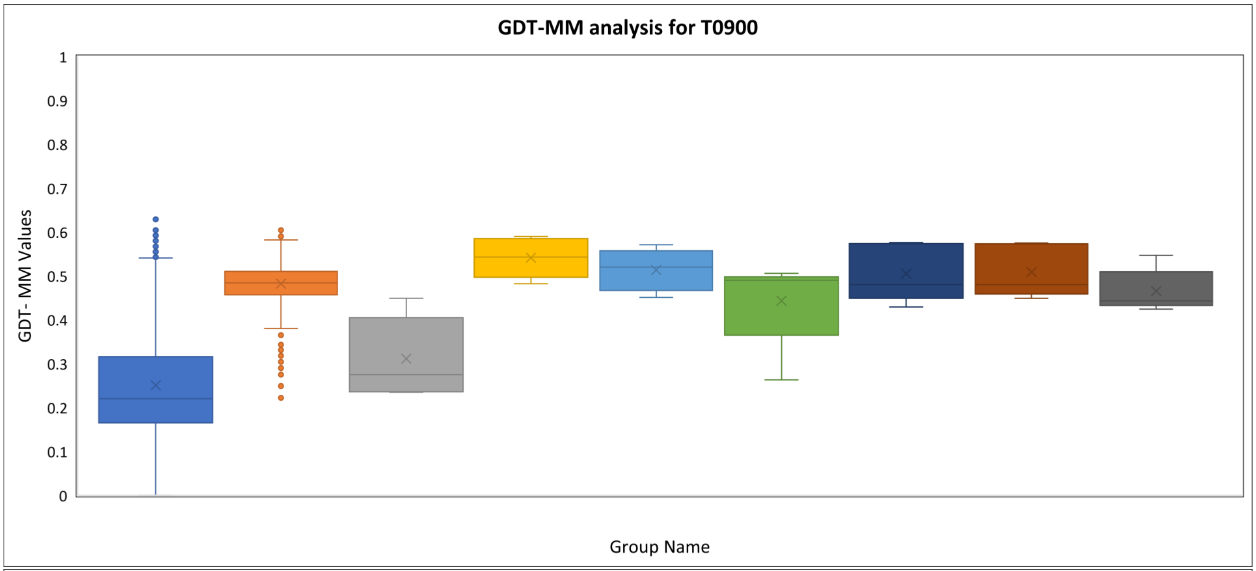
GDT-MM analysis for T0900
GDT- MM Values
1
0.9
0.8
0.7
0.6
0.5
0.4
0.3
0.2
0.1
0
Group Name

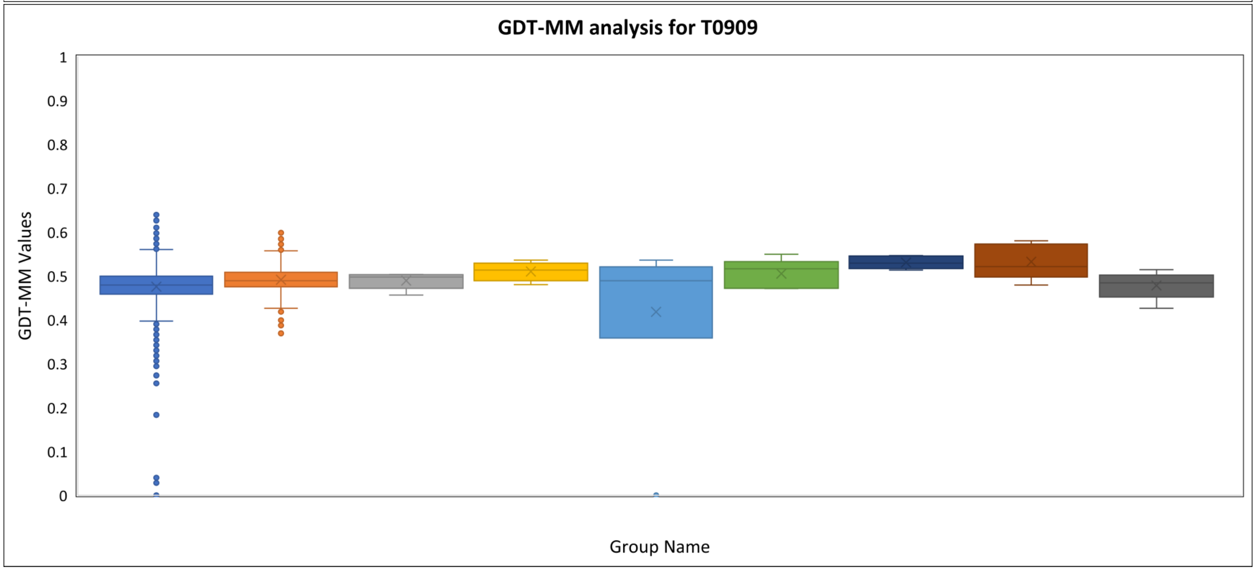
GDT-MM analysis for T0909
GDT-MM Values
1
0.9
0.8
0.7
0.6
0.5
0.4
0.3
0.2
0.1
0
Group Name

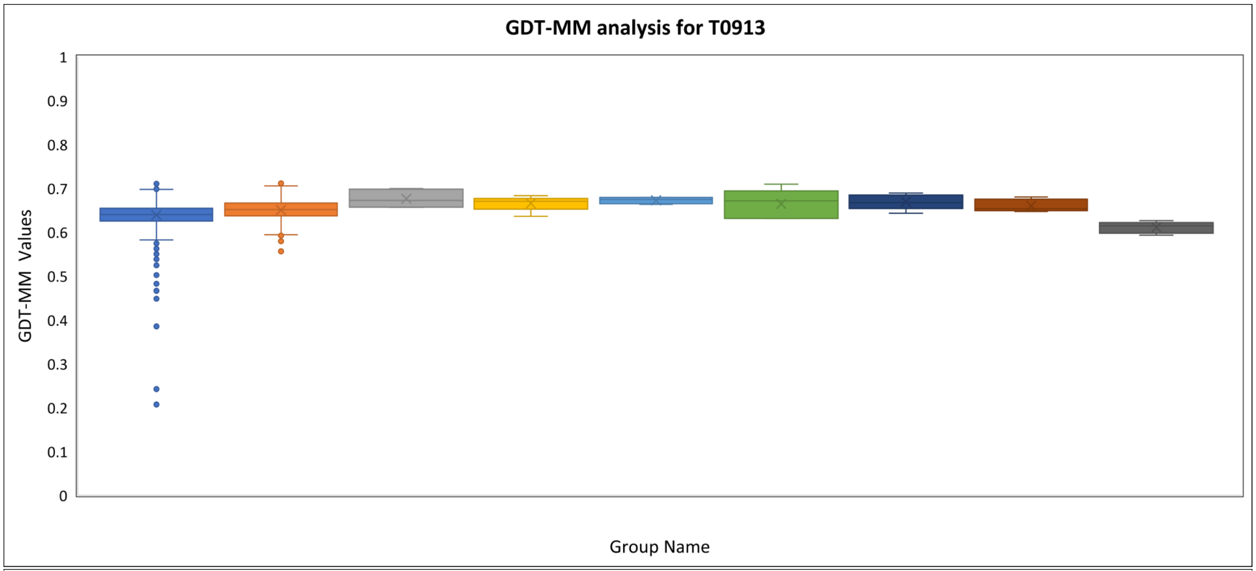
GDT-MM analysis for T0913
GDT-MM Values
1
0.9
0.8
0.7
0.6
0.5
0.4
0.3
0.2
0.1
0
Group Name

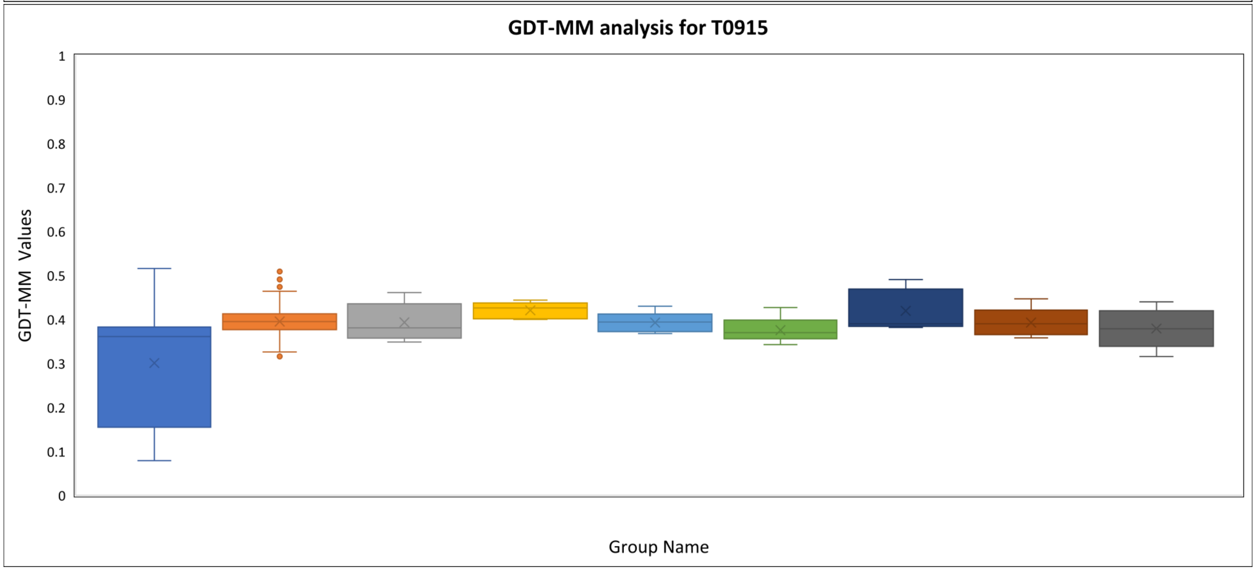
GDT-MM analysis for T0915
GDT-MM Values
1
0.9
0.8
0.7
0.6
0.5
0.4
0.3
0.2
0.1
0
Group Name

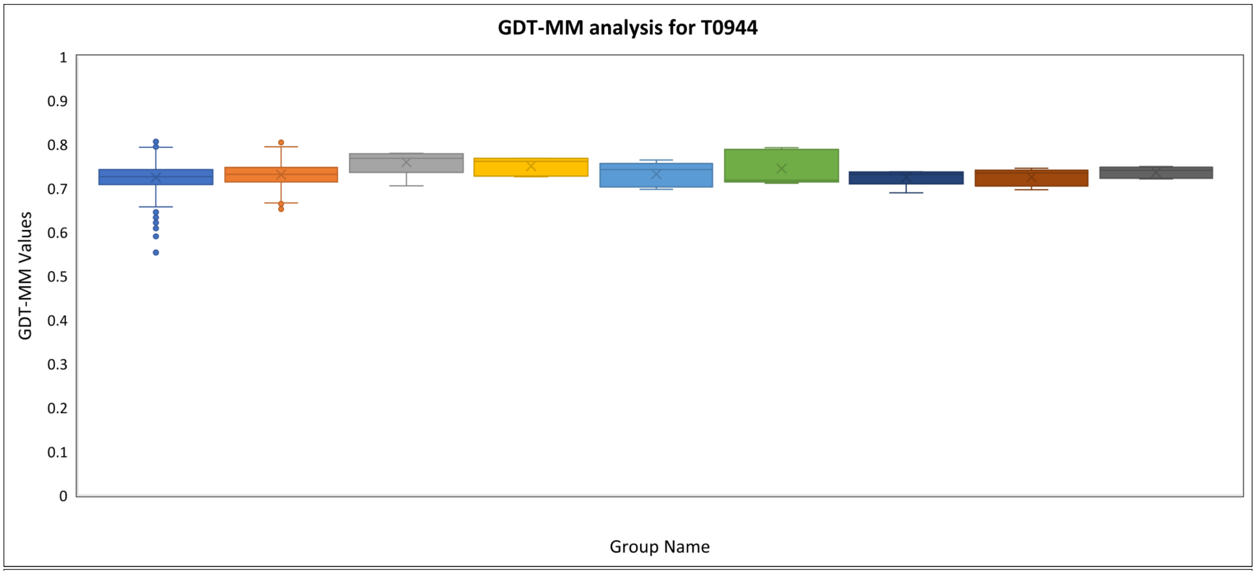
GDT-MM analysis for T0944
GDT-MM Values
1
0.9
0.8
0.7
0.6
0.5
0.4
0.3
0.2
0.1
0
Group Name

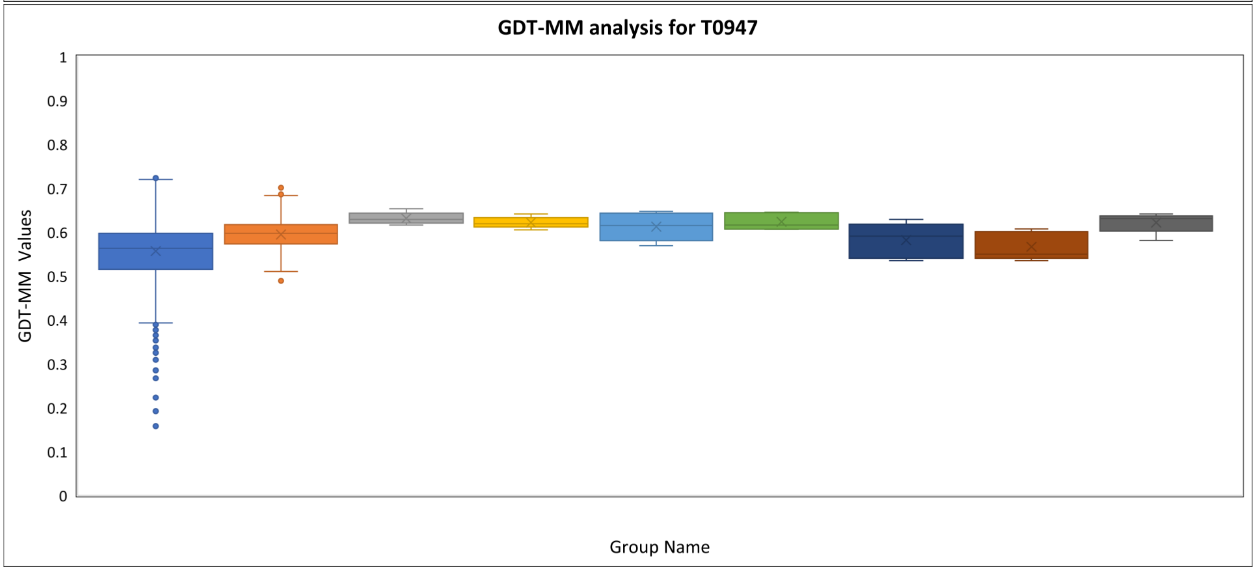
GDT-MM analysis for T0947
GDT-MM Values
1
0.9
0.8
0.7
0.6
0.5
0.4
0.3
0.2
0.1
0
Group Name

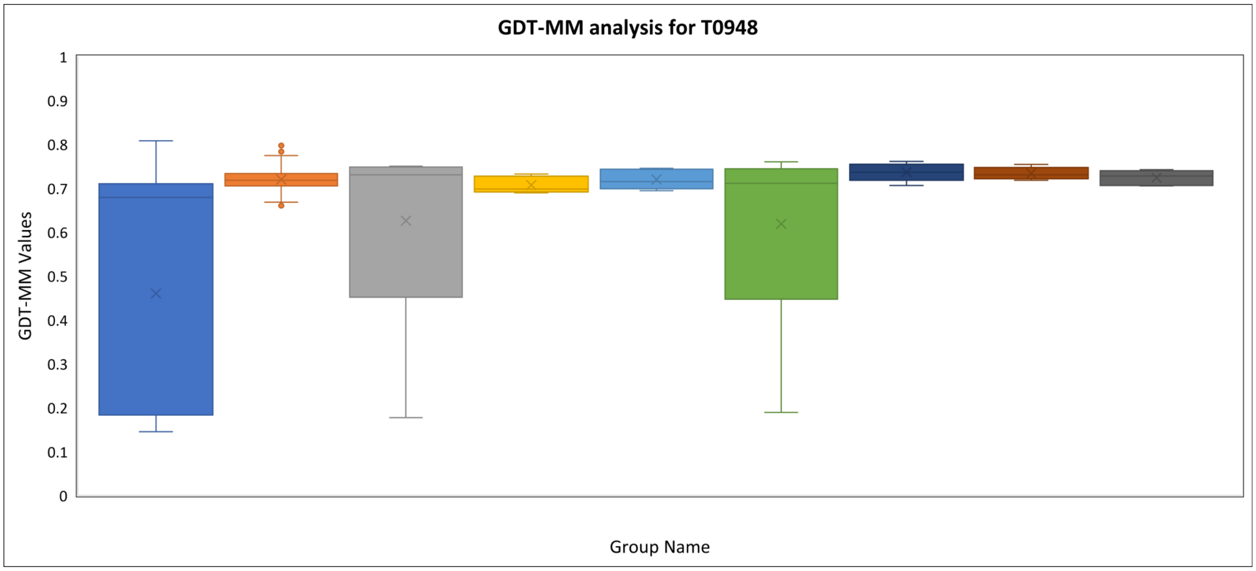


## References:

1. S. Mirzaei, T. Sidi, C. Keasar, and S.Crivelli (2016). "Purely Structural Protein Scoring Functions Using Support Vector Machine and Ensemble Learning." *IEEE/ACM Transactions on Computational Biology and Bioinformatics*, Volume: PP Issue: 99. DOI: 10.1109/TCBB.2016.2602269.
2. Khatib F, DiMaio F, Foldit Contenders Group, Foldit Void Crushers Group, Cooper S, Kazmierczyk M, Gilski M, Krzywda S, Zabranska H, Pichova I, Thompson J, Popović Z, Jaskolski M, Baker D. Crystal structure of a monomeric retroviral protease solved by protein folding game players. Nat Struct Mol Biol 2011;18(10):1175-1177.
3. Leaver-Fay A, Tyka M, Lewis SM, Lange OF, Thompson J, Jacak R, Kaufman K, Renfrew PD, Smith CA, Sheffler W, Davis IW, Cooper S, Treuille A, Mandell DJ, Richter F, Ban YE, Fleishman SJ, Corn JE, Kim DE, Lyskov S, Berrondo M, Mentzer S, Popovic Z, Havranek JJ, Karanicolas J, Das R, Meiler J, Kortemme T, Gray JJ, Kuhlman B, Baker D, Bradley P. ROSETTA3: an object-oriented software suite for the simulation and design of macromolecules. Methods Enzymol 2011;487:545-574.
4. Karplus K. SAM-T08, HMM-based protein structure prediction. *Nucleic Acids Research*. 2009;37(Web Server issue):W492-W497. doi:10.1093/nar/gkp403
5. Heo L, Park H, Seok C. GalaxyRefine: Protein structure refinement driven by side-chain repacking. Nucleic acids research 2013;41(Web Server issue):W384-388.
6. Lee GR, Heo L, and Seok C. Effective protein model structure refinement by loop modeling and overall relaxation, Proteins, *in press* (DOI: 10.1002/prot.24858).
7. MacKerell AD, Bashford D, Bellott M, Dunbrack RL, Evanseck JD, Field MJ, Fischer S, Gao J, Guo H, Ha S, Joseph-McCarthy D, Kuchnir L, Kuczera K, Lau FTK, Mattos C, Michnick S, Ngo T, Nguyen DT, Prodhom B, Reiher WE, Roux B, Schlenkrich M, Smith JC, Stote R, Straub J, Watanabe M, Wiorkiewicz-Kuczera J, Yin D, Karplus M. All-atom empirical potential for molecular modeling and dynamics studies of proteins. J Phys Chem B 1998;102(18):3586-3616.
8. Haberthur U, Caflisch A. FACTS: Fast analytical continuum treatment of solvation. Journal of computational chemistry 2008;29(5):701-715.
9. Kortemme T, Morozov AV, Baker D. An orientation-dependent hydrogen bonding potential improves prediction of specificity and structure for proteins and protein-protein complexes. Journal of

molecular biology 2003;326(4):1239-1259.
10. Yang Y, Zhou Y. Specific interactions for ab initio folding of protein terminal regions with secondary structures. Proteins 2008;72(2):793-803.
11. Canutescu, AA, Shelenkov AA, Dunbrack RL, Jr. A graph-theory algorithm for rapid protein side-chain prediction. Protein Sci. 12:2001-2014, 2003.
12. Ray A, Lindahl E, Wallner B. Improved model quality assessment using ProQ2. BMC bioinformatics (2012) 13 (1), 1.
13. McGuffin,L.J. (2008) The ModFOLD Server for the Quality Assessment of Protein Structural Models. Bioinformatics.24, 586-587.
14. McGuffin,L.J. (2009) Prediction of global and local model quality in CASP8 using the ModFOLD server. Proteins. 77, 185-190.
15. McGuffin, L. J., Buenavista, M. T., & Roche, D. B. (2013) The ModFOLD4 Server for the Quality Assessment of 3D Protein Models. Nucleic Acids Res., 41, W368-72.
16. Buenavista, M. T., Roche, D. B. & McGuffin, L. J. (2012) Improvement of 3D protein models using multiple templates guided by single-template model quality assessment. Bioinformatics. 28, 1851-1857.
17. McGuffin, L.J., Atkins, J., Salehe, B.R., Shuid, A.N. & Roche, D.B. (2015) IntFOLD: an integrated server for modelling protein structures and functions from amino acid sequences. Nucleic Acids Research, In press.
18. McGuffin, L., Roche, D: Rapid Model Quality Assessment for Protein Structure Predictions Using the Comparison of Multiple Models without Structural Alignments. Bioinformatics 2010, 26:182-188.
19. McGuffin,L.J (2007) Benchmarking consensus model quality assessment for protein fold recognition. BMC Bioinformatics. 8, 345
20. Ben-David,M., Noivirt-Brik,O., Paz,A., Prilusky,J., Sussman,J.L. and Levy,Y. (2009) Assessment of CASP8 structure predictions for template free targets, Proteins, 77, 50-65
21. Zhang,Y. and Skolnick,J. (2004) Scoring function for automated assessment of protein structure template quality. Proteins. 57, 702-710.
22. Uziela K., Wallner B. ProQ2: estimation of model accuracy implemented in Rosetta. Bioinformatics. 2016 Jan 5.
23. Levitt M., Gerstein M. A unified statistical framework for sequence comparison and structure comparison. Proc Natl Acad Sci U S A. 1998 May 26;95(11):5913-20.
24. Kryshtafovych A., Barbato A., Monastyrskyy B., Fidelis K., Schwede T., Tramontano A. Methods of model accuracy estimation can help selecting the best models from decoy sets: Assessment of model accuracy estimations in CASP11. Proteins. 2015 Sep 7. doi: 10.1002/prot.24919.
25. Roy A, Kucukural A, Zhang Y. I-TASSER: a unified platform for automated protein structure and function prediction. *Nat Protoc*. **5**(4):725-738 (2010).
26. Xu D, Zhang Y. Ab initio protein structure assembly using continuous structure fragments and optimized knowledge-based force field. *Proteins*; **80**(7):1715-1735 (2012).
27. Zhang Y. Interplay of I-TASSER and QUARK for template-based and ab initio protein structure prediction in CASP10. *Proteins*;**82** Suppl 2:175-187 (2014).
28. Wu ST, Zhang Y. LOMETS: A local meta-threading-server for protein structure prediction. *Nucl Acids Res*;**35**:3375-3382 (2007).
29. Yang, J., et al. Template-based protein structure prediction in CASP11 and retrospect of I-TASSER in the last decade. *Proteins: Structure, Function and Bioinf.* **84** Suppl 1:233-46. doi: 10.1002/prot.24918 (2016)
30. Zhang, W., et al. Integration of QUARK and I-TASSER for Ab Initio Protein Structure Prediction in CASP11. *Proteins: Structure, Function and Bioinf.* **84** Suppl 1:76-86. doi: 10.1002/prot.24930 (2016)
31. Zhang Y, Skolnick J. SPICKER: A clustering approach to identify near-native protein folds. *J*

*Comput Chem*;**25**(6):865-871 (2004).
32. Zhang J, Liang Y, Zhang Y. Atomic-level protein structure refinement using fragment-guided molecular dynamics conformation sampling. *Structure*; **19**(12):1784-1795 (2011).
33. Xue Z, Xu D, Wang Y, Zhang Y. ThreaDom: extracting protein domain boundary information from multiple threading alignments. *Bioinformatics*;**29**(13):i247-i256 (2013).
34. Khoury, G., Liwo, A., Khatib, F., Zhou, H., Chopra, G., Bacardit, J., Bortot, L., Delbem, A.C., Deng, X., Faccioli, R., He, Y., Krupa, P., Li, J., Mozolewska, M., Baker, D., Cheng, J., Floudas, C., Keasar, C., Levitt, M., Popović, Z., Scheraga, H., Skolnick, J., Crivelli, S. & Foldit Players. WeFold: A coopetition for protein structure prediction. *Proteins: Structure, Function, and Bioinformatics* 82(9), 1850-1868 (2014).
35. Kieslich,C.A., Smadbeck,J., Khoury,G.A. & Floudas,C.A. (2015) conSSert: Consensus SVM models for accurate prediction of ordered secondary structure. J. Chem. Inf. Modeling, 56, 455-461.
36. Yang, J., Yan, R., Roy, A., Xu, D., Poisson, J., Zhang, Y. (2015) The I-TASSER Suite: Protein structure and function prediction. *Nat Methods*, 12, 7-8.
37. McGuffin, L.J., Bryson, K., Jones, D.T. (2000) The PSIPRED protein structure prediction server. Bioinformatics, 16, 404-405.
38. Wang,Z., Zhao,F., Peng,J., Xu,J. (2011) Protein 8-class secondary structure prediction using conditional neural fields. *Proteomics*, 11, 3786-3792.
39. Faraggi,E., Zhang,T., Yang,Y., Kurgan,L., Zhou,Y. (2012) SPINE X: Improving protein secondary structure prediction by multi-step learning coupled with prediction of solvent accessible surface area and backbone torsion angles. *J Comp Chem*, 33, 259-67.
40. Kamisetty,H., Ovchinnikov,S, Baker D. (2013) Assessing the utility of coevolution-based residue-residue contact predictions in a sequence- and structure-rich era. *Proc. Natl. Acad. Sci. U.S.A.*, 110, 16674-16679.
41. Söding, J. Protein homology detection by HMM-HMM comparison. Bioinformatics 2005;21:951–960.
42. Remmert,M., Biegert,A., Hauser,A., Söding,J. (2011) HHblits: lightning-fast iterative protein sequence searching by HMM-HMM alignment. *Nat Methods,* 9, 173-175.
43. Suzek BE, Wang Y, Huang H, McGarvey PB, Wu CH, UniProt Consortium. UniRef clusters: a comprehensive and scalable alternative for improving sequence similarity searches. Bioinformatics. 2014 Nov 13:btu739.
44. Eddy SR. Accelerated profile HMM searches. PLoS Comput Biol 2011;7:e1002195.
45. Eddy,S.R. (2009) A new generation of homology search tools based on probabilistic inference. *Genome Inform.*, 23, 205-211.
46. Rhee, Y. M.; Pande, V. S. (2003) Multiplexed-Replica Exchange Molecular Dynamics Method for Protein Folding Simulation. *Biophys. J.*, *84*, 775–786.
47. Nanias, M.; Czaplewski, C.; Scheraga, H. A. (2006) Replica Exchange and Multicanonical Algorithms with the Coarse-Grained UNRES Force Field. *J. Chem. Theory Comput.*, *2*, 513–528.
48. Czaplewski,C., Kalinowski,S., Liwo,A., Scheraga,H.A. (2009) Application of multiplexed replica exchange molecular dynamics to the UNRES force field: Tests with α and α+β proteins. *J Chem. Theory Comput.* 5, 627-640.
49. Liwo,A., Czaplewski,C., Ołdziej,S., Rojas,A.V., Kaźmierkiewicz,R., Makowski,M., Murarka, R.K., Scheraga,H.A. (2008) Simulation of protein structure and dynamics with the coarse-grained UNRES force field. In: *Coarse-Graining of Condensed Phase and Biomolecular Systems.*, ed. G. Voth, Taylor & Francis, Chapter 8, pp. 107-122.
50. He,Y., Xiao,Y., Liwo,A., Scheraga,H.A. (2009) Exploring the parameter space of the coarse-grained UNRES force field by random search: Selecting a transferable medium-resolution force field. *J. Comput. Chem.* 30, 2127-2135.
51. Krupa, P.; Sieradzan, A. K.; Rackovsky, S.; Baranowski, M.; Ołdziej, S.; Scheraga, H. A.; Liwo, A.;

Czaplewski, C. (2013) Improvement of the treatment of loop structures in the UNRES force field by inclusion of coupling between backbone- and side-chain-local conformational states. *J. Chem. Theory Comput.*, *9*, 4620–4632.
52. Sieradzan, A. K.; Krupa, P.; Scheraga, H. A.; Liwo, A.; Czaplewski, C. (2015a) Physics-based potentials for the coupling between backbone- and side-chain-local conformational states in the united residue (UNRES) force field for protein simulations. *J. Chem. Theory Comput.*, *11*, 817–831
53. Sieradzan, A. K. (2015b) Introduction of periodic boundary conditions into UNRES force field. *J. Comput. Chem.*, *36*, 940–946.
54. Kumar, S.; Bouzida, D.; Swendsen, R. H.; Kollman, P. A.; Rosenberg, J. M., The weighted histogram analysis method for free-energy calculations on Biomolecules. I. The method. *J. Comput. Chem.* 1992, *13*, 1011–1021.
55. Liwo,A., Khalili,M., Czaplewski,C., Kalinowski,S., Ołdziej,S., Wachucik,K., Scheraga,H.A. (2007) Modification and optimization of the united-residue (UNRES) potential energy function for canonical simulations. I. Temperature dependence of the effective energy function and tests of the optimization method with single training proteins. *J. Pys. Chem. B* 111, 260-285.
56. Murtagh F. (1985) Multidimensional clustering algorithms. Compstat Lect Vienna Phys Verlag.
57. Rotkiewicz,P., Skolnick,J. (2008) Fast procedure for reconstruction of full-atom protein models from reduced representations. *J. Comput. Chem.*, 29, 1460-1465.
58. Krivov, G. G.; Shapovalov, M. V; Dunbrack, R. L. (2009) Improved Prediction of Protein Side-Chain Conformations with SCWRL4. *Proteins*: *Struct. Funct. Bioinfo.*, *77*, 778–795.
59. Khoury, GA, Smadbeck, J, Kieslich, CA, Koskosidis, AJ, Guzman, YA, Tamamis, P, Floudas, CA (2017) Princeton_TIGRESS 2.0: High refinement consistency and net gains through support vector machines and molecular dynamics in double-blind predictions during the CASP11 experiment. Proteins: Struct., Funct., Bioinf., 85 (6), 1078-1098.
60. http://www.predictioncenter.org/casp11/targetlist.cgi?view=refinement
61. Wang Z, Eickholt J, Cheng J: APOLLO: a quality assessment service for single and multiple protein models. *Bioinformatics*, **27**(12):1715-1716 (2011).
62. Bhattacharya D, Cheng J: 3Drefine: Consistent protein structure refinement by optimizing hydrogen bonding network and atomic‐level energy minimization. *Proteins: Structure, Function, and Bioinformatics*, **81**(1):119-131 (2013).
63. Bhattacharya D, Cheng J: i3Drefine Software for Protein 3D Structure Refinement and Its Assessment in CASP10. *PloS one*, **8**(7):e69648 (2013).
64. Coello CA. Evolutionary multi-objective optimization: a historical view of the field. *Computational Intelligence Magazine, IEEE*, 1 (1): 28–36. (2006).
65. El-Ghazali Talbi. Metaheuristics: from design to implementation. *John Wiley & Sons*. 2009.
66. Hess,B., Bekker,H., Berendsen,H.J.C. & Fraaije,J.G.E.M. (1997). LINCS: A linear constraint solver for molecular simulations. *J. Comp. Chem*. 18, 1463-1472.
67. Pronk S., Páll P., Schulz R., Larsson P., Bjelkmar P., Apostolov R., Shirts M. R., Smith J. C., Kasson P. M. , van der Spoel D., Hess B. & Lindahl E. (2013). GROMACS 4.5: a high-throughput and highly parallel open source molecular simulation toolkit, *Bioinformatics*.
68. Lindorff-Larsen,K., Piana,S., Palmo,K., Maragakis,P., Klepeis,J.L., Dror,R.O. & Shaw,D.E. (2010). Improved side-chain torsion potentials for the Amber ff99SB protein force field. *Proteins.* 78(8), 1950-1958.
69. Tsui, V. & Case, D.A., (2000). Theory and applications of the generalized born solvation model in macromolecular simulations. *Biopolymers.* 56(4), 275-291.
70. Onufriev,A., Bashford,D. & Case,D.A. (2004). Exploring protein native states and large-scale conformational changes with a modified Generalized Born model. *Proteins.* 55(2), 383-394.
71. E Faraggi, A Kloczkowski. A global machine learning based scoring function for protein structure

prediction. *Proteins: Structure, Function, and Bioinformatics* **82** (5), 752-759 (2014)
72. Faraggi, E., & Kloczkowski, A. GENN: a GEneral Neural Network for learning tabulated data with examples from protein structure prediction. In Artificial Neural Networks, 165-178. Springer New York (2015).
73. Pawlowski M, Kozlowski L, Kloczkowski A. MQAPsingle: A quasi single-model approach for estimation of the quality of individual protein structure models. *Proteins*;**84**(8):1021-8 (2016)
74. Kurowski MA, Bujnicki JM. GeneSilico protein structure prediction meta-server. *Nucleic Acids Research,* **1**;31(13):3305-7 (2003)
75. Kabsch W, SANDER C. Dictionary of Protein Secondary Structure - Pattern-Recognition of Hydrogen-Bonded and Geometrical Features. *Biopolymers*;**22**(12):2577-637 (1983)
76. Zhou HY, Zhou YQ. Distance-scaled, finite ideal-gas reference state improves structure-derived potentials of mean force for structure selection and stability prediction. *Protein Science*;**11**(11):2714-26 (2002)
77. Zemla A: LGA: a method for finding 3D similarities in protein structures. *Nucleic Acids Research*, **31**(13):3370-3374 (2003).
78. Eastwood MP, Hardin C, Luthey-Schulten Z, Wolynes PG. Evaluating protein structure-prediction schemes using energy landscape theory. *IBM Journal of Research and Development*;**45**(3-4):475-97 (2001)
79. Goldstein RA, Lutheyschulten ZA, Wolynes PG. Optimal Protein-Folding Codes from Spin-Glass Theory. *Proceedings Of The National Academy Of Sciences Of The United States Of America* **1**;89(11):4918-22 (1992)
80. Ginalski K, Elofsson A, Fischer D, Rychlewski L. 3D-Jury: a simple approach to improve protein structure predictions. *Bioinformatics*;**19**(8):1015-8 (2003).
81. Stumpff-Kane AW, Feig N. A correlation-based method for the enhancement of scoring functions on funnel-shaped energy landscapes. *Proteins-Structure Function and Bioinformatics*;**63**(1):155-64 (2006).
82. Chopra et al. Consistent refinement of submitted models at CASP using a knowledge-based potential. Proteins (2010) vol. 78 (12) pp. 2668-78
83. Bhattacharyya, Moitrayee, Soma Ghosh, and Saraswathi Vishveshwara. "Protein Structure and Function: Looking through the Network of Side-Chain Interactions." *Current Protein and Peptide Science* 17.1 (2016): 4-25
84. N. Kannan and S. Vishveshwara, "Identification of side-chain clusters in protein structures by a graph spectral method," *J Mol Biol,* vol. 292, pp. 441-64, Sep 17 1999.
85. K. V. Brinda and S. Vishveshwara, "A network representation of protein structures: implications for protein stability," *Biophys J,* vol. 89, pp. 4159-70, Dec 2005.
86. S. Chatterjee, M. Bhattacharyya, and S. Vishveshwara, "Network properties of protein-decoy structures," *J Biomol Struct Dyn,* vol. 29, pp. 606-22, 2012.
87. S. Chatterjee, S. Ghosh, and S. Vishveshwara, "Network properties of decoys and CASP predicted models: a comparison with native protein structures," *Mol Biosyst,* vol. 9, pp. 1774-88, Jul 2013.
88. S. Ghosh and S. Vishveshwara, "Ranking the quality of protein structure models using sidechain based network properties," *F1000Research,* vol. 3, 2014.
89. Gadiyaram, V., Ghosh, S. and Vishveshwara, S., 2017. A graph spectral-based scoring scheme for network comparison. *Journal of Complex Networks*, *5*(2), pp.219-244. doi.org/10.1093/comnet/cnw016
90. Ghosh,S., Gadiyaram, V. and Vishveshwara, S., 2017. Validation of Protein Structure Models using Network Similarity Score. *Proteins: Structure, Function, and Bioinformatics*. doi: 10.1002/prot.25332
91. Song, Y., et al. High-resolution comparative modeling with RosettaCM. *Structure*, *21*(10), pp.1735-

1742. (2013)
92. Bradley P, Misura KM, Baker D. Toward high-resolution de novo structure prediction for small proteins. *Science*;**309**:1868–1871. PMID:16166519. DOI:10.1126/science.1113801 (2005)
93. Uziela K, Wallner B. ProQ2: estimation of model accuracy implemented in Rosetta. *Bioinformatics.* PMID: 26733453. DOI: 10.1093/bioinformatics/btv767 (2016)
94. Cao, R., & Cheng, J. Protein single-model quality assessment by feature-based probability density functions. *Scientific reports*, **6**, 23990. (2016).
95. Lee,G.R., Heo,L. & Seok,C. Simultaneous refinement of inaccurate local regions and overall structure in the CASP12 protein model refinement experiment. *Proteins*, *in press.*
